# Genome-Wide Gene-Environment Study Identifies Glutamate Receptor Gene *GRIN2A* as a Parkinson's Disease Modifier Gene via Interaction with Coffee

**DOI:** 10.1371/journal.pgen.1002237

**Published:** 2011-08-18

**Authors:** Taye H. Hamza, Honglei Chen, Erin M. Hill-Burns, Shannon L. Rhodes, Jennifer Montimurro, Denise M. Kay, Albert Tenesa, Victoria I. Kusel, Patricia Sheehan, Muthukrishnan Eaaswarkhanth, Dora Yearout, Ali Samii, John W. Roberts, Pinky Agarwal, Yvette Bordelon, Yikyung Park, Liyong Wang, Jianjun Gao, Jeffery M. Vance, Kenneth S. Kendler, Silviu-Alin Bacanu, William K. Scott, Beate Ritz, John Nutt, Stewart A. Factor, Cyrus P. Zabetian, Haydeh Payami

**Affiliations:** 1 New York State Department of Health Wadsworth Center, Albany, New York, United States of America; 2 Epidemiology Branch, National Institute of Environmental Health Sciences, Research Triangle Park, North Carolina, United States of America; 3 Department of Epidemiology, School of Public Health, University of California Los Angeles, Los Angeles, California, United States of America; 4 Institute of Genetics and Molecular Medicine and The Roslin Institute, University of Edinburgh, Edinburgh, United Kingdom; 5 VA Puget Sound Health Care System and Department of Neurology, University of Washington, Seattle, Washington, United States of America; 6 Virginia Mason Medical Center, Seattle, Washington, United States of America; 7 Booth Gardner Parkinson's Care Center, Evergreen Hospital Medical Center, Kirkland, Washington, United States of America; 8 Department of Neurology, School of Medicine, University of California Los Angeles, Los Angeles, California, United States of America; 9 Nutritional Epidemiology Branch, Divisions of Cancer Epidemiology and Genetics, National Cancer Institute, Bethesda, Maryland, United States of America; 10 Dr. John T. Macdonald Foundation Department of Human Genetics and John P. Hussman Institute for Human Genomics, University of Miami Miller School of Medicine, Miami, Florida, United States of America; 11 Virginia Institute for Psychiatric and Behavioral Genetics, Department of Psychiatry, Virginia Commonwealth University, Richmond, Virginia, United States of America; 12 Department of Environmental Health Sciences, Center for Occupational and Environmental Health, School of Public Health, University of California Los Angeles, Los Angeles, California, United States of America; 13 Department of Neurology, Oregon Health and Sciences University, Portland, Oregon, United States of America; 14 Department of Neurology, Emory University School of Medicine, Atlanta, Georgia, United States of America; The Wellcome Trust Centre for Human Genetics, University of Oxford, United Kingdom

## Abstract

Our aim was to identify genes that influence the inverse association of coffee with the risk of developing Parkinson's disease (PD). We used genome-wide genotype data and lifetime caffeinated-coffee-consumption data on 1,458 persons with PD and 931 without PD from the NeuroGenetics Research Consortium (NGRC), and we performed a genome-wide association and interaction study (GWAIS), testing each SNP's main-effect plus its interaction with coffee, adjusting for sex, age, and two principal components. We then stratified subjects as heavy or light coffee-drinkers and performed genome-wide association study (GWAS) in each group. We replicated the most significant SNP. Finally, we imputed the NGRC dataset, increasing genomic coverage to examine the region of interest in detail. The primary analyses (GWAIS, GWAS, Replication) were performed using genotyped data. In GWAIS, the most significant signal came from rs4998386 and the neighboring SNPs in *GRIN2A*. *GRIN2A* encodes an NMDA-glutamate-receptor subunit and regulates excitatory neurotransmission in the brain. Achieving P_2df_ = 10^−6^, *GRIN2A* surpassed all known PD susceptibility genes in significance in the GWAIS. In stratified GWAS, the *GRIN2A* signal was present in heavy coffee-drinkers (OR = 0.43; P = 6×10^−7^) but not in light coffee-drinkers. The *a priori* Replication hypothesis that “Among heavy coffee-drinkers, rs4998386_T carriers have lower PD risk than rs4998386_CC carriers” was confirmed: OR_Replication_ = 0.59, P_Replication_ = 10^−3^; OR_Pooled_ = 0.51, P_Pooled_ = 7×10^−8^. Compared to light coffee-drinkers with rs4998386_CC genotype, heavy coffee-drinkers with rs4998386_CC genotype had 18% lower risk (P = 3×10^−3^), whereas heavy coffee-drinkers with rs4998386_TC genotype had 59% lower risk (P = 6×10^−13^). Imputation revealed a block of SNPs that achieved P_2df_<5×10^−8^ in GWAIS, and OR = 0.41, P = 3×10^−8^ in heavy coffee-drinkers. This study is proof of concept that inclusion of environmental factors can help identify genes that are missed in GWAS. Both adenosine antagonists (caffeine-like) and glutamate antagonists (*GRIN2A*-related) are being tested in clinical trials for treatment of PD. *GRIN2A* may be a useful pharmacogenetic marker for subdividing individuals in clinical trials to determine which medications might work best for which patients.

## Author Summary

Parkinson's disease (PD), like most common disorders, involves interactions between genetic make-up and environmental exposures that are unique to each individual. Caffeinated-coffee consumption may protect some people from developing PD, although not all benefit equally. In a genome-wide search, we discovered that variations in the glutamate-receptor gene *GRIN2A* modulate the risk of developing PD in heavy coffee drinkers. The study was hypothesis-free, that is, we cast a net across the entire genome allowing statistical significance to point us to a genetic variant, regardless of whether it fell in a genomic desert or an important gene. Fortuitously, the most significant finding was in a well-known gene, *GRIN2A*, which regulates brain signals that control movement and behavior. Our finding is important for three reasons: First, it is a proof of concept that studying genes and environment on the whole-genome scale is feasible, and this approach can identify important genes that are missed when environmental exposures are ignored. Second, the knowledge of interaction between *GRIN2A*, which is involved in neurotransmission in the brain, and caffeine, which is an adenosine-A_2A_-receptor antagonist, will stimulate new research towards understanding the cause and progression of PD. Third, the results may lead to personalized prevention of and treatment for PD.

## Introduction

Common disorders are thought to have both genetic and environmental components. Genome-wide association studies (GWAS) have successfully identified numerous susceptibility loci for many common disorders ranging from behavioral traits such as addiction and substance abuse to infectious and immune-related disorders, age-related neurodegenerative disorders like Alzheimer's, Parkinson's and macular degeneration, metabolic disorders, psychiatric disorders, and many more (for the list and results of over 800 published GWAS see http://www.genome.gov/gwastudies). Despite the success of GWAS, the heritability of common disorders cannot be fully explained by the genes that have been discovered [1]. GWAS are built on the notion that common alleles predispose to common disorders. Rare variants, which are probably responsible for some of the missing heritability, would not have been detected by GWAS. Sequencing the genome and novel analytical methods will help identify the rare variants. Another hiding place for the missing heritability is in interactions. Genes that impact disease through interactions with other genes or environmental factors are not detected by GWAS if their main effects are small. GWAS can only identify genes that exhibit significant main effects; genes that require the interacting factor to be included in the study to show their association with disease are missed. Inclusion of key environmental factors in genome-wide studies is anticipated to be an important next step for deciphering the genetic structure of common multifactorial disorders. Amassing sufficient analytic power for gene-environment studies, however, is a challenge. Power decreases dramatically as a function of frequency of exposure, number of parameters being estimated and sample size. Interaction studies require at least four times the sample size that standard GWAS would require to detect an effect of similar magnitude (reviewed in [2]). Yet, there are fewer datasets with both DNA and environmental exposure data than those with DNA alone, and their sample sizes are often smaller.

Parkinson's disease (PD) is a classic example of a common multifactorial disorder. PD is characterized by neurodegeneration in the substantia nigra that manifests initially as a movement disorder but often leads to cognitive and psychiatric problems as well. PD is progressive and there is no treatment currently available that could prevent or slow disease progression. PD is the second most common neurodegenerative disease after Alzheimer's disease; it affects about 5 million individuals in the 10 most populous nations and is expected to double in frequency by 2030 [3]. Until the 1990's PD was thought to be purely environmental with no genetic component. In the last decade, numerous genes have been identified, some of which can cause PD [4] and others that are susceptibility loci [5]–[10]. There are also compelling data from epidemiology that cigarette smoking and caffeinated-coffee consumption are associated with reduced risk of developing PD [11], [12] and that exposure to environmental neurotoxins is associated with increased risk of developing PD [13]. Thus PD is a strong candidate for studying gene-environment interactions [14].

We conducted a genome-wide association and interaction study (GWAIS) using the joint test [15] for each SNP's marginal association and its interaction with coffee consumption on PD risk, followed by stratified GWAS in heavy and light coffee drinkers (see Analytic Strategy in Materials and Methods section). Our aim was to identify genes that enhance or diminish the protective effect of caffeinated-coffee for use as biomarkers for pharmacogenetic prevention and treatment. Caffeine is an adenosine-receptor antagonist. In animal models of PD, where administration of neurotoxins is used to destroy dopaminergic neurons mimicking PD, caffeine and selective A_2A_-antagonists have been shown to be neuroprotective and attenuate dopamine loss [16]. Selective A_2A_-antagonists have been studied in human clinical trials and found to be safe, well tolerated and to provide symptomatic benefit for persons with PD [17], [18]; however, efficacy has not been high enough in the first generation of the drugs to meet regulatory approval for use as PD drugs. We posit that subsets of patients with certain genotypes may respond well to a given treatment and others may not. When they are combined the average efficacy may be insufficient for regulatory approval, while a subgroup of patients with certain genotype might still benefit substantially. If our prediction is correct, incorporating genetics in clinical trials of PD could revolutionize PD drug development. By examining the interaction of caffeinated-coffee with 811,597 SNPs in a hypothesis-free genome-wide study, we discovered *GRIN2A* as a novel PD modifier gene. *GRIN2A* encodes a subunit of the NMDA-glutamate-receptor which is well known for regulating excitatory neurotransmission in the brain and for controlling movement and behavior.

## Materials and Methods

### Human subjects

Human Subject Committees of the participating institutions approved the study. The Discovery dataset was nested in the NeuroGenetics Research Consortium (NGRC) GWAS which successfully identified known PD genes as well as a novel association with *HLA* [5] which has been widely replicated [10], [19]. For the present GWAIS, Replication samples were provided by PEG [20] (Parkinson, Environment, and Gene), PAGE [21] (Parkinson's, Genes, and Environment from the prospective NIH-AARP Diet and Health Study cohort), and HIHG [9] (Hussman Institute for Human Genomics). Persons with PD had been diagnosed by neurologists using standard criteria [22], control subjects self-reported as not having PD. Cases and controls were all unrelated, non-Hispanic Caucasian, from United States. The NGRC cohort was clinic-based sequentially ascertained patients, PEG and PAGE were community-based incident cases, HIHG was clinic-based and self-referral cases. The numbers of cases/controls with genotype, coffee/caffeine and key clinical and demographic data were NGRC = 1458/931, PEG = 280/310, PAGE = 525/1474, HIHG = 209/133 (Table S1).

### Coffee/caffeine

NGRC, PEG and HIHG had collected lifetime caffeinated-coffee consumption data, measured as cups per day multiplied by the number of years of consumption (ccy) [12], [23]. PAGE had daily mg caffeine intake from all caffeine-containing drinks and foods for 12 months prior to enrollment (1995–1996) and only incident PD cases diagnosed after 1997 were included in the analysis [24]. Despite the variation in data collection, results were consistent across studies, corroborating robustness of the interaction between coffee/caffeine and *GRIN2A*. We could not, and did not, attempt to distinguish the bioactive ingredient in caffeinated-coffee. Although caffeine has been shown to be neuroprotective, there may be other ingredients in caffeinated-coffee that may affect disease pathogenesis. To classify coffee/caffeine intake, each dataset was treated separately according to the measurements available. The median ccy or mg was determined for controls within each dataset (excluding those with zero intake) and used as the cut-off for heavy drinkers (>median) vs. light drinkers (0 to ≤median). The median was 67.5 ccy for NGRC, 74.0 ccy for PEG, 70.0 ccy for HIHG, and 237.8 mg/day for PAGE. For coffee dose, quartiles were defined for each dataset using the full range from zero to maximum intake in controls. Results shown for NGRC, PEG and HIHG are based on lifetime caffeinated-coffee consumption. Truncating coffee use at age-at-onset or age-at-diagnosis in patients did not affect the results. To assess the effects of caffeinated tea and soda, we performed sensitivity analysis in NGRC dataset. Caffeinated soda and tea were commonly and equally consumed by heavy and light coffee drinkers (soda: 80% in both heavy and light drinkers; caffeinated tea: 66% in heavy coffee drinkers and 61% in light coffee drinkers). We repeated GWAIS and stratified GWAS with caffeinated soda and tea as covariates. We also explored association of caffeinated tea and soda with PD expecting an inverse association if caffeine were the bioactive ingredient in coffee.

### Genotyping

The source of DNA was whole blood for NGRC and HIHG, saliva for PAGE, and whole blood (all PD and half of controls) or saliva (half of controls) for PEG. NGRC was genome-wide genotyped using Illumina HumanOmni1-Quad_v1-0_B array and achieved 99.92% call rate and 99.99% reproducibility. GWAS genotyping and statistical quality control (QC) have been published [5]. 811,597 SNPs (excluding Y chromosome SNPs because they are not amenable to sex adjustment) passed GWAS QC and were included in GWAIS. Replication groups genotyped *GRIN2A*_rs4998386. Only one SNP was genotyped for replication; we have no other undisclosed replication results. PEG and HIHG used ABI TaqMan assay-by-design (C__28018721_20), PAGE used Sequenom and all achieved call rates of 96%–99%.

### Analytic strategy

The first step was to test the hypothesis that the effect of coffee on PD risk is affected by a gene; ie, test statistical interaction between SNPs and coffee genome-wide. Theoretically, a test of SNP*coffee interaction would have been suitable; however, a pure test of interaction has low power; reportedly, it requires more than four times the sample size that GWAS would require to detect a main effect of similar size (reviewed in [2]). We chose the joint test of SNP main effect and its interaction with coffee as proposed by Kraft et al [15]. We call the test GWAIS for genome-wide *association* and *interaction* study. The main advantage of the joint test is that it does test for interaction and it has more power than pure interaction test when there is a modest SNP marginal effect. Next we performed stratified GWAS in heavy and light drinkers to gain insight to where the interaction signal was coming from and to formulate a hypothesis for replication. We then replicated the top signal and performed pooled analysis. Methods for meta-analysis of the joint test are available [25], [26]; however, since we had individual level data we pooled the datasets.

### Statistical analysis

#### Quality control for GWAIS and stratified GWAS in Discovery (NGRC)

The genome-wide genotypes for NGRC had been cleaned previously for GWAS using standard rigorous measures [5]. We had identified two significant principal components (PC1, PC2) marking Jewish/non-Jewish ancestry and European countries of origin [5]. Sex was a significant variable, because PD affects more men than women and our data has a significant gender disparity (Table S1). Controls were older than patients at age at onset, which was by design to minimize the chances that controls were too young to have developed the disease. Nevertheless, we controlled for age at enrolment both for patients and controls to avoid confounding by age-related factors. We examined coffee consumption and the most significant SNP for potential variation by disease related variables, recruitment sites, and ethnic and geographic origins of subjects (Table S2). Smoking was a potential confounder because it is correlated with coffee use and is an independent inverse risk factor of PD. Thus we repeated all analyses with smoking included as a covariate in the model (Table S3 without smoking as covariate, Table S4 with smoking as covariate). We also repeated analyses with caffeinated tea and soda in the model (Table S5). For details on how the data on tea, soda and smoking were collected in NGRC, see [12].

#### GWAIS in Discovery

811,597 SNP genotypes [5] and lifetime caffeinated-coffee consumption data [12] from 1458 persons with PD and 931 controls from NGRC were analyzed. We tested the following models: [SNP+coffee+SNP*coffee+covariate vs. coffee+covariate] henceforth referred to as [SNP+SNP*coffee] joint test [15]. Critically, the main effect of coffee on PD risk was present in both models being compared thus we controlled for coffee in the test. This model conducts a 2 degrees of freedom (df) joint test of SNP marginal effect and its interaction with coffee on PD risk [15]. Sex, age, PC1 and PC2 were included as covariates. We used likelihood ratio test statistics as implemented in PLINK [27], and tested the Dominant, Additive and Recessive modes of inheritance. GWAIS was repeated once with the addition of smoking as a covariate, and again by addition of caffeinated tea and soda as covariates.

#### Stratified GWAS in Discovery

There were 512 cases and 387 controls in the heavy coffee drinking group and 946 cases and 544 controls in the light coffee drinking group. We tested association of 811,597 SNPs with PD in each group using standard GWAS with 1 df. We used PLINK [27] and adjusted for age, sex, PC1 and PC2. Stratified GWAS were repeated with smoking, caffeinated soda and caffeinated tea added as covariates (Tables S4 and S5).

#### Replication

Based on the main finding in Discovery, we specified the replication hypothesis a-priori as follows: “Among heavy coffee drinkers, carriers of rs4998386_T allele have a lower risk of PD than carriers of rs4998386_CC genotype”. Note that we were using the GWAIS as a means to identify the genes that might enhance the inverse association of coffee with PD with the goal of carrying the discovery forward to pharmacogenetic studies. Hence, the replication hypothesis was framed as specified. We used three datasets for replication PEG [20], PAGE [21], and HIHG [9]. We tested between-study heterogeneity using Breslow-Day test statistics. There was no heterogeneity in coffee use, in rs4998386_CC or in rs4998386_TC genotype frequencies, but rs4998386_TT frequency, which is quite rare, varied significantly across studies. There were a total of 26 cases and 26 controls with rs4998386_TT genotype in Discovery and Replication combined. We found no trend in rs4998386_TT subject characteristics that could help pinpoint the source of heterogeneity (Table S6). Given the unanticipated heterogeneity in rs4998386_TT, we performed genotype-specific analysis (comparing TC to CC, excluding TT) as well as Dominant and Additive models which included TT. Categorical data were analyzed using logistic regression in SAS (version 9.2) and were adjusted for age and sex, and for source of data when data were pooled. Age at onset was analyzed as a continuous variable using linear regression in SAS.

#### Significance

P values were two-sided for Discovery, one-sided for Replication given the clear directional prior hypothesis [28], and two sided when Discovery and Replication were pooled. There is no agreed-upon significance threshold for GWAIS. The Bonferroni corrected threshold for all 811,597 SNPs on the array is P<6.4×10^−8^. However, not all 811,597 SNPs are independent due to linkage disequilibrium (LD). SimpleM [29] provides a sound Bonferroni-based multiple testing correction method for GWAS based on the estimated number of independent tests, allowing for marker-to-marker LD. It was shown to be the best approximation for permutation, which is computationally prohibitive for GWAS. Using simpleM we calculated the number of independent SNPs genome-wide for NGRC as M_eff_ = 430,151; thus the Bonferroni corrected threshold for independent tests was P<1.16×10^−7^.

#### Imputation

We used IMPUTE v2 [30] with HapMap and 1000 Genomes genotypes combined as reference data to infer genotypes for SNPs that were not originally included on the Illumina OMNI-1 array and thus not genotyped in the NGRC dataset. 2,710,971 SNPs were imputed with high reliability (information score ≥0.95) and had MAF>0.01, increasing the genomic coverage to 3,522,568 SNPs total (genotyped and imputed). We performed GWAIS and stratified GWAS for the *GRIN2A* region (Chromosome 16, 97 Mb–102 Mb) using genotype probability data (dose 2-0) in R software http://www.r-project.org/.

#### Linkage disequilibrium

Linkage disequilibrium and haplotype blocks were estimated using the Haploview software [31]. Haplotype analysis was performed using hapstat adjusting for sex and age [32].

#### Copy number variations (CNV)

We used Golden Helix SNP Variation Suite version 7.2.3 (http://www.goldenhelix.com/) and PennCNV [33] to explore for deletions or duplications in the *GRIN2A* region. Golden Helix found no CNVs; PennCNV identified two controls with CNVs, which even if confirmed to be real, would not affect the results of the study.

#### Data access

NGRC genome-wide genotypes, phenotype and environmental data are available at www.ncbi.nlm.nih.gov/gap study accession number phs000196.v1.p1.

## Results

### GWAIS in Discovery

**Figure 1:**
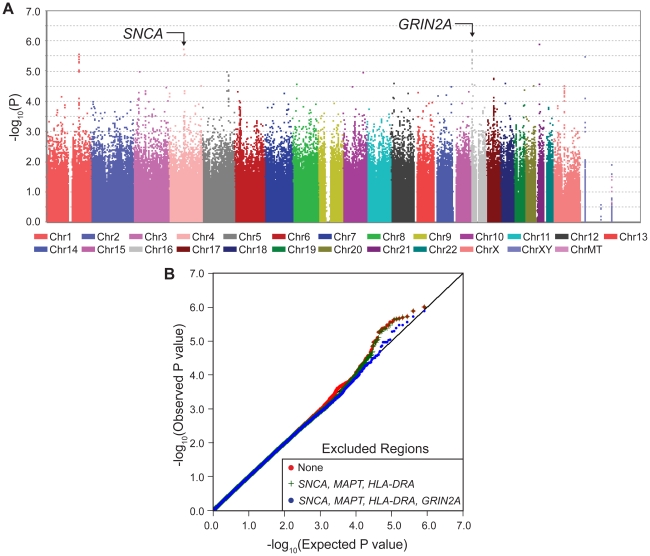
Manhattan Plot and QQ Plot of GWAIS. Panel A depicts the Manhattan plot for the GWAIS (joint test of association and interaction with coffee, 2df, adjusted for sex, age, PC1 and PC2). The novel spike on chromosome 16 corresponds to 12 *GRIN2A* SNPs that were genotyped. Imputed SNPs achieved P_2df_<5×10^−8^ (see Table 4). Additive model is shown here, Dominant and Recessive are in Figure S1. Dominant and Additive models yielded similar results for top hits (see Table 1). Panel B is the QQ plot where the observed P values (red line) are plotted against the expected P values under no association (straight black line). The plots were made first by including all genotyped SNPs (red), then excluding those in the *SNCA*, *HLA* and *MAPT* regions (green) and finally by excluding *GRIN2A* (blue).

The most significant result was the novel appearance, on the Manhattan plot (Figure 1A, Figure S1), of a block of linked SNPs which map to the *GRIN2A* gene on chromosome 16 (Figure S2). This locus had not been detected in PD GWAS previously because its main effect is modest. However, when considered in the context of interaction with coffee, *GRIN2A* surpassed all known PD-associated genes in significance including *SNCA* which has been the strongest association with PD in GWAS. The signal for known PD genes were driven only by their main effects with no evidence for interaction (P_interaction_ = 0.5–0.7); whereas the signals for PD-associated SNPs in *GRIN2A* were enhanced by SNP*coffee interaction (P_interaction_∼10^−3^). The quantile-quantile (QQ) plot of the expected vs. observed genome-wide P values (Figure 1B) is also evidence for the impact of *GRIN2A* on PD risk.

**Table 1:** *GRIN2A* was the most significant signal in GWAIS.

								Dominant					Additive				
					MAF	MAF	HWE	SNP		Interaction		2df	SNP		Interaction		2df
CHR	Gene	SNP	BP	Minor/Major Allele	Case	Control	P	OR (SE)	P	OR (SE)	P	P	OR (SE)	P	OR (SE)	P	P
**Novel PD-association identified via interaction with coffee**																	
16	*GRIN2A*	rs4998386	9978046	T/C	0.08	0.12	0.54	0.82 (0.12)	0.17	0.49 (0.11)	2×10^−3^	1×10^−6^	0.84 (0.11)	0.19	0.50 (0.11)	1×10^−3^	1×10^−6^
16	*GRIN2A*	rs17569693	9987686	G/A	0.08	0.12	0.54	0.79 (0.11)	0.10	0.51 (0.12)	4×10^−3^	1×10^−6^	0.80 (0.10)	0.09	0.54 (0.12)	5×10^−3^	2×10^−6^
16	*GRIN2A*	rs8043728	10003004	T/C	0.09	0.12	0.88	0.82 (0.12)	0.16	0.53 (0.12)	5×10^−3^	8×10^−6^	0.82 (0.11)	0.13	0.57 (0.12)	0.01	8×10^−6^
16	*GRIN2A*	rs8056683	10052710	T/C	0.09	0.13	1.00	0.82 (0.12)	0.17	0.52 (0.12)	3×10^−3^	4×10^−6^	0.83 (0.11)	0.14	0.55 (0.11)	4×10^−3^	4×10^−6^
16	*GRIN2A*	rs9927926	10057405	C/T	0.09	0.13	1.00	0.82 (0.12)	0.17	0.52 (0.12)	3×10^−3^	4×10^−6^	0.83 (0.11)	0.14	0.55 (0.11)	4×10^−3^	4×10^−6^
16	*GRIN2A*	rs17671033	10068727	A/G	0.09	0.13	1.00	0.85 (0.12)	0.24	0.50 (0.11)	2×10^−3^	3×10^−6^	0.85 (0.11)	0.20	0.53 (0.11)	2×10^−3^	4×10^−6^
16	*GRIN2A*	rs9933111	10072100	G/A	0.09	0.13	0.88	0.85 (0.12)	0.24	0.49 (0.11)	2×10^−3^	2×10^−6^	0.85 (0.11)	0.20	0.53 (0.11)	2×10^−3^	2×10^−6^
16	*GRIN2A*	rs13331465	10077968	T/C	0.09	0.13	0.88	0.85 (0.12)	0.23	0.49 (0.11)	2×10^−3^	2×10^−6^	0.85 (0.11)	0.19	0.53 (0.11)	2×10^−3^	2×10^−6^
16	*GRIN2A*	rs13336632	10078155	C/A	0.09	0.13	0.88	0.85 (0.12)	0.23	0.49 (0.11)	2×10^−3^	2×10^−6^	0.85 (0.11)	0.19	0.52 (0.11)	2×10^−3^	2×10^−6^
16	*GRIN2A*	rs1448270	10082819	T/G	0.09	0.13	0.77	0.86 (0.12)	0.29	0.49 (0.11)	2×10^−3^	5×10^−6^	0.86 (0.11)	0.24	0.53 (0.11)	2×10^−3^	5×10^−6^
16	*GRIN2A*	rs11866570	10113676	C/T	0.11	0.15	0.18	0.89 (0.12)	0.35	0.51 (0.11)	1×10^−3^	8×10^−6^	0.92 (0.11)	0.46	0.54 (0.11)	2×10^−3^	3×10^−5^
16	*GRIN2A*	rs1448253	10128367	C/T	0.09	0.13	0.67	0.83 (0.11)	0.18	0.52 (0.12)	3×10^−3^	4×10^−6^	0.84 (0.11)	0.18	0.55 (0.11)	3×10^−3^	5×10^−6^
**Well-established PD-associated genes identified via their main effect**																	
4	*SNCA*	rs356220	90860363	T/C	0.43	0.35	0.77	1.37 (0.16)	0.01	1.25 (0.23)	0.23	3×10^−5^	1.36 (0.11)	2×10^−4^	1.05 (0.14)	0.69	3×10^−6^
4	*SNCA*	rs356168	90893454	G/A	0.51	0.44	0.89	1.33 (0.17)	0.03	1.33 (0.26)	0.16	1×10^−4^	1.28 (0.10)	3×10^−3^	1.09 (0.14)	0.51	5×10^−5^
17	*MAPT*	rs199533	42184098	T/C	0.17	0.22	0.15	0.65 (0.08)	2×10^−4^	1.10 (0.21)	0.63	7×10^−5^	0.68 (0.07)	2×10^−4^	1.10 (0.18)	0.57	7×10^−5^
6	*HLA*	rs3129882	32517508	G/A	0.46	0.40	0.78	1.25 (0.15)	0.07	1.26 (0.24)	0.22	2×10^−3^	1.24 (0.10)	0.01	1.08 (0.14)	0.54	5×10^−4^

Also see Figure 1. GWAIS analysis was [SNP+SNP*coffee] test with 2 df adjusting for sex, age, PC1 and PC2. The test examines the significance of the SNP main effect and its interaction with coffee, without introducing the significant effect of coffee on PD. Results for *GRIN2A* were equally significant under Dominant and Additive models. Recessive model had no clear signal (see Figure S1). Also shown are the results obtained with the same dataset and under the same analytic model for the known PD genes *SNCA*, *MAPT* and *HLA*. *SNCA* and *HLA* had reached P<5×10^−8^ in our GWAS. The fall in significance in GWAIS is due in part to 1/3 reduction in sample size due to unavailability of coffee data, and also the penalty imposed by the added degree of freedom. *GRIN2A* did not have a strong main effect to be noticed in GWAS, but in GWAIS, the inclusion of coffee and interaction placed *GRIN2A* higher than *SNCA*, *HLA* and *MAPT*.

GWAIS results described above were obtained from a test that measures the combined significance of the SNP and its interaction with coffee on risk of PD [15]. The test has 2 df; hence when interaction is absent, GWAIS is less powerful than GWAS which has only 1 df. Furthermore, the sample size was smaller in GWAIS because it required not only genotypes but also coffee data, which was available for 2/3 of NGRC. Under these conditions, GWAIS produced P_2df_>10^−6^ (Figure 1A) for the top SNP in *SNCA* which had reached P = 3×10^−11^ in NGRC GWAS [5]. This drop in significance demonstrates the dramatic loss of power in GWAIS as compared to GWAS. Under these conditions, GWAIS yielded P_2df_ = 1×10^−6^ for rs4998386 in *GRIN2A* (as compared to P_2df_ = 3×10^−6^ for *SNCA* and P_2df_ = 7×10^−5^ for *MAPT*). Dominant and Additive models produced nearly identical results for *GRIN2A* SNPs (Table 1). Recessive model had no notable signal (Figure S1).

### GWAS in heavy and light coffee-drinkers in Discovery samples

**Figure 2:**
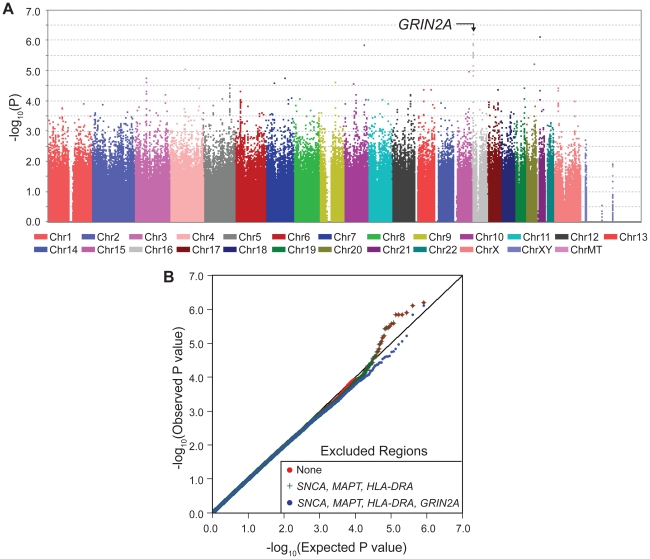
GWAS in heavy coffee-drinkers. Panel A depicts GWAS in heavy coffee drinkers with *GRIN2A* achieving the lowest P values. The P values in stratified GWAS are for genotyped SNP's main effect on PD risk, adjusted for sex, age, PC1 and PC2. Imputed SNPs (not shown here) achieved P = 3×10^−8^ (see Table 4). Additive model is shown here; see Figure S1 for Dominant model. Dominant and additive models yielded similar results for top hits (see Table 2). Panel B is the QQ plot for heavy coffee drinkers where the observed P values (red line) are plotted against the expected P values under no association (straight black line). The plots were made first by including all SNPs (red), then excluding *SNCA*, *HLA* and *MAPT* (green) and finally by excluding *GRIN2A* (blue). Unlike the QQ plot for GWAIS, the effects of *SNCA*, *HLA* and *MAPT* are unnoticeable. The only deviation is seen at the extreme <10^−5^ which is primarily due to *GRIN2A*.

**Figure 3:**
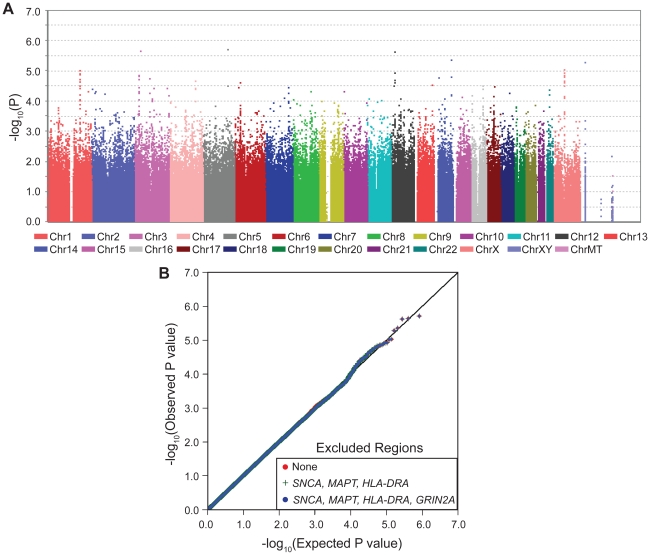
GWAS in light coffee-drinkers. Neither the Manhattan plot (Panel A) nor the QQ plot (Panel B) exhibit evidence of association between *GRIN2A* and PD among individuals who drink little or no coffee.

With one goal being pharmacogenetic applications, we were interested in genes that modulate risk in people who consume caffeine, thus we stratified the subjects as heavy drinkers or light drinkers (light includes non-drinkers) and performed GWAS in each group (SNP-PD test, 1 df). The sample size was now further reduced to only 512 cases and 387 controls who drank more than the median (heavy drinkers) and 946 cases and 544 control subjects who drank less than the median (light drinkers). As expected due to interaction, which suggests different association patterns across categories, most of the signals seen in GWAIS (Figure 1A) appeared within either heavy drinkers (Figure 2, Table 2, Figure S1) or light drinkers (Figure 3, Table 2, Figure S1). In heavy drinkers, the focus of this study, the most significant result was *GRIN2A*_rs4998386 (P = 6×10^−7^) and 11 neighboring SNPs (P = 10^−5^ to 10^−6^, Table 2).

**Table 2:** *GRIN2A* was the most significant result in GWAS in heavy coffee-drinkers.

					GWAS in Heavy Coffee Drinkers						GWAS in Light Coffee Drinkers					
					MAF	MAF	Dominant		Additive		MAF	MAF	Dominant		Additive	
CHR	GENE	SNP	BP	Minor/Major Allele	Case	Control	OR (SE)	P	OR (SE)	P	Case	Control	OR (SE)	P	OR (SE)	P
**Novel PD-association identified via interaction with coffee**																
16	*GRIN2A*	rs4998386	9978046	T/C	0.07	0.14	0.41 (0.07)	8×10^−7^	0.43 (0.07)	6×10^−7^	0.09	0.11	0.82 (0.12)	0.18	0.84 (0.11)	0.19
16	*GRIN2A*	rs17569693	9987686	G/A	0.07	0.13	0.41 (0.08)	1×10^−6^	0.44 (0.08)	3×10^−6^	0.09	0.11	0.79 (0.11)	0.10	0.84 (0.11)	0.09
16	*GRIN2A*	rs8043728	10003004	T/C	0.08	0.14	0.41 (0.08)	5×10^−6^	0.44 (0.08)	7×10^−6^	0.09	0.11	0.82 (0.12)	0.17	0.84 (0.11)	0.13
16	*GRIN2A*	rs8056683	10052710	T/C	0.08	0.14	0.43 (0.08)	2×10^−6^	0.44 (0.08)	3×10^−6^	0.10	0.12	0.82 (0.12)	0.17	0.84 (0.11)	0.14
16	*GRIN2A*	rs9927926	10057405	C/T	0.08	0.14	0.43 (0.08)	2×10^−6^	0.44 (0.08)	3×10^−6^	0.10	0.12	0.82 (0.12)	0.17	0.84 (0.11)	0.14
16	*GRIN2A*	rs17671033	10068727	A/G	0.08	0.14	0.43 (0.08)	2×10^−6^	0.44 (0.08)	3×10^−6^	0.10	0.11	0.82 (0.12)	0.25	0.85 (0.11)	0.20
16	*GRIN2A*	rs9933111	10072100	G/A	0.08	0.15	0.42 (0.08)	1×10^−6^	0.45 (0.07)	1×10^−6^	0.10	0.12	0.82 (0.12)	0.24	0.85 (0.11)	0.20
16	*GRIN2A*	rs13331465	10077968	T/C	0.08	0.15	0.42 (0.08)	1×10^−6^	0.45 (0.07)	1×10^−6^	0.10	0.12	0.82 (0.12)	0.24	0.85 (0.11)	0.20
16	*GRIN2A*	rs13336632	10078155	C/A	0.08	0.15	0.42 (0.08)	1×10^−6^	0.45 (0.07)	1×10^−6^	0.10	0.12	0.82 (0.12)	0.24	0.85 (0.11)	0.20
16	*GRIN2A*	rs1448270	10082819	T/G	0.08	0.14	0.43 (0.08)	3×10^−6^	0.47 (0.08)	3×10^−6^	0.10	0.12	0.86 (0.12)	0.29	0.85 (0.11)	0.24
16	*GRIN2A*	rs11866570	10113676	C/T	0.09	0.16	0.46 (0.08)	3×10^−6^	0.51 (0.08)	1×10^−5^	0.12	0.13	0.88 (0.12)	0.35	0.85 (0.11)	0.45
16	*GRIN2A*	rs1448253	10128367	C/T	0.08	0.15	0.44 (0.08)	2×10^−6^	0.47 (0.08)	4×10^−6^	0.10	0.12	0.83 (0.11)	0.18	0.84 (0.11)	0.18
**Well-established PD-associated genes identified via their main effect**																
4	*SNCA*	rs356220	90860363	T/C	0.42	0.34	1.71 (0.25)	2×10^−4^	1.43 (0.15)	6×10^−4^	0.43	0.36	1.37 (0.16)	0.01	1.36 (0.11)	2×10^−4^
4	*SNCA*	rs356168	90893454	G/A	0.50	0.42	1.75 (0.27)	3×10^−4^	1.38 (0.14)	1×10^−3^	0.51	0.45	1.33 (0.17)	0.03	1.28 (0.10)	3×10^−3^
17	*MAPT*	rs199533	42184098	T/C	0.16	0.21	0.71 (0.10)	0.02	0.75 (0.10)	0.02	0.17	0.23	0.65 (0.08)	3×10^−4^	0.69 (0.07)	3×10^−4^
6	*HLA*	rs3129882	32517508	G/A	0.47	0.40	1.57 (0.24)	2×10^−3^	1.34 (0.13)	4×10^−3^	0.45	0.40	1.25 (0.15)	0.07	1.24 (0.10)	0.01

See also Figure 2. Standard GWAS (PD-SNP association, no interaction) was conducted among heavy and light coffee drinkers separately. *GRIN2A* was most notable only among heavy coffee drinkers. Odds ratio (OR) of 0.41–0.46 suggests that among heavy coffee drinkers, who are known to be at reduced risk for PD, *GRIN2A* genotypes further modifies risk by over two-fold. As expected due to interaction, *GRIN2A* did not have a significant effect in light coffee drinkers. This is in contrast to known PD genes which exhibited their effects on PD risk regardless of coffee consumption.

The QQ plots for stratified GWAS also demonstrate clearly that *GRIN2A* is the single primary PD associated locus in heavy coffee drinkers (Figure 2): exclusion of *SNCA*, *HLA* and *MAPT* did not have an impact in heavy drinkers, whereas exclusion of *GRIN2A* nearly abolished the extreme P values of 10^−5^–10^−6^. No clear signals were detected in light coffee drinkers (Figure 3).

The 12 *GRIN2A* SNPs that were associated with PD via heavy coffee consumption had similar minor allele frequencies (MAF = 0.13–0.16 in controls) and odds ratios (OR = 0.43–0.51) and were in strong LD (Figure S3). Haplotype analysis did not strengthen the signal. Within this gene varying CNV software tools called either no CNVs or just two CNVs in controls. Thus, CNVs are unlikely to explain a large fraction of the phenotype variability. We therefore selected only the SNP with the lowest P value for replication (*GRIN2A*_rs4998386).

### Genotype-specific association of coffee with PD in Discovery

Testing the association of coffee with PD in NGRC, when calculated irrespective of genotype, showed an average of 34% lower PD risk in heavy coffee drinkers than in light drinkers (OR = 0.66, P = 6×10^−6^, Table 3, Coffee irrespective of genotype). *GRIN2A*, irrespective of coffee, had a modest main effect on PD in NGRC (Table 3, *GRIN2A* rs4998386 genotype irrespective of coffee). A key question was if, and to what degree, *GRIN2A*_rs4998386 genotype modifies the effect of coffee on PD risk (Table 3): Within heavy drinkers, PD risk was 58% lower (OR = 0.42, P = 2×10^−6^) for rs4998386_TC, and 81% lower (OR = 0.19, P = 0.05) for rs4998386_TT genotype than rs4998386_CC; whereas in light drinkers genotype had no effect on risk. Similar results were obtained for Additive and Dominant models (Table S3). The joint effect comparing rs4998386_TC genotype and heavy coffee vs. rs4998386_CC genotype and light coffee was most dramatic, suggesting a highly significant 68% risk reduction (OR = 0.32, P = 7×10^−11^) in NGRC (Table 3, Joint effects of *GRIN2A* rs4998386 and coffee).

**Table 3:** PD risk conditioned on *GRIN2A* genotype and coffee use.

*GRIN2A*	Coffee	NGRC (Discovery)				Pooled Replications				Pooled NGRC+Replications			
		N Case	N Control	OR (SE)	P	N Case	N Control	OR (SE)	P	N Case	N Control	OR (SE)	P
**Coffee irrespective of genotype**													
-	Light	946	544	Ref		621	1012	Ref		1567	1556	Ref	
-	Heavy	512	387	0.66 (0.06)	6×10^−6^	393	905	0.79 (0.07)	2×10^−3^	905	1292	0.73 (0.04)	3×10^−7^
***GRIN2A*** ** rs4998386 genotype irrespective of coffee**													
CC	-	1227	716	Ref		837	1558	Ref		2064	2274	Ref	
TC		219	204	0.62 (0.07)	2×10^−5^	163	344	0.89 (0.10)	0.14	382	548	0.75 (0.06)	2×10^−4^
TT		12	11	0.53 (0.23)	0.15	14	15			26	26	Heterogeneity P = 0.06*	
CC	Heavy	441	283	Ref		330	706	Ref.		771	989	Ref	
TC	Heavy	69	99	0.42 (0.08)	2×10^−6^	54	192	0.59 (0.10)	1×10^−3^	123	291	0.51 (0.06)	7×10^−8^
TT	Heavy	2	5	0.19 (0.16)	0.05	9	7			11	12	Heterogeneity P = 0.04*	
CC	Light	786	433	Ref		507	852	Ref.		1293	1285	Ref	
TC	Light	150	105	0.81 (0.12)	0.16	109	152	1.24 (0.18)	0.93	259	257	1.00 (0.10)	0.99
TT	Light	10	6	0.81 (0.44)	0.70	5	8			15	14		
**Joint effects of ** ***GRIN2A*** ** rs4998386 and coffee**													
CC	Light	786	433	Ref.		507	852	Ref.		1293	1285	Ref	
CC	Heavy	441	283	0.75 (0.08)	6×10^−3^	330	706	0.88 (0.08)	0.08	771	989	0.82 (0.06)	3×10^−3^
TC	Light	150	105	0.81 (0.12)	0.15	109	152	1.24 (0.18)	0.07	259	257	1.00 (0.10)	0.99
TC	Heavy	69	99	0.32 (0.06)	7×10^−11^	54	192	0.52 (0.09)	5×10^−5^	123	291	0.41 (0.05)	6×10^−13^
TT	Light	10	6	0.81 (0.44)	0.70	5	8			15	14		
TT	Heavy	2	5	0.14 (0.12)	0.02	9	7			11	12		
**Interaction of ** ***GRIN2A*** ** rs4998386 genotypes and coffee consumption**													
		1446	920	0.52 (0.12)	4×10^−3^	1000	1902	0.48 (0.11)	5×10^−4^	2446	2822	0.51 (0.08)	3×10^−5^
**Genotype specific dose-dependent effect of coffee**													
CC	≤25%	334	189	Ref		117	98	Ref.		451	287	Ref	
	25%–≤50%	344	178	1.03 (0.14)	0.84	120	92	1.11 (0.12)	0.70	464	270	1.06 (0.12)	0.61
	50%–≤75%	366	203	0.91 (0.12)	0.47	91	87	0.83 (0.17)	0.19	457	290	0.89 (0.10)	0.30
	>75%	183	146	0.58 (0.09)	3×10^−4^	75	82	0.68 (0.15)	0.04	258	228	0.61 (0.08)	6×10^−5^
TC	≤25%	69	55	Ref		27	18	Ref.		96	73	Ref	
	25%–≤50%	65	41	1.31 (0.37)	0.34	22	16	0.89 (0.41)	0.40	87	57	1.24 (0.30)	0.36
	50%–≤75%	59	56	0.71 (0.20)	0.21	16	22	0.40 (0.19)	0.03	75	78	0.63 (0.15)	0.05
	>75%	26	52	0.31 (0.10)	2×10^−4^	13	21	0.37 (0.18)	0.02	39	73	0.34 (0.09)	5×10^−5^
TT	≤25%	6	0			2	2			8	2		
	25%–≤50%	2	3			0	3			2	6		
	50%–≤75%	2	4			3	1			5	5		
	>75%	2	4			3	1			5	5		

C genotype was associated with reduced risk consistently across studies. rs4998386_TT frequency varied significantly across studies. The a-priori hypothesis for replication that among heavy drinkers *GRIN2A*_rs4998386_T carriers had a lower risk of PD than *GRIN2A*_rs4998386_CC was replicated under three conditions: comparing TC to CC (excluding rare and variable TT genotype) shown here, Dominant model (TT+TC vs. CC) and Additive model (TT vs. TC vs CC) shown in Table S3. As predicted from the Discovery phase, genotype had no effect on risk of PD among light coffee drinkers. The joint effects of genotype and coffee showed a significant 59% drop in PD risk in people who had the rs4998386_TC genotype and were heavy drinkers, but little or no effect in other combinations. A formal interaction test demonstrated that the effects of coffee and genotype are dependent on each other. By definition, statistical interaction exists if the joint effect of gene (g) and exposure (e) is significantly different from the product of their individual effects. Interaction OR is the ratio of the OR of disease when g and e are present, divided by the product of the individual OR; i.e., OR_interaction_ = OR_g+e_/(OR_g_×OR_e_). (F) Dose-dependent risk reduction by coffee was clear and strong for rs4998386_TC genotype. Analyses were repeated with smoking (Table S4) or caffeinated soda/tea (Table S5) as additional covariates, results were unchanged.

*Heterogeneity P: Breslow-Day test statistics to assess between-study heterogeneity conducted for coffee and genotypes and found to be significant only for TT genotype. Analyses were adjusted for sex and age at interview in each dataset, and also for study in the pooled analyses. Expanded analysis including results for individual replication data sets are shown in Table S3.

### Hypothesis for replication

We used GWAIS as a means to identify genes that might enhance the inverse association of coffee with PD with the goal of carrying the discovery forward as a genetic marker for use in pharmacogenetic studies. Hence, the replication hypothesis was specified a-priori, based on results of NGRC, as follows: “Among heavy coffee drinkers, carriers of rs4998386_T allele have lower risk of PD than carriers of rs4998386_CC genotype”. Although this test does not reflect our most significant results, it is the test that has the clearest interpretation because it keeps the effect of coffee constant. For example, comparing TC+heavy vs. CC+light gave larger effect size and the P value was 3-orders of magnitude lower than the specified hypothesis, however, unlike our hypothesis, the test included coffee, which would have made it difficult to draw firm conclusions about the effect of genotype on coffee's inverse association with PD.

### Potential confounders

Before attempting replication, the following analyses were conducted to identify potential confounders (Table S2). We tested the frequency of rs4998386 and coffee use across disease-specific strata and population structure. There was no evidence for heterogeneity by presence or absence of family history of PD, age at onset, or recruitment site. rs4998386 frequency was different between Ashkenazi-Jewish and non-Jewish individuals (P = 0.02) and across the European countries of ancestral origin (P = 3×10^−3^) in cases, but not in controls, which, PD being heterogeneous, may indicate different ethnic-specific clusters of disease subtypes as has been noted for *LRRK2*-associated PD [34]. Not surprisingly, heavy coffee use was associated with smoking (P<10^−4^), which itself is inversely associated with PD risk independently of coffee [12]. Adjusting for smoking, in addition to other covariates, did not change the results (Table S4). We also repeated the analyses adjusting for caffeinated soda and caffeinated tea consumption and found the results to be robust (Table S5). Some reports suggest persons with PD are more likely to avoid sensation seeking and addictive behaviors [35] and *GRIN2A* polymorphisms have been implicated in predisposition to heroin addiction [36] and smoking [37] raising the concern that our results could have been confounded if the *GRIN2A* SNPs identified here were associated with habitual coffee drinking. However, there was no evidence for association between any of the *GRIN2A* SNPs and heavy vs. light coffee consumption in cases and controls combined (OR = 0.95–1.01, P = 0.61–0.94).

### Replication

See Table 3, Table S3. The a-priori hypothesis for replication that among heavy drinkers *GRIN2A*_rs4998386_T carriers had a lower risk of PD than *GRIN2A*_rs4998386_CC was replicated comparing TC to CC (excluding rare heterogeneous TT genotype): OR = 0.59, P = 10^−3^; under Additive model (TT vs. TC vs. CC): OR = 0.77, P = 0.04; and Dominant model (TT+TC vs. CC): OR = 0.70, P = 0.01. Note that the Additive and Dominant models included the TT genotype which is rare and its frequency varied significantly across datasets (Table 3, Table S6). The TC vs. CC comparison is more robust for this reason; Additive and Dominant model are shown for completeness. As seen in NGRC data, genotype had no effect on risk of PD among light coffee drinkers in Replication or combined data (OR = 1.0, P = 0.99).

In pooled Replication (without Discovery), the [SNP+SNP*coffee] joint test yielded P_2df_ = 2.3×10^−3^ comparing TC to CC (excluding rare heterogeneous TT genotype); P_2df_ = 0.12 for the Additive model, P_2df_ = 0.02 for the Dominant model. The pooled analysis of Replication and Discovery with the [SNP+SNP*coffee] joint test yielded, P_2df_ = 1.9×10^−7^ comparing TC to CC (excluding rare heterogeneous TT genotype), P_2df_ = 1.4×10^−5^ for the Additive model, and P_2df_ = 8.6×10^−7^ for the Dominant model.

In pooled data, compared to the light coffee drinkers with *GRIN2A*_rs4998386_CC genotype (the group with highest risk), heavy coffee use (with CC genotype) reduced risk by 18% (OR = 0.82, P = 3×10^−3^), having *GRIN2A*_rs4998386_T allele (light coffee) had no effect on risk (OR = 1.0, P = 0.99), but the combination of heavy coffee use and *GRIN2A*_rs4998386_TC genotype was associated with a highly significant 59% risk reduction (OR = 0.41, P = 6×10^−13^) (Table 3, Joint effects of *GRIN2A* rs4998386 and coffee).

### Imputation

See Table 4, Table S7. The array used in the study, Illumina OMNI-1 had nearly a million SNPs, which is a relatively dense coverage, but which could be further improved by imputing the SNPs that were not on the array using 1000 Genomes and HapMap data, a practice that has successfully aided many projects. After QC, we had over 3.5 million imputed and genotyped SNPs per individual in NGRC, each with information score ≥0.95 (measure of imputation certainty), and each passing standard GWAS QC. Imputation could only be applied to NGRC (Discovery) because only NGRC had genome-wide data. GWAIS and GWAS analysis of the *GRIN2A* region with imputed SNPs uncovered a block of densely linked SNPs embedded amongst the genotyped *GRIN2A*, six of which achieved P_2df_≤5×10^−8^ in GWAIS (Table 4). The interaction term was OR = 0.44, P = 4×10^−5^ (Table 4). In GWAS conducted in heavy coffee drinkers, 12 SNPs achieved P = 3×10^−8^ to 5×10^−8^ with OR = 0.41–0.42 (Table 4).

**Table 4:** GWAIS and GWAS results on combined genotyped and imputed data.

SNP	BP			GWAIS in all NGRC subjects							GWAS In NGRC heavy coffee drinkers			
				MAF	MAF	SNP		Interaction		2DF	MAF	MAF	OR	P
		Minor/Major Allele	Impute Info Score	Case	Control	OR (SE)	P	OR (SE)	P	P	Case	Control	(SE)	
16-10105921	10105921	T/C	0.98	0.11	0.15	0.91 (0.11)	0.45	0.44 (0.09)	4×10^−5^	5×10^−8^	0.09	0.17	0.41 (0.07)	3×10^−8^
16-10103787	10103787	G/A	0.98	0.11	0.15	0.91 (0.11)	0.45	0.44 (0.09)	4×10^−5^	5×10^−8^	0.09	0.17	0.41 (0.07)	3×10^−8^
16-10102229	10102229	T/C	0.98	0.11	0.15	0.91 (0.11)	0.45	0.44 (0.09)	4×10^−5^	5×10^−8^	0.09	0.17	0.41 (0.07)	3×10^−8^
16-10102124	10102124	T/C	0.98	0.11	0.15	0.91 (0.11)	0.45	0.44 (0.09)	4×10^−5^	5×10^−8^	0.09	0.17	0.41 (0.07)	3×10^−8^
rs56275045	10108893	A/C	0.99	0.11	0.15	0.91 (0.11)	0.41	0.45 (0.09)	5×10^−5^	5×10^−8^	0.09	0.17	0.42 (0.07)	3×10^−8^
16-10109203	10109203	A/T	0.99	0.11	0.15	0.91 (0.11)	0.41	0.45 (0.09)	5×10^−5^	5×10^−8^	0.09	0.17	0.42 (0.07)	4×10^−8^
16-10110896	10110896	C/T	0.98	0.11	0.15	0.91 (0.11)	0.41	0.45 (0.09)	6×10^−5^	6×10^−8^	0.09	0.17	0.42 (0.07)	4×10^−8^
16-10101465	10101465	A/G	0.98	0.11	0.15	0.91 (0.11)	0.42	0.45 (0.09)	6×10^−5^	7×10^−8^	0.09	0.17	0.41 (0.07)	5×10^−8^
16-10092692	10092692	T/C	0.98	0.11	0.15	0.91 (0.11)	0.40	0.46 (0.09)	7×10^−5^	8×10^−8^	0.09	0.17	0.42 (0.07)	5×10^−8^
16-10093997	10093997	T/C	0.98	0.11	0.15	0.91 (0.11)	0.39	0.46 (0.09)	7×10^−5^	8×10^−8^	0.09	0.17	0.42 (0.07)	5×10^−8^
16-10094528	10094528	G/A	0.98	0.11	0.15	0.91 (0.11)	0.39	0.46 (0.09)	7×10^−5^	8×10^−8^	0.09	0.17	0.42 (0.07)	5×10^−8^
rs17671178	10094708	G/A	0.98	0.11	0.15	0.90 (0.11)	0.39	0.46 (0.09)	7×10^−5^	8×10^−8^	0.09	0.17	0.42 (0.07)	5×10^−8^

*GRIN2A* was the most significant area in both the GWAIS and the GWAS in heavy coffee users. This table shows results that achieved P≤5×10^−8^; for a complete list of all SNPs that achieved P<10^−5^ see Table S7.

## Discussion

In a genome-wide gene-environment study we identified *GRIN2A* as a genetic modifier of the inverse association of coffee with the risk of developing PD. The discovery was made in NGRC, and replicated in independent data. Risk reduction by heavy coffee use, which was estimated to be 27% on average, was genotype-specific and varied according to *GRIN2A* genotype from 18% (P = 3×10^−3^) for individuals with rs4998386_CC genotype to 59% (P = 6×10^−13^) for those with rs4998386_TC genotype. When coffee intake was categorized in four doses, the dose trend was more prominent in individuals with rs4998386_T allele than those with rs4998386_CC genotype, with the 3^rd^ and 4^th^ quartiles exhibiting only 11% and 39% risk reduction for rs4998386_CC carriers, vs. 37% and 66% for rs4998386_T carriers. With imputation we uncovered a block of *GRIN2A* SNPs not included on the genotyping array, which achieved P = 3×10^−8^ to 5×10^−8^. We propose *GRIN2A* as a new modifier gene for PD, and posit that if coffee-consumption is considered, *GRIN2A* may prove to be one of the most important PD-associated genes to have emerged from genome-wide studies. We base this suggestion on statistics, biology and the potential for immediate translation to clinical medicine, as we discuss below.

*GRIN2A* had not previously been tested as a candidate gene for PD, and was not detected in PD GWAS which have all been examining gene main effects without considering interactions with relevant environmental exposures. The most significant and consistently replicated main effects detected to date are for *SNCA*, *MAPT* and *HLA*. Here we added, for the first time, a common and relevant environmental exposure (coffee) to a genome-wide study. Inclusion of coffee allowed *GRIN2A* to rise to the top. In the gene-environment (GWAIS) model, *GRIN2A* surpassed *SNCA*, *MAPT* and *HLA* in statistical significance. Among heavy coffee drinkers, the impact of *GRIN2A* on PD risk (measured as OR) was 50% greater, and 2 to 5 orders of magnitude more significant (measured as P value) than the strongest associations reported for *SNCA*, *MAPT* or *HLA*. This study is proof of concept that inclusion of environmental factors can help identify disease-associated genes that are missed in SNP-only GWAS.

*GRIN2A* is an important gene for the central nervous system. Accelerated evolution of *GRIN2A* in primates is said to have contributed to the dramatic increase in the size and complexity of the human brain which defines human evolution [38]. *GRIN2A* encodes a subunit of the N-methyl-D-aspartate-2A (NMDA) glutamate receptor. It is central to excitatory neurotransmission and the control of movement and behavior [39]–[41]. The literature suggest imbalances in NMDA-dependent neurotransmission contribute to neurodegeneration in PD, possibly through massive influx of calcium and impaired mitochondrial function leading to apoptosis; and/or disruption of glutamate-mediated autophagy which is implicated in degradation and removal of proteins like α-synuclein (see [42] for review). The portion of intron 3 containing SNPs with the most significant associations (from base pair 9978046 to base pair 10128367, Table 1, Table 2, and Table 4) includes numerous transcription factor binding sites and two peaks of enhanced histone H3K4 mono-methylation (http://genome.ucsc.edu) [43]. Polymorphisms throughout this region could therefore disrupt regulatory elements, potentially leading to variation in levels of *GRIN2A* transcript. *GRIN2A* is expressed at high levels in the brain, most notably in the subthalamic nucleus (STN) [44]. Pharmacologic inhibition of STN with an NMDA-antagonist reduces nigral neuron loss in a rodent model of PD [45]. Deep-brain-stimulation, which also targets STN, is an effective surgical symptomatic therapy for PD.

The other piece of this finding is coffee/caffeine. Our study was not designed to distinguish the active ingredients in coffee. However, we note that the largest replication study (PAGE) measured specifically the caffeine intake in mg from all food sources (drink, food, and chocolate) and replicated our hypothesis and interaction robustly. We also found trends for inverse association of tea and soda with PD, and interestingly, the varied effect size and strength of association was consistent with the relative amount of caffeine in each drink (Table S5). Thus, our data are consistent with experimental observations that caffeine is neuroprotective. Caffeine is an adenosine A_2A_-receptor antagonist. A_2A_-receptor enhances calcium influx via NMDA [41] and A_2A_-receptor antagonists are neuroprotective in animal models of PD; they attenuate excitotoxicity by reducing extracellular glutamate levels in the striatum [46], [47]. Thus interaction between coffee/caffeine and *GRIN2A* is biologically plausible, and can help formulate testable hypotheses towards a better understanding of the disease pathogenesis.

*GRIN2A* genotyping may be useful for pharmacogenetic studies. Genetics has not yet entered drug development for PD but the time is here. We now have several susceptibility loci (*SNCA*, *MAPT*, *HLA*, *BST1*, *PARK16*, *GAK* [5]–[10]) that can help identify individuals who are at moderately increased risk of developing PD. We also have at least one neuroprotective compound (coffee/caffeine) which can be pharmacologically modified to alleviate its undesirable side effects. *GRIN2A* genotyping might also inform treatments for people who already have PD. L-DOPA, the primary PD drug for 40 years, does not slow disease progression and has serious side effects. Clinical trials for new PD drugs have not found drugs that surpass the symptomatic benefits of L-DOPA. There have been numerous drug trials for glutamate-receptor blockers as well as for selective A_2A_-receptor antagonists. Most were shown to be safe, well tolerated and beneficial [17], [18], [48]; however, the majority did not reach the regulatory threshold for efficacy to be approved as PD drugs. We wonder if some of these clinical trials will succeed if patients are subdivided by *GRIN2A* genotype. We acknowledge the distinction that the present study examined risk of developing PD; whereas clinical trials have thus far aimed for symptomatic improvements in patients. Nonetheless, there are sufficient parallels to suggest that *GRIN2A* genotype might also influence efficacy of glutamate-receptor antagonists and A_2A_-receptor antagonists. This is a simple and inexpensive hypothesis that can be tested in future, ongoing and even closed clinical trials that have banked DNA.

Common non-coding variants in *GRIN2A* have been associated with Huntington disease (HD) [49], [50] and schizophrenia [51], and rare mutations have been described in patients with neurodevelopmental phenotypes [52]. Schizophrenia is associated with a (GT)n repeat in the *GRIN2A* promoter that may increase disease risk by suppressing gene expression [51]. Three *GRIN2A* SNPs have been associated with onset-age of HD; they are conserved and reportedly tag a binding site for CCAAT/enhancer-binding protein [49], [50]. HD and PD are both neurodegenerative movement disorders, thus the possibility of a common genetic element was of interest. The reported HD-associated *GRIN2A* SNPs, rs1969060, rs8057394 and rs2650427, were not on the genotyping array but were imputed with high fidelity (information score >0.99). They map within the 150 kb region identified here for PD, they are in strong LD with PD-associated SNPs defined by D' (0.48–1.0) but not by r^2^ (0–0.33) (Figure S4). We tested the HD SNPs for association with onset age and risk of PD in NGRC while conditioning on the neighboring top PD SNP (rs4998386). One HD SNP, rs8057394, yielded OR = 0.85, P = 0.02 for PD overall; OR = 0.79, P = 0.04 for heavy coffee drinkers; and OR = 0.90, P = 0.24 for light coffee drinkers. We found no other evidence for association of HD SNPs with PD, including when we jointly tested HD SNPs and possible interaction with coffee [SNP+SNP*coffee] on risk or onset of PD. Conversely, we retested, in NGRC, the association of top genotyped PD SNP (rs4998386) with PD, conditioning on HD SNP (rs8057394) and found it to be robust (P_2df_ = 8×10^−6^).

Unlike GWAS, which is now a fully standardized practice, there is no established protocol for testing gene*environment interaction on a whole-genome scale. Our strategy of starting with the joint test (GWAIS) and following up with GWAS in subgroups stratified by exposure was driven by the aims of our study. In Table S7 we present a side-by-side comparison of the results for the top *GRIN2A* SNPs (P<10^−5^), when analyzed for main effect (GWAS), for interaction, with Kraft's joint test, and in GWAS stratified by exposure. Amassing a large enough sample size for GWAIS is challenging. GWAIS requires larger sample sizes than GWAS yet there exist fewer samples that have data on relevant environmental exposures in addition to DNA and phenotype. To our knowledge, NGRC is the largest genetic study of PD that has collected exposure data. No other publically available PD GWAS has coffee data, eliminating the possibility of in-silico replication. We were able to identify and get access to only 3 datasets that had DNA and coffee, giving us a total sample size of 393 cases and 905 controls to replicate the *GRIN2A* effect in heavy coffee drinkers. In contrast, replications and meta-analyses for gene-only GWAS now have over 17,000 PD cases and controls [10]. We detected the known and confirmed PD-associated genes (*SNCA, MAPT and HLA*) in GWAIS but at much lower significance levels than in GWAS because of the smaller sample size with coffee data and the added degree of freedom in GWAIS. It is noteworthy, however, that at P_2df_ = 10^−6^, *GRIN2A* surpassed all known PD loci in significance. With the aid of imputation, we achieved P = 3×10^−8^ for a 2.4-fold difference in genotype specific effect of coffee on risk of PD. Importantly, we were able to replicate the hypothesis that we set out a-priori based on discovery.

## Supporting Information

Figure S1 GWAIS and stratified GWAS for Dominant and Recessive Models. Panel A is the Manhattan Plot of GWAIS for the Dominant model, and Panel B is for the Recessive model. Additive model is shown in Figure 1. We tested 811,597 SNPs in combination with coffee consumption for association with PD. The model was [SNP+SNP*coffee] test with 2 df, adjusted for sex, age, PC1 and PC2. Dominant and Additive models yielded similar results for the top hits (see Table 1 of main text). Panel C shows the GWAS in heavy coffee drinkers and Panel D is GWAS in light coffee drinkers, both for the Dominant model. Additive model is shown in Figure 2 (heavy drinkers) and Figure 3 (light drinkers). The P values in stratified GWAS (Panels C and D) are for SNP main effect on PD risk, adjusted for sex, age, PC1 and PC2. Dominant and Additive models yielded similar results for top hits (see Table 2 in main text). Genotyped SNPs only (imputed SNPs not included).
(PDF)

Figure S2 Map of *GRIN2A*. Panel A: Chromosomal location and gene structure of *GRIN2A*. Numbers 1–14 denote exons. Panels B and C: LD map of all the genotyped SNPs that are located in the *GRIN2A* gene or within 50 kb upstream or downstream of the gene. LD is measured as r^2^ (shades of grey) in Panel B and as D' (shades of red) in Panel C. The intensity of the color depicts strength of LD and the numbers in the grids are the values of r^2^ and D' in percentage.
(PDF)

Figure S3 LD among the PD-associated SNPs. SNPs marked in red boxes were genotyped and achieved P<10^−5^ in either 2 df GWAIS or GWAS in heavy coffee drinkers. SNPs not in red boxes were imputed and achieved P≤5×10^−8^ in either 2 df GWAIS or GWAS in heavy coffee drinkers. Panel A is r2, Panel B is D'.
(PDF)

Figure S4 LD among the PD-associated and HD-associated SNPs. SNPs marked in red boxes were genotyped and achieved P<10^−5^ in either 2 df GWAIS or GWAS in heavy coffee drinkers with PD. SNPs not in boxes were imputed and achieved P≤5×10^−8^ in either 2 df GWAIS or GWAS in heavy coffee drinkers. SNPs in blue boxes are reported as being associated with HD [50], [51]. Panel A is r2, Panel B is D'.
(PDF)

Table S1 Summary statistics on subject characteristics. NA: not available * Non-smoker: <100 cigarettes in lifetime. Smoker: ≥100 cigarettes.
(DOC)

Table S2 Frequency of *GRIN2A* rs4998386_T and heavy coffee use in NGRC by disease strata and population structure. Family history: Patients who had at least one first or second degree relative with PD were classified as familial. All others were classified as non-familial (sporadic) Age at onset: The higher coffee consumption in late-onset PD (>50 years) is because they are older than patients who have early-onset PD and therefore have had higher cumulative lifetime coffee use over the years. Smoking: having smoked ≥100 cigarettes in the lifetime qualified as smoker (a standard criterion the literature). Coffee: Number of cups of caffeinated coffee drank per day multiplied by the number of years of consumption (ccy); heavy and light divided at the median in controls (67.5 ccy). Jewish/Non-Jewish clusters: The core of the Jewish cluster was defined within 0.04≤PC1≤0.055 and 0.001≤PC2≤0.013. A core within non-Jewish Caucasian cluster was defined within −0.0075≤PC1≤0.0025 & −0.005≤PC2≤0.003. See Hamza et al. [5]. Recruitment site: US states where subjects were recruited from. Paternal & maternal ancestry: Subjects whose both paternal and maternal ancestors came from the same country. Paternal or maternal ancestry: Since having only one lineage tracing back to a country was sufficient for this classification, an individual may fall in more than one group. * Adjusted for age.
(DOC)

Table S3 PD risk conditioned on *GRIN2A* genotype and coffee use (Expanded version of Table 3). (A) Heavy coffee use was associated with 27% risk reduction (1-OR) in the pooled data. (B) *GRIN2A* rs4998386_TC genotype was associated with reduced risk consistently across studies. rs4998386_TT frequency varied significantly across studies. (C) The a-priori hypothesis for replication that among heavy drinkers *GRIN2A*_rs4998386_T carriers had a lower risk of PD than *GRIN2A*_rs4998386_CC was replicated under three conditions: comparing TC to CC (excluding rare and variable TT genotype), Dominant model (TT+TC vs. CC) and Additive model (TT vs. TC vs CC). As predicted from Discovery phase, genotype had no effect on risk of PD among light coffee drinkers. (D) The joint effects of genotype and coffee showed a significant 59% drop in PD risk in people who had the rs4998386_TC genotype and were heavy drinkers, but little or no effect in other combinations. (E) A formal interaction test demonstrated that effects of coffee and genotype are dependent on each other. By definition, statistical interaction exists if the joint effect of gene (G) and exposure (E) is significantly different from the product of their individual effects. Interaction OR is the ratio of the OR of disease when g and e are present, divided by the product of the individual OR; i.e., OR_interaction_ = OR_g+e_/(OR_g_×OR_e_). (F) Dose-dependent risk reduction by coffee was clear and strong for rs4998386_TC genotype. Analyses were repeated with smoking added as covariate, results were unchanged (Table S4). OR: odds ratio. P: statistical significance, two sided for NGRC and pooled analysis, one-sided for replication studies. *Heterogeneity P: Breslow-Day test statistics to assess between-study heterogeneity conducted for coffee and genotypes and found to be significant only for TT genotype. Analyses were adjusted for sex and age at interview in each dataset, and also for study in the pooled analyses.
(DOC)

Table S4 Smoking does not alter the results. GWAIS adjusted for smoking, sex, age, PC1, PC2 for [SNP+SNP*coffee] model gave P_2df_ = 2×10^−6^, P_interaction_ = 10^−3^. GWAS in heavy coffee-drinkers yielded OR = 0.44, P = 10^−6^. PD risk conditioned on *GRIN2A*_rs4998386 genotype and coffee use, adjusted for smoking as well as sex and age are given in Table S4.
(DOC)

Table S5 Caffeinated soda and tea do not alter the results. GWAIS and stratified GWAS results were robust when caffeinated tea and soda were included as additional covariates, along with sex, age, PC1 and PC2.
(DOC)

Table S6 Exploring characteristics of individuals with the rare TT genotype in search of the source of heterogeneity. There was no trend for any of the PD-relevant characteristics that would explain the heterogeneity in TT genotype across studies. Due to the low frequency of TT and the small number of subjects of Jewish heritage, the N = 0 for the Jewish subgroup is expected.
(DOC)

Table S7 Side-by-side comparison of results from GWAS, Interaction, GWAIS and stratified GWAS analyses for the top *GRIN2A* SNPs. Genotyped and imputed SNPs (info score ≥95%) that reached P<10^−5^ in GWAIS are shown in the order of base pair position (BP).
(DOC)
