# Supplementary figures and images for: Genome-Wide Gene-Environment Study Identifies Glutamate Receptor Gene GRIN2A as a Parkinson's Disease Modifier Gene via Interaction with Coffee
Source: PLoS Genet. 2011 Aug 18;7(8):e1002237. doi: 10.1371/journal.pgen.1002237 (PMC3158052; doi:10.1371/journal.pgen.1002237)

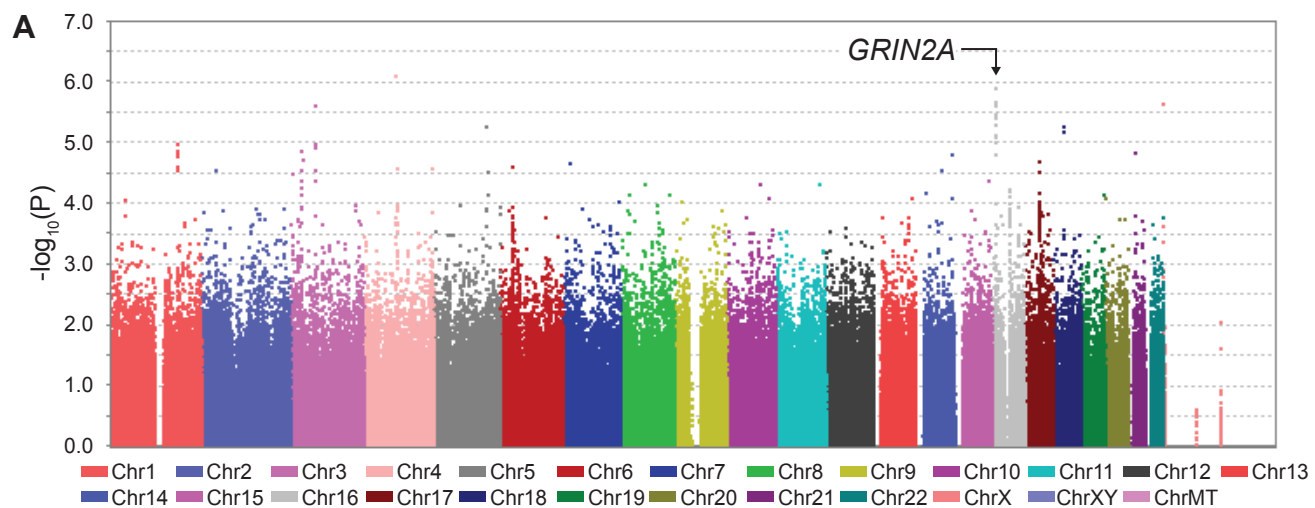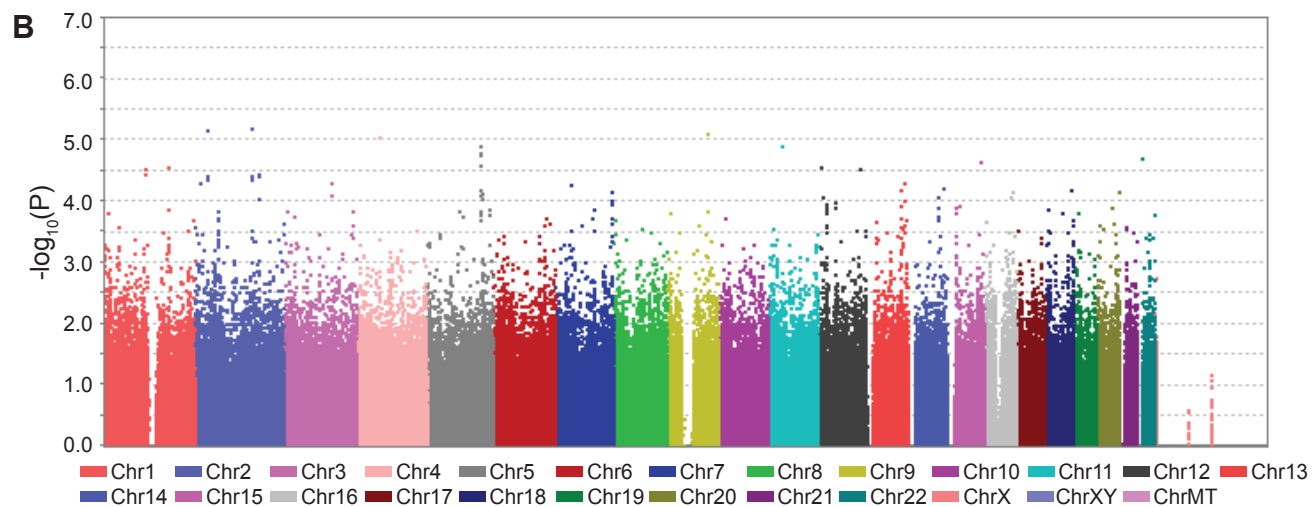

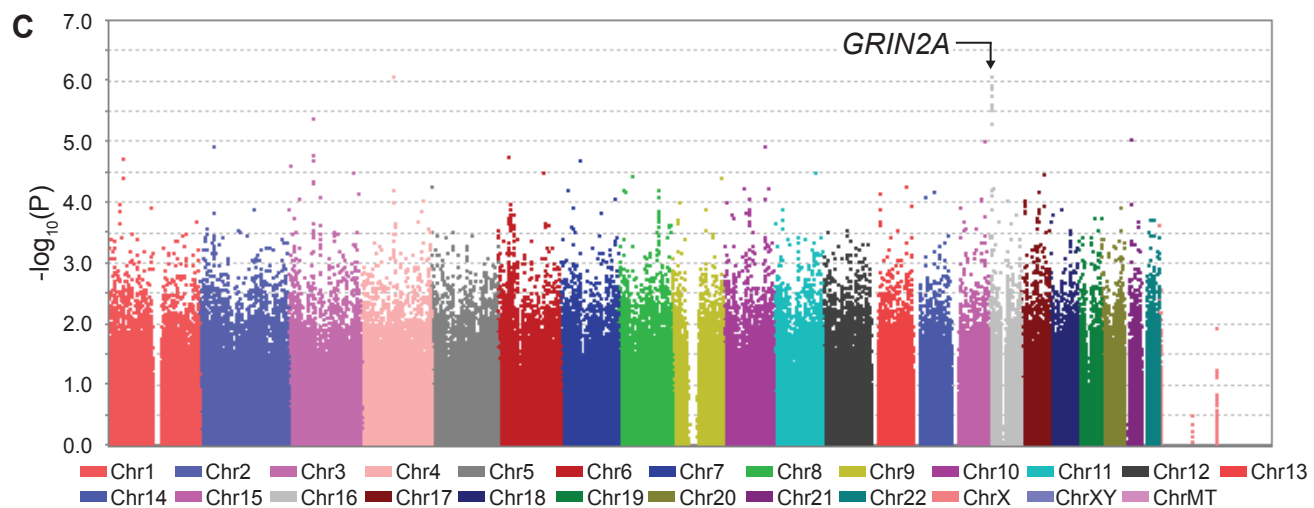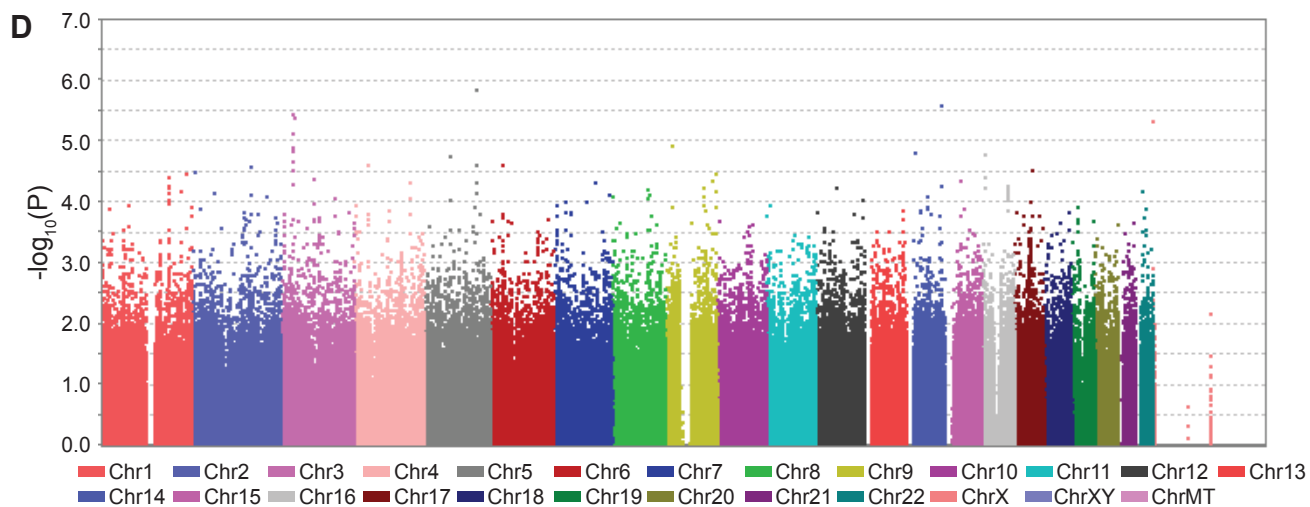

Supplement: Figure S1 — GWAIS and stratified GWAS for Dominant and Recessive Models. Panel A is the Manhattan Plot of GWAIS for the Dominant model, and Panel B is for the Recessive model. Additive model is shown in Figure 1. We tested 811,597 SNPs in combination with coffee consumption for association with PD. The model was [SNP+SNP*coffee] test with 2 df, adjusted for sex, age, PC1 and PC2. Dominant and Additive models yielded similar results for the top hits (see Table 1 of main text). Panel C shows the GWAS in heavy coffee drinkers and Panel D is GWAS in light coffee drinkers, both for the Dominant model. Additive model is shown in Figure 2 (heavy drinkers) and Figure 3 (light drinkers). The P values in stratified GWAS (Panels C and D) are for SNP main effect on PD risk, adjusted for sex, age, PC1 and PC2. Dominant and Additive models yielded similar results for top hits (see Table 2 in main text). Genotyped SNPs only (imputed SNPs not included). (PDF) [file pgen.1002237.s001.pdf]

**A** | chr16 (p13.2) | p13.3 13.2 | 12.3 | p12.1 16p11.2 | q11.2 q12.1 | 13 16q21 22.1 | q23.1

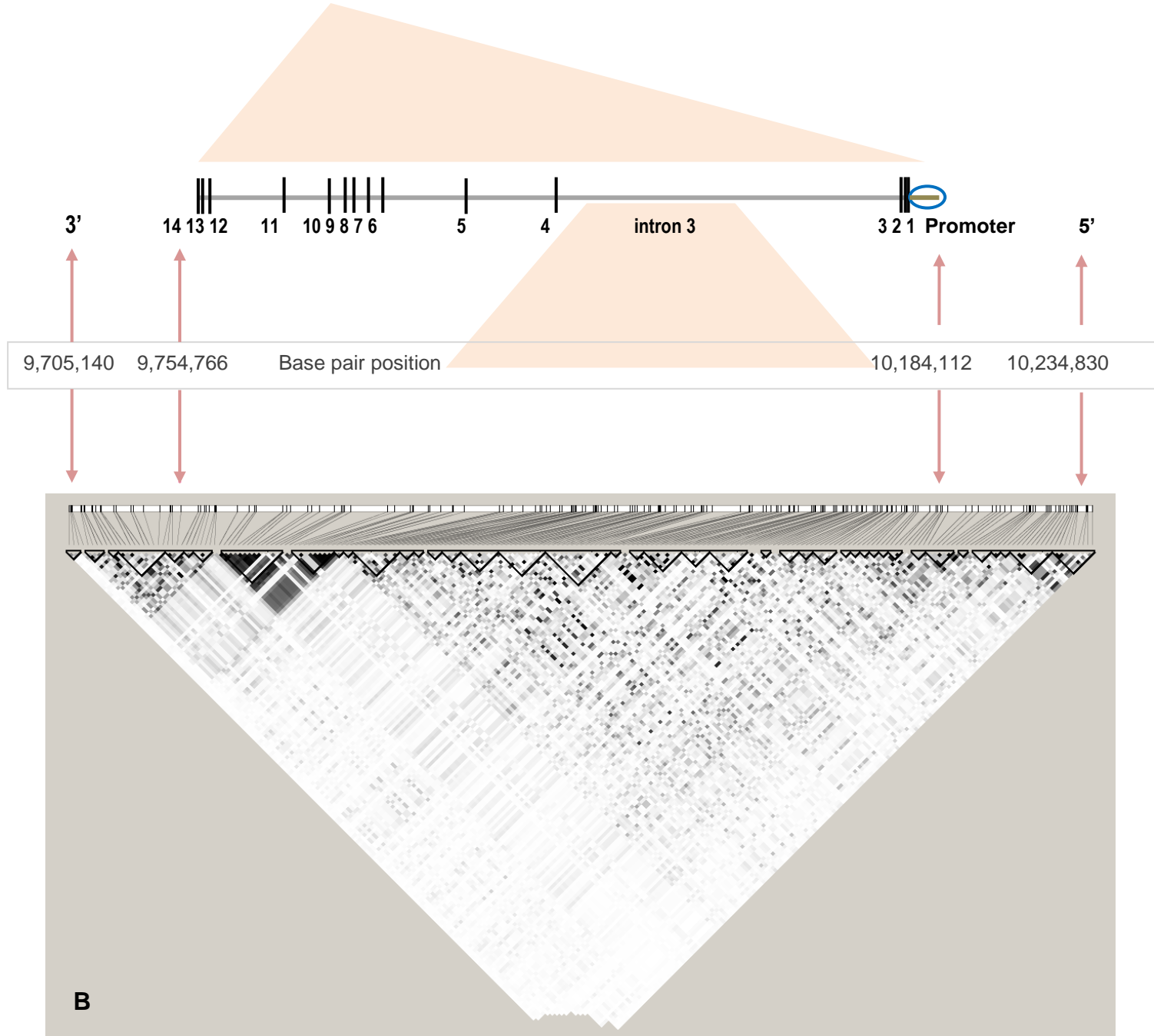

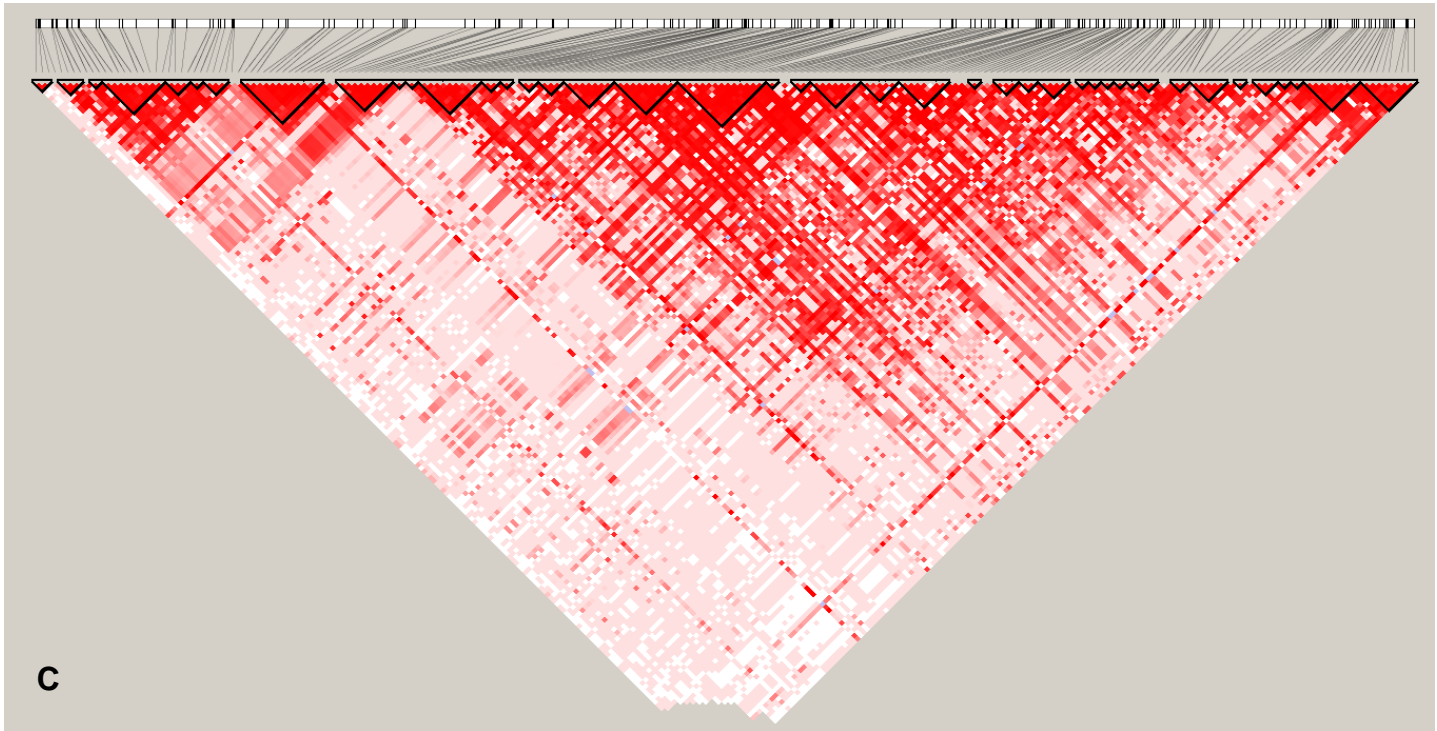

Supplement: Figure S2 — Map of GRIN2A. Panel A: Chromosomal location and gene structure of GRIN2A. Numbers 1–14 denote exons. Panels B and C: LD map of all the genotyped SNPs that are located in the GRIN2A gene or within 50 kb upstream or downstream of the gene. LD is measured as r2 (shades of grey) in Panel B and as D' (shades of red) in Panel C. The intensity of the color depicts strength of LD and the numbers in the grids are the values of r2 and D' in percentage. (PDF) [file pgen.1002237.s002.pdf]

A

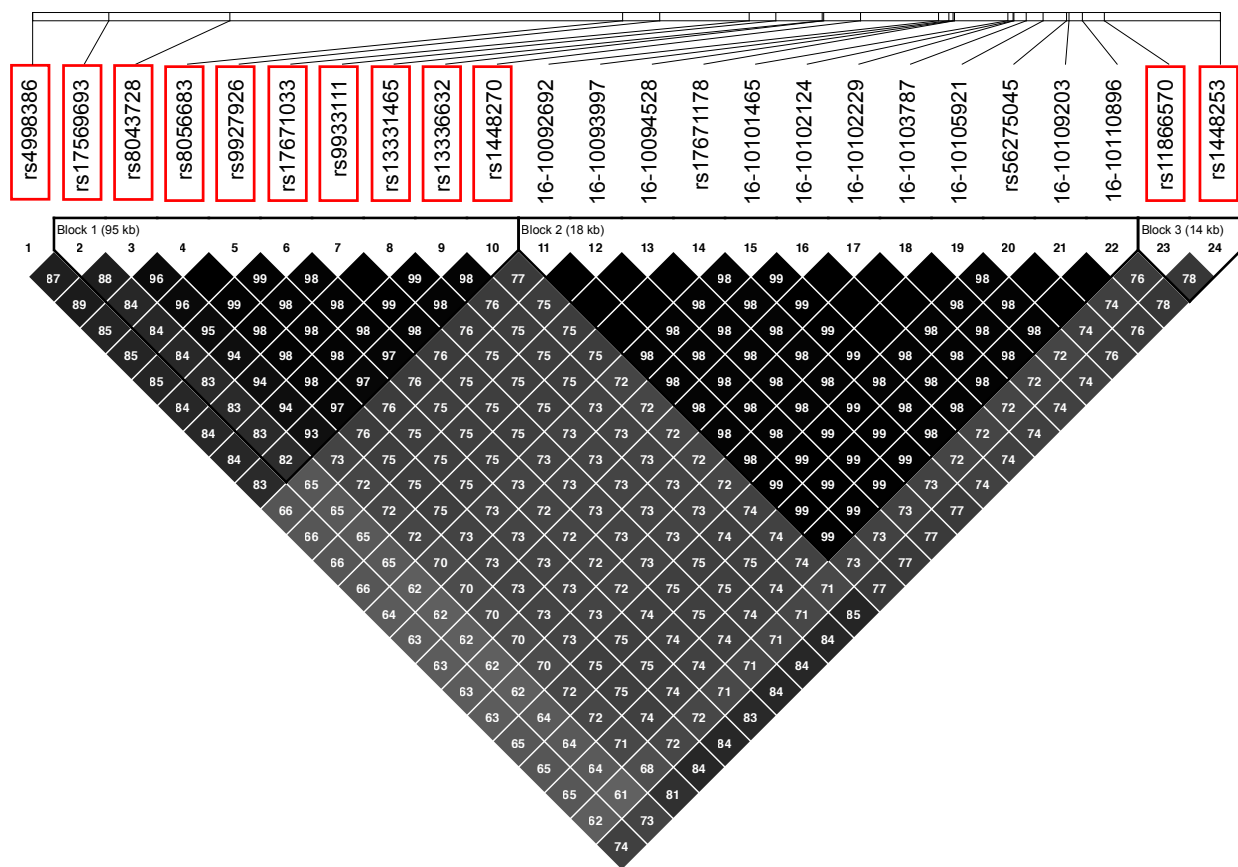

B

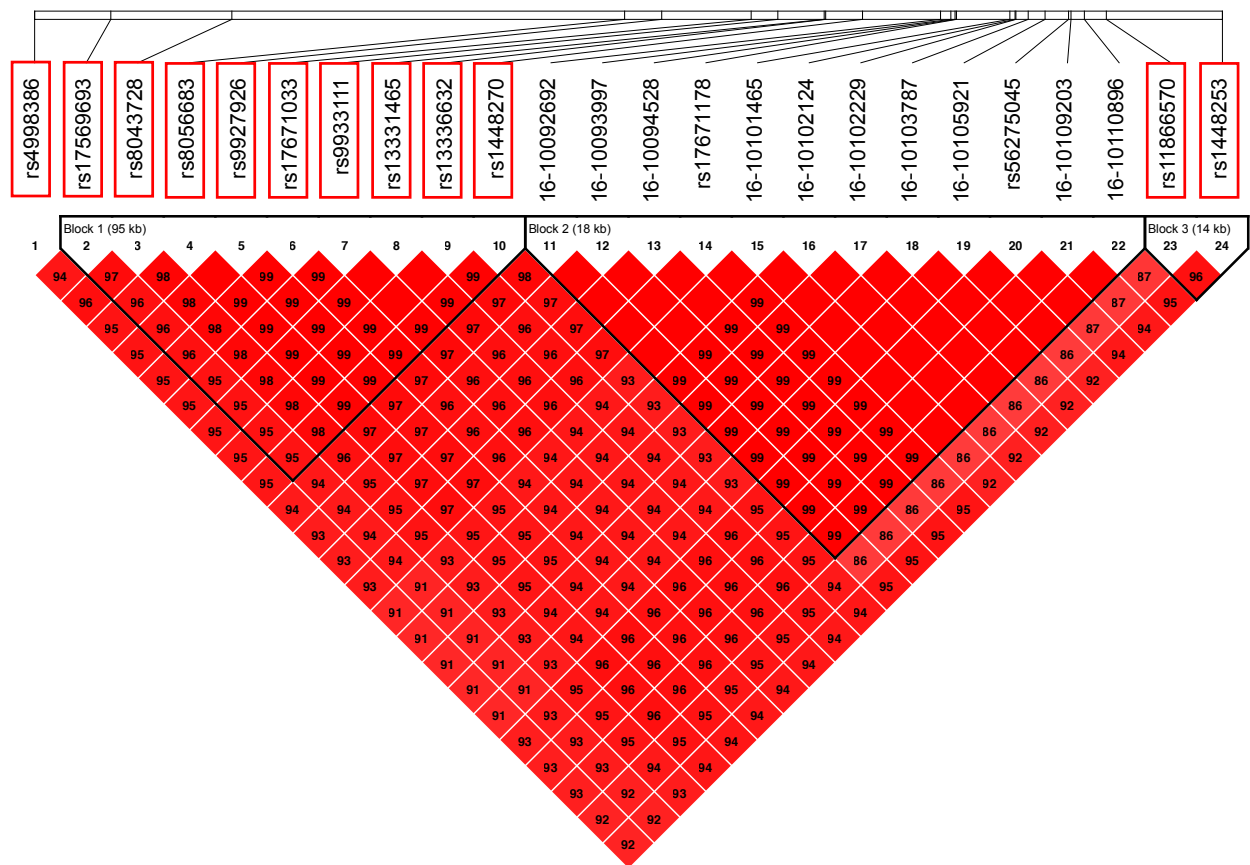

Supplement: Figure S3 — LD among the PD-associated SNPs. SNPs marked in red boxes were genotyped and achieved P<10−5 in either 2 df GWAIS or GWAS in heavy coffee drinkers. SNPs not in red boxes were imputed and achieved P≤5×10^−8^ in either 2 df GWAIS or GWAS in heavy coffee drinkers. Panel A is r2, Panel B is D'. (PDF) [file pgen.1002237.s003.pdf]

A

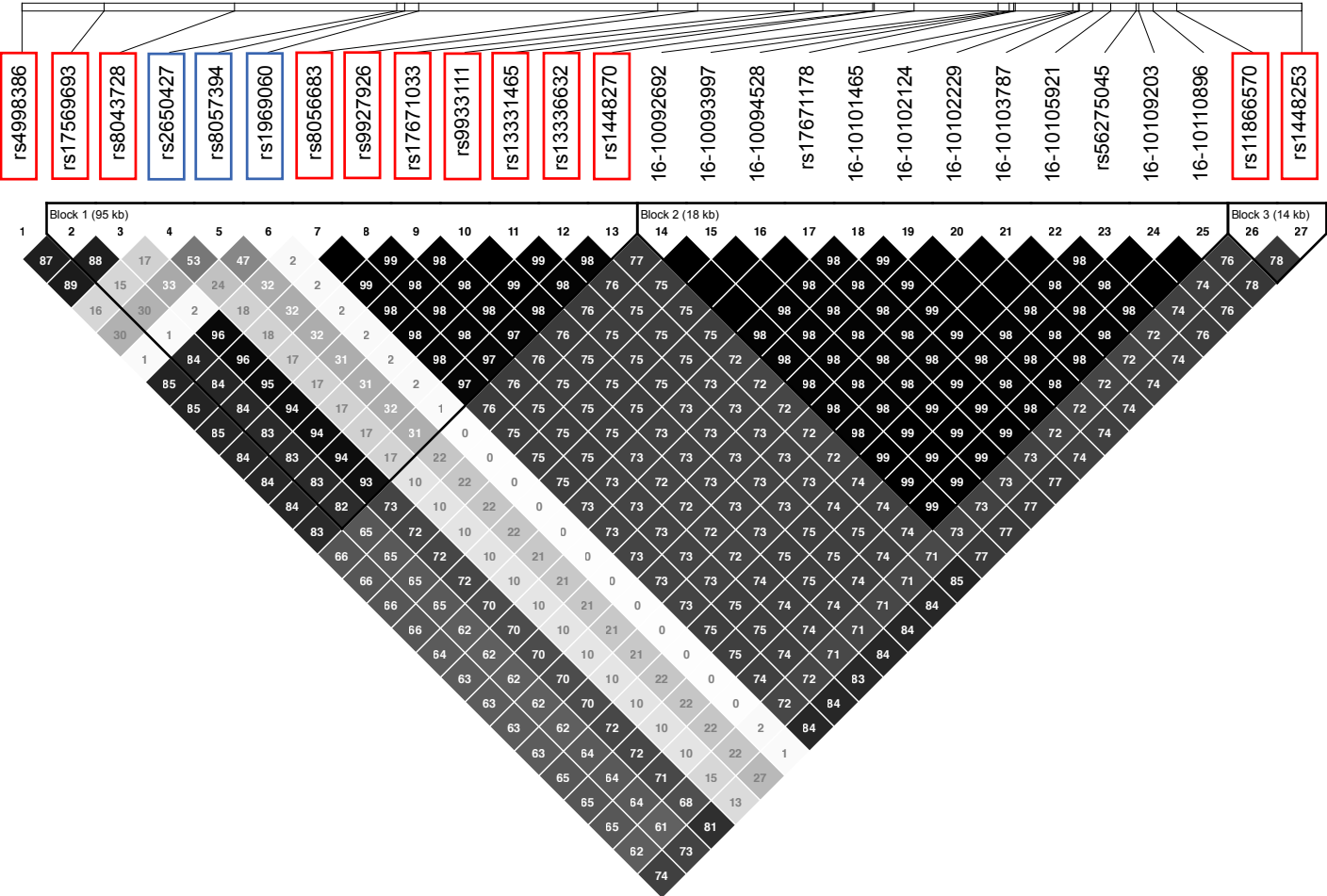

B

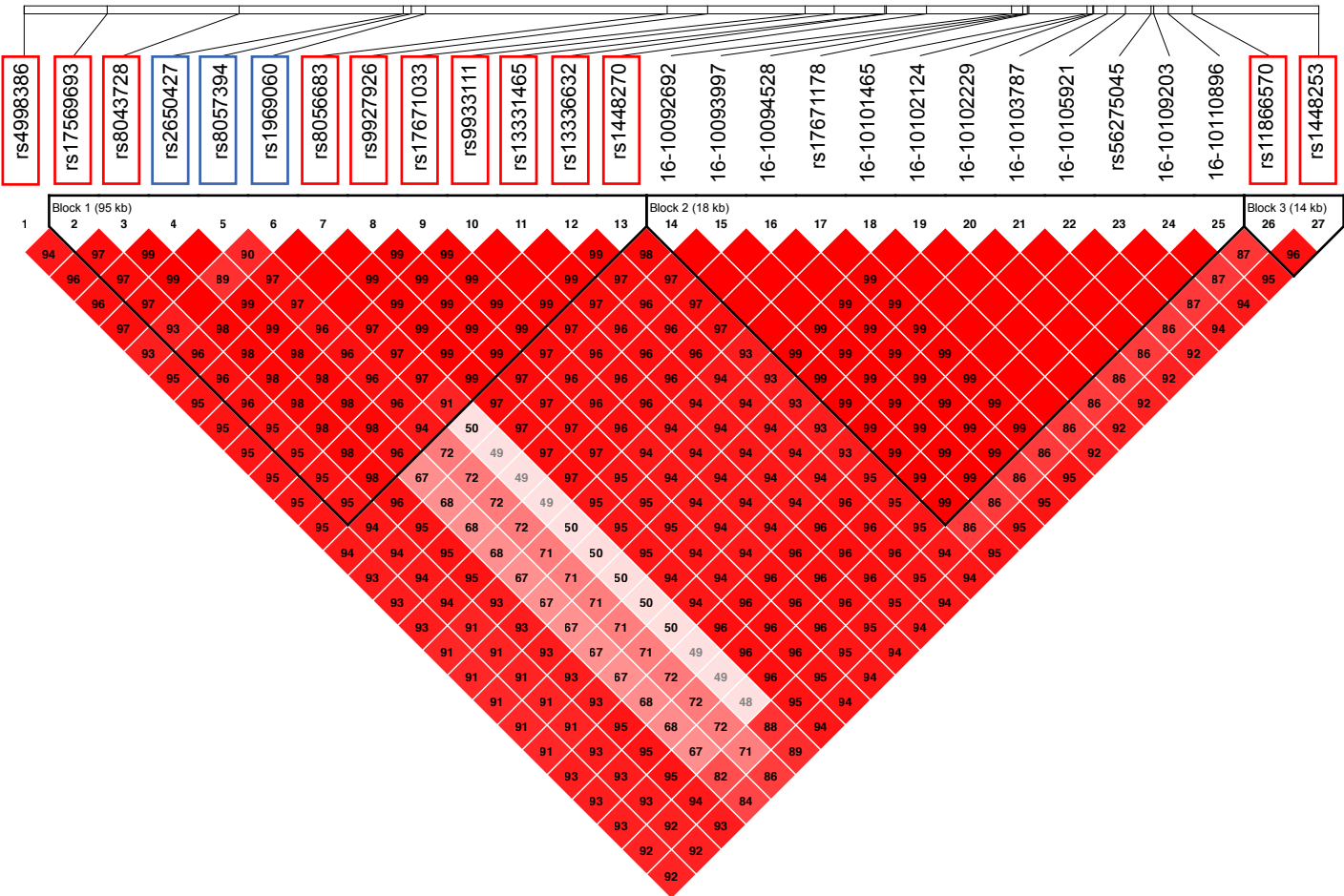

Supplement: Figure S4 — LD among the PD-associated and HD-associated SNPs. SNPs marked in red boxes were genotyped and achieved P<10−5 in either 2 df GWAIS or GWAS in heavy coffee drinkers with PD. SNPs not in boxes were imputed and achieved P≤5×10^−8^ in either 2 df GWAIS or GWAS in heavy coffee drinkers. SNPs in blue boxes are reported as being associated with HD [50], [51]. Panel A is r2, Panel B is D'. (PDF) [file pgen.1002237.s004.pdf]
