# Supplementary material for: Genome-Wide Gene-Environment Study Identifies Glutamate Receptor Gene GRIN2A as a Parkinson's Disease Modifier Gene via Interaction with Coffee
Source: PLoS Genet. 2011 Aug 18;7(8):e1002237. doi: 10.1371/journal.pgen.1002237 (PMC3158052; doi:10.1371/journal.pgen.1002237)
Supplement: Table S1 — Summary statistics on subject characteristics. NA: not available * Non-smoker: <100 cigarettes in lifetime. Smoker: ≥100 cigarettes. (DOC) [file pgen.1002237.s005.doc]

**Table S1 Summary statistics on subject characteristics.**

|  |  |  | **NGRC (Discovery)** | |  | **PEG (Replication)** | |  | **PAGE (Replication)** | |  | **HIHG (Replication)** | |
| --- | --- | --- | --- | --- | --- | --- | --- | --- | --- | --- | --- | --- | --- |
|  |  |  | **Case** | **Control** |  | **Case** | **Control** |  | **Case** | **Control** |  | **Case** | **Control** |
| **N** | Total |  | 1458 | 931 |  | 280 | 310 |  | 525 | 1474 |  | 209 | 133 |
|  |  |  |  |  |  |  |  |  |  |  |  |  |  |
| **Sex** | Male (%) |  | 991 (67.97) | 385 (41.35) |  | 156 (55.71) | 155 (50.00) |  | 406 (77.33) | 1164 (78.97) |  | 142 (67.94) | 56 (42.11) |
|  | Female (%) |  | 467 (32.03) | 546 (58.65) |  | 124 (44.29) | 155 (50.00) |  | 119 (22.67) | 310 (21.03) |  | 67 (32.06) | 77 (57.89) |
|  |  |  |  |  |  |  |  |  |  |  |  |  |  |
| ***GRIN2A*** **rs4998386**  **genotype** | CC (%) |  | 1227 (84.16) | 716 (76.91) |  | 234 (83.57) | 249 (80.32) |  | 434 (82.67) | 1199 (81.34) |  | 169 (80.86) | 110 (82.71) |
|  | TC (%) |  | 219 (15.02) | 204 (21.91) |  | 42 (15.00) | 55 (17.74) |  | 85 (16.19) | 267 (18.11) |  | 36 (17.22) | 22 (16.54) |
|  | TT (%) |  | 12 (0.82) | 11 (1.18) |  | 4 (1.43) | 6 (1.94) |  | 6 (1.14) | 8 (0.54) |  | 4 (1.91) | 1 (0.75) |
|  |  |  |  |  |  |  |  |  |  |  |  |  |  |
| **Caffeinated-coffee**  **drinking** | Light (%) |  | 946 (64.88) | 544 (58.43) |  | 184 (65.71) | 188 (60.65) |  | 290 (55.24) | 738 (50.07) |  | 147 (70.33) | 86 (64.66) |
|  | Heavy (%) |  | 512 (35.12) | 387 (41.57) |  | 96 (34.29) | 122 (39.35) |  | 235 (44.76) | 736 (49.93) |  | 62 (29.67) | 47 (35.34) |
|  |  |  |  |  |  |  |  |  |  |  |  |  |  |
| **Cigarette**  **smoking*** | Never (%) |  | 784 (54.18) | 486 (52.26) |  | 149 (54.18) | 121 (44.98) |  | 229 (44.29) | 523 (35.82) |  | 113 (54.33) | 66 (50.00) |
|  | Ever (%) |  | 663 (45.82) | 444 (47.74) |  | 126 (45.82) | 148 (55.02) |  | 288 (55.71) | 937 (64.18) |  | 95 (45.67) | 66 (50.00) |
|  |  |  |  |  |  |  |  |  |  |  |  |  |  |
| **Age at enrolment** | Mean±SD |  | 65.51±10.59 | 65.82±11.48 |  | 71.04±10.17 | 67.96±12.07 |  | 63.98±4.77 | 64.01±4.84 |  | 65.16±10.63 | 67.53±10.62 |
| **Age at onset** | Mean±SD |  | 58.13±11.61 | - |  | NA | - |  | 68.24±5.66 | - |  | 56.41±11.59 | - |
| **Age at diagnosis** | Mean±SD |  | 60.05±11.20 | - |  | 69.07±10.20 | - |  | 69.40±5.34 | - |  | NA | - |
