# Supplementary material for: Genome-Wide Gene-Environment Study Identifies Glutamate Receptor Gene GRIN2A as a Parkinson's Disease Modifier Gene via Interaction with Coffee
Source: PLoS Genet. 2011 Aug 18;7(8):e1002237. doi: 10.1371/journal.pgen.1002237 (PMC3158052; doi:10.1371/journal.pgen.1002237)
Supplement: Table S2 — Frequency of GRIN2A rs4998386_T and heavy coffee use in NGRC by disease strata and population structure. Family history: Patients who had at least one first or second degree relative with PD were classified as familial. All others were classified as non-familial (sporadic) Age at onset: The higher coffee consumption in late-onset PD (>50 years) is because they are older than patients who have early-onset PD and therefore have had higher cumulative lifetime coffee use over the years. Smoking: having smoked ≥100 cigarettes in the lifetime qualified as smoker (a standard criterion the literature). Coffee: Number of cups of caffeinated coffee drank per day multiplied by the number of years of consumption (ccy); heavy and light divided at the median in controls (67.5 ccy). Jewish/Non-Jewish clusters: The core of the Jewish cluster was defined within 0.04≤PC1≤0.055 and 0.001≤PC2≤0.013. A core within non-Jewish Caucasian cluster was defined within −0.0075≤PC1≤0.0025 & −0.005≤PC2≤0.003. See Hamza et al. [5]. Recruitment site: US states where subjects were recruited from. Paternal & maternal ancestry: Subjects whose both paternal and maternal ancestors came from the same country. Paternal or maternal ancestry: Since having only one lineage tracing back to a country was sufficient for this classification, an individual may fall in more than one group. * Adjusted for age. (DOC) [file pgen.1002237.s006.doc]

**Table S2 Frequency of *GRIN2A* rs4998386_T and heavy coffee use in NGRC by disease strata and population structure.**

|  |  | |  | ***GRIN2A* rs4998386_T** | | | |  | **Heavy Coffee Use** | | | |
| --- | --- | --- | --- | --- | --- | --- | --- | --- | --- | --- | --- | --- |
|  | Case  N (%) | Control  N (%) |  | MAF  Case | P | MAF  Control | P |  | Case  N (%) | P | Control  N (%) | P |
| All subjects | 1458 | 931 |  | 0.08 |  | 0.12 |  |  | 512 (35.12) |  | 387 (41.57) |  |
| **PD-Associated risk factors** | | | | | | | | | | | | |
| Sporadic PD | 1142 (78.33) | 931 (100.00) |  | 0.08 |  | 0.12 |  |  | 405 (35.46) |  | 387 (41.57) |  |
| Familial PD | 316 (21.67) | 931 (100.00) |  | 0.09 | 0.39 | 0.12 |  |  | 107 (33.86) | 0.60 | 387 (41.57) |  |
|  |  |  |  |  |  |  |  |  |  |  |  |  |
| Late onset (>50 yrs) | 1069 (73.32) | 931 (100.00) |  | 0.08 |  | 0.12 |  |  | 427 (39.94) |  | 387 (41.57) |  |
| Early onset (≤50 yrs) | 389 (26.68) | 931 (100.00) |  | 0.08 | 0.86 | 0.12 |  |  | 85 (21.85) | 0.19* | 387 (41.57) |  |
|  |  |  |  |  |  |  |  |  |  |  |  |  |
| Male | 991 (67.97) | 385 (41.35) |  | 0.08 |  | 0.13 |  |  | 371 (37.44) |  | 187 (48.57) |  |
| Female | 467 (32.03) | 546 (58.65) |  | 0.08 | 0.98 | 0.12 | 0.35 |  | 141 (30.19) | 0.01 | 200 (36.63) | 0.0003 |
|  |  |  |  |  |  |  |  |  |  |  |  |  |
| Smokers | 663 (45.47) | 444 (47.69) |  | 0.09 |  | 0.13 |  |  | 298 (44.95) |  | 233 (52.48) |  |
| Non-Smokers | 784 (53.77) | 486 (52.20) |  | 0.08 | 0.82 | 0.11 | 0.17 |  | 211 (26.91) | <0.0001 | 153 (31.48) | <0.0001 |
|  |  |  |  |  |  |  |  |  |  |  |  |  |
| Coffee-heavy | 512 (35.12) | 387 (41.57) |  | 0.07 |  | 0.14 |  |  |  |  |  |  |
| Coffee-light | 946 (64.88) | 544 (58.43) |  | 0.09 | 0.08 | 0.11 | 0.03 |  |  |  |  |  |
| **Ashkenazi Jewish (self-reported, verified by principal components)** | | | | | | |  |  |  |  |  |  |
| Yes | 67 (4.60) | 23 (2.47) |  | 0.03 |  | 0.04 |  |  | 17 (25.37) |  | 9 (39.13) |  |
| No | 1391 (95.40) | 908 (97.53) |  | 0.09 | 0.02 | 0.12 | 0.10 |  | 495 (35.59) | 0.09 | 378 (41.63) | 0.81 |
| **Recruitment Site** |  |  |  |  |  |  |  |  |  |  |  |  |
| New York | 346 (23.73) | 265 (28.46) |  | 0.08 |  | 0.11 |  |  | 118 (34.10) |  | 105 (39.62) |  |
| Oregon | 221 (15.16) | 130 (13.96) |  | 0.08 |  | 0.11 |  |  | 75 (33.94) |  | 55 (42.31) |  |
| Georgia | 220 (15.09) | 107 (11.49) |  | 0.08 |  | 0.11 |  |  | 82 (37.27) |  | 42 (39.25) |  |
| Washington | 671 (46.02) | 429 (46.08) |  | 0.09 | 0.81 | 0.13 | 0.48 |  | 237 (35.32) | 0.86 | 185 (43.12) | 0.78 |
| **Paternal and Maternal ancestry** | | | | | | | | | | | | |
| Great Britain | 100 (6.86) | 57 (6.12) |  | 0.14 |  | 0.16 |  |  | 35 (35.00) |  | 26 (45.61) |  |
| Germany / Austria | 76 (5.21) | 38 (4.08) |  | 0.07 |  | 0.20 |  |  | 31 (40.79) |  | 16 (42.11) |  |
| Ireland | 35 (2.40) | 14 (1.50) |  | 0.10 |  | 0.04 |  |  | 16 (45.71) |  | 6 (42.86) |  |
| Scandinavia | 44 (3.02) | 25 (2.69) |  | 0.14 |  | 0.10 |  |  | 18 (40.91) |  | 12 (48.00) |  |
| Eastern Europe | 27 (1.85) | 25 (2.69) |  | 0.02 |  | 0.12 |  |  | 12 (44.44) |  | 11 (44.00) |  |
| Italy | 43 (2.95) | 31 (3.33) |  | 0.05 |  | 0.06 |  |  | 22 (51.16) |  | 17 (54.84) |  |
| Russia | 16 (1.10) | 9 (0.97) |  | 0.09 | 0.03 | 0.06 | 0.19 |  | 4 (25.00) | 0.49 | 5 (55.56) | 0.95 |
| **Paternal or Maternal ancestry** | | | | | | | | | | | |  |
| Great Britain | 410 (28.12) | 262 (28.14) |  | 0.11 |  | 0.13 |  |  | 143 (34.88) |  | 108 (41.22) |  |
| Germany / Austria | 321 (22.02) | 193 (20.73) |  | 0.09 |  | 0.13 |  |  | 115 (35.83) |  | 71 (36.79) |  |
| Ireland | 180 (12.35) | 126 (13.53) |  | 0.08 |  | 0.13 |  |  | 72 (40.00) |  | 57 (45.24) |  |
| Scandinavia | 170 (11.66) | 102 (10.96) |  | 0.10 |  | 0.12 |  |  | 61 (35.88) |  | 46 (45.10) |  |
| Eastern Europe | 72 (4.94) | 60 (6.44) |  | 0.02 |  | 0.16 |  |  | 25 (34.72) |  | 27 (45.00) |  |
| Italy | 68 (4.66) | 59 (6.34) |  | 0.04 |  | 0.08 |  |  | 28 (41.18) |  | 21 (35.59) |  |
| France | 59 (4.05) | 45 (4.83) |  | 0.08 |  | 0.17 |  |  | 17 (28.81) |  | 14 (31.11) |  |
| Russia | 45 (3.09) | 18 (1.93) |  | 0.04 | 0.003 | 0.03 | 0.37 |  | 10 (22.22) | 0.40 | 9 (50.00) | 0.47 |
