# Supplementary material for: Genome-Wide Gene-Environment Study Identifies Glutamate Receptor Gene GRIN2A as a Parkinson's Disease Modifier Gene via Interaction with Coffee
Source: PLoS Genet. 2011 Aug 18;7(8):e1002237. doi: 10.1371/journal.pgen.1002237 (PMC3158052; doi:10.1371/journal.pgen.1002237)
Supplement: Table S3 — PD risk conditioned on GRIN2A genotype and coffee use (Expanded version of Table 3). (A) Heavy coffee use was associated with 27% risk reduction (1-OR) in the pooled data. (B) GRIN2A rs4998386_TC genotype was associated with reduced risk consistently across studies. rs4998386_TT frequency varied significantly across studies. (C) The a-priori hypothesis for replication that among heavy drinkers GRIN2A_rs4998386_T carriers had a lower risk of PD than GRIN2A_rs4998386_CC was replicated under three conditions: comparing TC to CC (excluding rare and variable TT genotype), Dominant model (TT+TC vs. CC) and Additive model (TT vs. TC vs CC). As predicted from Discovery phase, genotype had no effect on risk of PD among light coffee drinkers. (D) The joint effects of genotype and coffee showed a significant 59% drop in PD risk in people who had the rs4998386_TC genotype and were heavy drinkers, but little or no effect in other combinations. (E) A formal interaction test demonstrated that effects of coffee and genotype are dependent on each other. By definition, statistical interaction exists if the joint effect of gene (G) and exposure (E) is significantly different from the product of their individual effects. Interaction OR is the ratio of the OR of disease when g and e are present, divided by the product of the individual OR; i.e., ORinteraction = ORg+e/(ORg×ORe). (F) Dose-dependent risk reduction by coffee was clear and strong for rs4998386_TC genotype. Analyses were repeated with smoking added as covariate, results were unchanged (Table S4). OR: odds ratio. P: statistical significance, two sided for NGRC and pooled analysis, one-sided for replication studies. *Heterogeneity P: Breslow-Day test statistics to assess between-study heterogeneity conducted for coffee and genotypes and found to be significant only for TT genotype. Analyses were adjusted for sex and age at interview in each dataset, and also for study in the pooled analyses. (DOC) [file pgen.1002237.s007.doc]

| **Table S3** **PD risk conditioned on *GRIN2A* genotype and coffee use.** | | | | | | | | | | | | | | | | | | | | | | | | | |
| --- | --- | --- | --- | --- | --- | --- | --- | --- | --- | --- | --- | --- | --- | --- | --- | --- | --- | --- | --- | --- | --- | --- | --- | --- | --- |
| ***GRIN2A*** | **Coffee** | **NGRC**  **(Discovery)** | | | | **PEG**  **(Replication 1)** | | | | **PAGE**  **(Replication 2)** | | | | **HIHG**  **(Replication 3)** | | | | **Pooled**  **Replications** | | | | **Pooled**  **NGRC + Replications** | | | |
|  |  | **N Case** | **N Control** | **OR (SE)** | **P** | **N Case** | **N Control** | **OR (SE)** | **P** | **N Case** | **N Control** | **OR (SE)** | **P** | **N Case** | **N Control** | **OR (SE)** | **P** | **N Case** | **N Control** | **OR (SE)** | **P** | **N Case** | **N Control** | **OR (SE)** | **P** |
| **(A) Coffee irrespective of genotype** | | | | | | | | | | | | | | | | | | | | | | | | | |
| - | Light | 946 | 544 | Ref |  | 184 | 188 | Ref |  | 290 | 738 | Ref |  | 147 | 86 | Ref |  | 621 | 1012 | Ref |  | 1567 | 1556 | Ref |  |
| - | Heavy | 512 | 387 | 0.66(0.06) | 6x10^-6^ | 96 | 122 | 0.69(0.12) | 0.02 | 235 | 736 | 0.81(0.08) | 0.02 | 62 | 47 | 0.66(0.17) | 0.05 | 393 | 905 | 0.79(0.07) | 2x10^-3^ | 905 | 1292 | 0.73(0.04) | 3x10^-7^ |
| **(B) *GRIN2A* rs4998386 genotype irrespective of coffee** | | | | | | | | | | | | | | | | | | | | | | | | | |
| CC | - | 1227 | 716 | Ref |  | 234 | 249 | Ref |  | 434 | 1199 | Ref |  | 169 | 110 | Ref |  | 837 | 1558 | Ref |  | 2064 | 2274 | Ref |  |
| TC |  | 219 | 204 | 0.62(0.07) | 2x10^-5^ | 42 | 55 | 0.81(0.18) | 0.18 | 85 | 267 | 0.88(0.12) | 0.17 | 36 | 22 | 1.04(0.32) | 0.55 | 163 | 344 | 0.89(0.10) | 0.14 | 382 | 548 | 0.75(0.06) | 2x10^-4^ |
| TT |  | 12 | 11 | 0.53(0.23) | 0.15 | 4 | 6 |  |  | 6 | 8 |  |  | 4 | 1 |  |  | 14 | 15 |  |  | 26 | 26 | Heterogeneity P = 0.06* | |
| **(C) *GRIN2A* rs4998386 genotype stratified by coffee** | | | | | | | | | | | | | | | | | | | | | | | | | |
| CC | Heavy | 441 | 283 | Ref |  | 80 | 94 | Ref |  | 199 | 576 | Ref |  | 51 | 36 | Ref |  | 330 | 706 | Ref. |  | 771 | 989 | Ref |  |
| TC | Heavy | 69 | 99 | 0.42(0.08) | 2x10^-6^ | 14 | 27 | 0.62(0.23) | 0.10 | 31 | 154 | 0.58(0.12) | 0.01 | 9 | 11 | 0.64(0.33) | 0.19 | 54 | 192 | 0.59(0.10) | 1x10^-3^ | 123 | 291 | 0.51(0.06) | 7x10^-8^ |
| TT | Heavy | 2 | 5 | 0.19(0.16) | 0.05 | 2 | 1 |  |  | 5 | 6 |  |  | 2 | 0 |  |  | 9 | 7 |  |  | 11 | 12 | Heterogeneity P = 0.04* | |
|  |  |  |  |  |  |  |  |  |  |  |  |  |  |  |  |  |  |  |  |  |  |  |  |  |  |
| CC | Light | 786 | 433 | Ref |  | 154 | 155 | Ref |  | 235 | 623 | Ref |  | 118 | 74 | Ref. |  | 507 | 852 | Ref. |  | 1293 | 1285 | Ref |  |
| TC | Light | 150 | 105 | 0.81(0.12) | 0.16 | 28 | 28 | 0.97(0.29) | 0.46 | 54 | 113 | 1.27(0.23) | 0.90 | 27 | 11 | 1.36(0.56) | 0.77 | 109 | 152 | 1.24(0.18) | 0.93 | 259 | 257 | 1.00(0.10) | 0.99 |
| TT | Light | 10 | 6 | 0.81(0.44) | 0.70 | 2 | 5 |  |  | 1 | 2 |  |  | 2 | 1 |  |  | 5 | 8 |  |  | 15 | 14 |  |  |
| **Additive model** | | | | | | | | | | | | | | | | | | | | | | | | | |
| T / C | Heavy | 512 | 387 | 0.42(0.07) | 3x10^-7^ | 96 | 122 | 0.78(0.25) | 0.22 | 235 | 736 | 0.75(0.14) | 0.06 | 62 | 47 | 0.93(0.40) | 0.43 | 393 | 905 | 0.77(0.11) | 0.04 | 905 | 1292 | 0.60(0.07) | 4x10^-6^ |
| T / C | Light | 946 | 544 | 0.83(0.11) | 0.16 | 184 | 188 | 0.83(0.20) | 0.22 | 290 | 738 | 1.26(0.22) | 0.90 | 147 | 86 | 1.27(0.45) | 0.75 | 621 | 1012 | 1.15(0.15) | 0.85 | 1567 | 1556 | 0.98(0.09) | 0.79 |
| **Dominant model** | | | | | | | | | | | | | | | | | | | | | | | | | |
| CC | Heavy | 441 | 283 | Ref |  | 80 | 94 | Ref |  | 199 | 576 | Ref |  | 51 | 36 | Ref |  | 330 | 706 | Ref |  | 771 | 989 | Ref |  |
| T- | Heavy | 71 | 104 | 0.41(0.07) | 5x10^-7^ | 16 | 28 | 0.68(0.24) | 0.14 | 36 | 160 | 0.65(0.13) | 0.02 | 11 | 11 | 0.76(0.37) | 0.29 | 63 | 199 | 0.70(0.11) | 0.01 | 134 | 303 | 0.54(0.06) | 3x10^-7^ |
|  |  |  |  |  |  |  |  |  |  |  |  |  |  |  |  |  |  |  |  |  |  |  |  |  |  |
| CC | Light | 786 | 433 | Ref |  | 154 | 155 | Ref |  | 235 | 623 | Ref |  | 118 | 74 | Ref |  | 507 | 852 | Ref |  | 1293 | 1285 | Ref |  |
| T- | Light | 160 | 111 | 0.81(0.12) | 0.15 | 30 | 33 | 0.88(0.25) | 0.32 | 55 | 115 | 1.27(0.23) | 0.90 | 29 | 12 | 1.34(0.53) | 0.77 | 114 | 160 | 1.20(0.17) | 0.90 | 274 | 271 | 0.99(0.10) | 0.89 |
| **(D) Joint effects of *GRIN2A* rs4998386 and coffee** | | | | | | | | | | | | | | | | | | | | | | | | | |
| CC | Light | 786 | 433 | Ref. |  | 154 | 155 | Ref |  | 235 | 623 | Ref |  | 118 | 74 | Ref |  | 507 | 852 | Ref. |  | 1293 | 1285 | Ref |  |
| CC | Heavy | 441 | 283 | 0.75(0.08) | 6x10^-3^ | 80 | 94 | 0.73(0.14) | 0.05 | 199 | 576 | 0.92(0.10) | 0.22 | 51 | 36 | 0.73(0.21) | 0.13 | 330 | 706 | 0.88(0.08) | 0.08 | 771 | 989 | 0.82(0.06) | 3x10^-3^ |
| TC | Light | 150 | 105 | 0.81(0.12) | 0.15 | 28 | 28 | 0.99(0.29) | 0.48 | 54 | 113 | 1.27(0.23) | 0.10 | 27 | 11 | 1.39(0.56) | 0.21 | 109 | 152 | 1.24(0.18) | 0.07 | 259 | 257 | 1.00(0.10) | 0.99 |
| TC | Heavy | 69 | 99 | 0.32(0.06) | 7x10^-11^ | 14 | 27 | 0.46(0.16) | 0.01 | 31 | 154 | 0.54(0.11) | 2x10^-3^ | 9 | 11 | 0.49(0.24) | 0.08 | 54 | 192 | 0.52(0.09) | 5x10^-5^ | 123 | 291 | 0.41(0.05) | 6x10^-13^ |
| TT | Light | 10 | 6 | 0.81(0.44) | 0.70 | 2 | 5 |  |  | 1 | 2 |  |  | 2 | 1 |  |  | 5 | 8 |  |  | 15 | 14 |  |  |
| TT | Heavy | 2 | 5 | 0.14(0.12) | 0.02 | 2 | 1 |  |  | 5 | 6 |  |  | 2 | 0 |  |  | 9 | 7 |  |  | 11 | 12 |  |  |
| **(E) Interaction of *GRIN2A* rs4998386 genotypes and coffee consumption** | | | | | | | | | | | | | | | | | | | | | | | | | |
|  |  | 1446 | 920 | 0.52(0.12) | 4x10^-3^ | 276 | 304 | 0.65(0.31) | 0.18 | 519 | 1466 | 0.46(0.13) | 3x10^-3^ | 205 | 132 | 0.48(0.32) | 0.13 | 1000 | 1902 | 0.48(0.11) | 5x10^-4^ | 2446 | 2822 | 0.51(0.08) | 3x10^-5^ |
| **(F) Genotype specific dose-dependent effect of coffee** | | | | | | | | | | | | | | | | | | | | | | | | | |
| CC | ≤25 | 334 | 189 | Ref |  | 68 | 66 | Ref |  | 131 | 317 | Ref |  | 49 | 32 | Ref |  | 117 | 98 | Ref. |  | 451 | 287 | Ref |  |
|  | 25%-≤50% | 344 | 178 | 1.03(0.14) | 0.84 | 65 | 67 | 0.97(0.24) | 0.46 | 104 | 306 | 0.83(0.13) | 0.11 | 55 | 25 | 1.49(0.52) | 0.87 | 120 | 92 | 1.11(0.12) | 0.70 | 464 | 270 | 1.06(0.12) | 0.61 |
|  | 50%-≤75% | 366 | 203 | 0.91(0.12) | 0.47 | 54 | 58 | 0.88(0.23) | 0.31 | 86 | 296 | 0.71(0.11) | 0.02 | 37 | 29 | 0.72(0.26) | 0.18 | 91 | 87 | 0.83(0.17) | 0.19 | 457 | 290 | 0.89(0.10) | 0.30 |
|  | >75% | 183 | 146 | 0.58(0.09) | 3x10^-4^ | 47 | 58 | 0.68(0.18) | 0.07 | 113 | 280 | 0.99(0.15) | 0.47 | 28 | 24 | 0.58(0.23) | 0.09 | 75 | 82 | 0.68(0.15) | 0.04 | 258 | 228 | 0.61(0.08) | 6x10^-5^ |
|  |  |  |  |  |  |  |  |  |  |  |  |  |  |  |  |  |  |  |  |  |  |  |  |  |  |
| TC | ≤25% | 69 | 55 | Ref |  | 14 | 12 | Ref |  | 32 | 53 | Ref |  | 13 | 6 | Ref |  | 27 | 18 | Ref. |  | 96 | 73 | Ref |  |
|  | 25%-≤50% | 65 | 41 | 1.31(0.37) | 0.34 | 12 | 13 | 0.62(0.37) | 0.21 | 22 | 60 | 0.60(0.20) | 0.07 | 10 | 3 | 1.70(1.42) | 0.74 | 22 | 16 | 0.89(0.41) | 0.40 | 87 | 57 | 1.24(0.30) | 0.36 |
|  | 50%-≤75% | 59 | 56 | 0.71(0.20) | 0.21 | 9 | 15 | 0.30(0.19) | 0.03 | 15 | 68 | 0.36(0.13) | 3x10^-3^ | 7 | 7 | 0.47(0.35) | 0.15 | 16 | 22 | 0.40(0.19) | 0.03 | 75 | 78 | 0.63(0.15) | 0.05 |
|  | >75% | 26 | 52 | 0.31(0.10) | 2x10^-4^ | 7 | 15 | 0.36(0.23) | 0.06 | 16 | 86 | 0.31(0.11) | 5x10^-4^ | 6 | 6 | 0.57(0.46) | 0.24 | 13 | 21 | 0.37(0.18) | 0.02 | 39 | 73 | 0.34(0.09) | 5x10^-5^ |
|  |  |  |  |  |  |  |  |  |  |  |  |  |  |  |  |  |  |  |  |  |  |  |  |  |  |
| TT | ≤25% | 6 | 0 |  |  | 1 | 2 |  |  | 1 | 0 |  |  | 1 | 0 |  |  | 2 | 2 |  |  | 8 | 2 |  |  |
|  | 25%-≤50% | 2 | 3 |  |  | 0 | 2 |  |  | 0 | 2 |  |  | 0 | 1 |  |  | 0 | 3 |  |  | 2 | 6 |  |  |
|  | 50%-≤75% | 2 | 4 |  |  | 1 | 1 |  |  | 3 | 4 |  |  | 2 | 0 |  |  | 3 | 1 |  |  | 5 | 5 |  |  |
|  | >75% | 2 | 4 |  |  | 2 | 1 |  |  | 2 | 2 |  |  | 1 | 0 |  |  | 3 | 1 |  |  | 5 | 5 |  |  |
