# Supplementary material for: Genome-Wide Gene-Environment Study Identifies Glutamate Receptor Gene GRIN2A as a Parkinson's Disease Modifier Gene via Interaction with Coffee
Source: PLoS Genet. 2011 Aug 18;7(8):e1002237. doi: 10.1371/journal.pgen.1002237 (PMC3158052; doi:10.1371/journal.pgen.1002237)
Supplement: Table S4 — Smoking does not alter the results. GWAIS adjusted for smoking, sex, age, PC1, PC2 for [SNP+SNP*coffee] model gave P2df = 2×10^−6^, Pinteraction = 10−3. GWAS in heavy coffee-drinkers yielded OR = 0.44, P = 10−6. PD risk conditioned on GRIN2A_rs4998386 genotype and coffee use, adjusted for smoking as well as sex and age are given in Table S4. (DOC) [file pgen.1002237.s008.doc]

| **Table S4 Smoking does not alter the results.** | | | | | | | | | | | | | | | | | | | | | | | | | |
| --- | --- | --- | --- | --- | --- | --- | --- | --- | --- | --- | --- | --- | --- | --- | --- | --- | --- | --- | --- | --- | --- | --- | --- | --- | --- |
| ***GRIN2A*** | **Coffee** | **NGRC**  **(Discovery)** | | | | **PEG**  **(Replication 1)** | | | | **PAGE**  **(Replication 2)** | | | | **HIHG**  **(Replication 3)** | | | | **Pooled**  **Replications** | | | | **Pooled**  **NGRC+Replications** | | | |
|  |  | **N Case** | **N Control** | **OR (SE)** | **P** | **N Case** | **N Control** | **OR (SE)** | **P** | **N Case** | **N Control** | **OR (SE)** | **P** | **N Case** | **N Control** | **OR (SE)** | **P** | **N Case** | **N Control** | **OR (SE)** | **P** | **N Case** | **N Control** | **OR (SE)** | **P** |
| **(a) Coffee irrespective of genotype** | | | | | | | | | | | | | | | | | | | | | | | | | |
|  | Light | 938 | 544 | Ref |  | 181 | 159 | Ref |  | 285 | 731 | Ref |  | 147 | 85 | Ref |  | 613 | 975 | Ref |  | 1551 | 1519 | Ref |  |
| - | Heavy | 509 | 386 | 0.68(0.06) | 6x10^-5^ | 94 | 110 | 0.69(0.13) | 0.02 | 232 | 729 | 0.83(0.09) | 0.04 | 61 | 47 | 0.68(0.18) | 0.07 | 387 | 886 | 0.80(0.07) | 5x10^-3^ | 896 | 1272 | 0.75(0.05) | 5x10^-6^ |
| **(b) *GRIN2A* rs4998386 genotype irrespective of coffee** | | | | | | | | | | | | | | | | | | | | | | | | | |
| CC | - | 1216 | 716 | Ref |  | 229 | 215 | Ref |  | 429 | 1189 | Ref |  | 168 | 109 | Ref |  | 826 | 1513 | Ref |  | 2042 | 2229 | Ref |  |
| TC |  | 219 | 203 | 0.63(0.07) | 5x10^-5^ | 42 | 48 | 0.79(0.19) | 0.15 | 82 | 263 | 0.87(0.12) | 0.16 | 36 | 22 | 1.00(0.31) | 0.50 | 160 | 333 | 0.88(0.10) | 0.12 | 379 | 536 | 0.75(0.06) | 3x10^-4^ |
| TT | - | 12 | 11 | 0.54(0.24) | 0.16 | 4 | 6 | 0.56(0.37) | 0.19 | 6 | 8 | 2.34(1.27) | 0.94 | 4 | 1 | 2.00(2.27) | 0.73 | 14 | 15 | 1.46(0.58) | 0.83 | 26 | 26 | Heterogeneity P =.06 | |
| **(c) *GRIN2A* rs4998386 genotype stratified by coffee** | | | | | | | | | | | | | | | | | | | | | | | | | |
| CC | Heavy | 438 | 283 | Ref |  | 78 | 84 | Ref |  | 197 | 571 | Ref |  | 50 | 36 | Ref |  | 325 | 691 | Ref |  | 763 | 974 | Ref |  |
| TC | Heavy | 69 | 98 | 0.43(0.08) | 3x10^-6^ | 14 | 25 | 0.57(0.22) | 0.07 | 30 | 152 | 0.57(0.12) | 0.01 | 9 | 11 | 0.65(0.34) | 0.20 | 53 | 188 | 0.58(0.10) | 10^-3^ | 122 | 286 | 0.51(0.06) | 1x10^-7^ |
| TT | Heavy | 2 | 5 | 0.20(0.17) | 0.06 | 2 | 1 |  |  | 5 | 6 |  |  | 2 | 0 |  |  | 9 | 7 | 3.06(1.64) | 0.98 | 11 | 12 | Heterogeneity P =.04 | |
|  |  |  |  |  |  |  |  |  |  |  |  |  |  |  |  |  |  |  |  |  |  |  |  |  |  |
| CC | Light | 778 | 433 | Ref |  | 151 | 131 | Ref |  | 232 | 618 | Ref |  | 118 | 73 | Ref |  | 501 | 822 | Ref |  | 1279 | 1255 | Ref |  |
| TC | Light | 150 | 105 | 0.82(0.12) | 0.18 | 28 | 23 | 1.02(0.32) | 0.53 | 52 | 111 | 1.26(0.23) | 0.89 | 27 | 11 | 1.31(0.54) | 0.74 | 107 | 145 | 1.24(0.18) | 0.93 | 257 | 250 | 1.00(0.10) | 0.98 |
| TT | Light | 10 | 6 | 0.82(0.45) | 0.71 | 2 | 5 |  |  | 1 | 2 |  |  | 2 | 1 |  |  | 5 | 8 | 0.63(0.37) | 0.22 | 15 | 14 | 0.77(0.30) | 0.51 |
| **Additive** | | | | | | | | | | | | | | | | | | | | | | | | | |
| T vs. C | Heavy | 509 | 386 | 0.43(0.07) | 7x10^-7^ | 94 | 110 | 0.70(0.23) | 0.14 | 232 | 729 | 0.76(0.14) | 0.07 | 61 | 47 | 0.97(0.42) | 0.47 | 387 | 886 | 0.78(0.12) | 0.05 | 896 | 1272 | 0.60(0.07) | 9x10^-6^ |
| T vs. C | Light | 938 | 544 | 0.84(0.11) | 0.18 | 181 | 159 | 0.82(0.21) | 0.21 | 285 | 731 | 1.25(0.22) | 0.90 | 147 | 85 | 1.22(0.44) | 0.71 | 613 | 975 | 1.14(0.15) | 0.83 | 1551 | 1519 | 0.98(0.09) | 0.79 |
| **Dominant** | | | | | | | | | | | | | | | | | | | | | | | | | |
| CC | Heavy | 438 | 283 | Ref |  | 78 | 84 | Ref |  | 197 | 571 | Ref |  | 50 | 36 | Ref |  | 325 | 691 | Ref |  | 763 | 974 | Ref |  |
| T | Heavy | 71 | 103 | 0.42(0.07) | 9x10^-7^ | 16 | 26 | 0.61(0.23) | 0.09 | 35 | 158 | 0.65(0.13) | 0.02 | 11 | 11 | 0.79(0.39) | 0.32 | 61 | 195 | 0.66(0.11) | 6x10^-3^ | 133 | 298 | 0.54(0.07) | 5x10^-7^ |
|  |  |  |  |  |  |  |  |  |  |  |  |  |  |  |  |  |  |  |  |  |  |  |  |  |  |
|  |  |  |  |  |  |  |  |  |  |  |  |  |  |  |  |  |  |  |  |  |  |  |  |  |  |
| CC | Light | 778 | 433 | Ref |  | 151 | 131 | Ref |  | 232 | 618 | Ref |  | 118 | 73 | Ref |  | 501 | 822 | Ref |  | 1279 | 1255 | Ref |  |
| T | Light | 160 | 111 | 0.82(0.12) | 0.17 | 30 | 28 | 0.89(0.27) | 0.35 | 53 | 113 | 1.26(0.23) | 0.90 | 29 | 12 | 1.29(0.51) | 0.74 | 112 | 153 | 1.20(0.17) | 0.89 | 272 | 264 | 0.99(0.10) | 0.91 |
| **(d) Joint effects of *GRIN2A* rs4998386 and coffee** | | | | | | | | | | | | | | | | | | | | | | | | | |
| CC | Light | 778 | 433 | Ref |  | 151 | 131 | Ref |  | 232 | 618 | Ref |  | 118 | 73 | Ref |  | 501 | 822 | Ref |  | 1279 | 1255 | Ref |  |
| CC | Heavy | 438 | 283 | 0.78(0.08) | 0.02 | 78 | 84 | 0.73(0.15) | 0.07 | 197 | 571 | 0.94(0.11) | 0.29 | 50 | 36 | 0.75(0.21) | 0.16 | 325 | 691 | 0.90(0.08) | 0.12 | 763 | 974 | 0.84(0.06) | 0.01 |
| TC | Light | 150 | 105 | 0.82(0.12) | 0.18 | 28 | 23 | 0.99(0.31) | 0.49 | 52 | 111 | 1.26(0.23) | 0.89 | 27 | 11 | 1.30(0.52) | 0.74 | 107 | 145 | 1.23(0.18) | 0.92 | 257 | 250 | 1.00(0.10) | 0.99 |
| TC | Heavy | 69 | 98 | 0.33(0.06) | 7x10^-10^ | 14 | 25 | 0.44(0.16) | 0.01 | 30 | 152 | 0.54(0.12) | 2x10^-3^ | 9 | 11 | 0.51(0.25) | 0.09 | 53 | 188 | 0.52(0.09) | 7x10^-5^ | 122 | 286 | 0.43(0.05) | 8x10^-12^ |
| TT | Light | 10 | 6 | 0.82(0.45) | 0.71 | 2 | 5 |  |  | 1 | 2 |  |  | 2 | 1 |  |  | 5 | 8 | 0.65(0.39) | 0.24 | 15 | 14 | 0.79(0.31) | 0.54 |
| TT | Heavy | 2 | 5 | 0.15(0.13) | 0.02 | 2 | 1 |  |  | 5 | 6 |  |  | 2 | 0 |  |  | 9 | 7 | 2.60(1.38) | 0.96 | 11 | 12 | 1.08(0.50) | 0.86 |
| **(e) Interaction of *GRIN2A* rs4998386 genotype and coffee consumption** | | | | | | | | | | | | | | | | | | | | | | | | | |
|  | | 1435 | 919 | 0.52(0.12) | 5x10^-3^ | 271 | 263 | 0.61(0.30) | 0.16 | 511 | 1452 | 0.46(0.13) | 3x10^-3^ | 208 | 131 | 0.52(0.35) | 0.16 | 986 | 1846 | 0.47(0.11) | 5x10^-4^ | 2421 | 2765 | 0.50(0.08) | 3x10^-5^ |
| **(f) Genotype specific dose-dependent effect of coffee** | | | | | | | | | | | | | | | | | | | | | | | | | |
| CC | ≤25% | 332 | 189 | Ref |  | 67 | 59 | Ref |  | 130 | 312 | Ref |  | 49 | 32 | Ref |  | 116 | 91 | Ref |  | 448 | 280 | Ref |  |
|  | 25%-≤50% | 342 | 178 | 1.05(0.14) | 0.74 | 63 | 55 | 1.07(0.28) | 0.61 | 102 | 306 | 0.78(0.12) | 0.05 | 55 | 24 | 1.95(0.73) | 0.96 | 118 | 79 | 1.29(0.27) | 0.89 | 460 | 257 | 1.12(0.13) | 0.34 |
|  | 50%-≤75% | 362 | 203 | 0.93(0.13) | 0.61 | 54 | 51 | 0.95(0.26) | 0.43 | 84 | 292 | 0.68(0.11) | 0.01 | 37 | 29 | 0.88(0.33) | 0.37 | 91 | 80 | 0.93(0.20) | 0.37 | 453 | 283 | 0.94(0.11) | 0.58 |
|  | >75% | 180 | 146 | 0.60(0.09) | 1x10^-3^ | 45 | 50 | 0.76(0.21) | 0.16 | 113 | 279 | 1.01(0.16) | 0.52 | 27 | 24 | 0.71(0.29) | 0.20 | 72 | 74 | 0.76(0.18) | 0.12 | 252 | 220 | 0.64(0.08) | 6x10^-4^ |
|  |  |  |  |  |  |  |  |  |  |  |  |  |  |  |  |  |  |  |  |  |  |  |  |  |  |
| TC | ≤25% | 69 | 55 | Ref |  | 14 | 10 | Ref |  | 32 | 53 | Ref |  | 13 | 6 | Ref |  | 27 | 16 | Ref |  | 96 | 71 | Ref |  |
|  | 25%-≤50% | 65 | 41 | 1.35(0.39) | 0.29 | 12 | 10 | 0.77(0.49) | 0.34 | 20 | 58 | 0.56(0.19) | 0.05 | 10 | 3 | 1.62(1.39) | 0.71 | 22 | 23 | 1.04(0.51) | 0.53 | 87 | 54 | 1.33(0.32) | 0.24 |
|  | 50%-≤75% | 59 | 55 | 0.74(0.21) | 0.30 | 9 | 14 | 0.29(0.19) | 0.03 | 14 | 67 | 0.34(01.3) | 2x10^-3^ | 7 | 7 | 0.46(0.34) | 0.15 | 16 | 21 | 0.40(0.19) | 0.03 | 75 | 76 | 0.66(0.16) | 0.08 |
|  | >75% | 26 | 52 | 0.32(0.11) | 6x10^-4^ | 7 | 14 | 0.35(0.23) | 0.06 | 16 | 85 | 0.32(0.11) | 6x10^-4^ | 6 | 6 | 0.53(0.45) | 0.23 | 13 | 20 | 0.38(0.19) | 0.03 | 39 | 72 | 0.36(0.10) | 2x10^-4^ |
|  |  |  |  |  |  |  |  |  |  |  |  |  |  |  |  |  |  |  |  |  |  |  |  |  |  |
| TT | ≤25% | 6 | 0 |  |  | 1 | 2 |  |  | 1 | 0 |  |  | 1 | 0 |  |  | 2 | 2 |  |  | 8 | 2 | Ref |  |
|  | 25%-≤50% | 2 | 3 |  |  | 0 | 2 |  |  | 0 | 2 |  |  | 0 | 1 |  |  | 0 | 3 |  |  | 2 | 6 | 0.05(0.06) | 0.02 |
|  | 50%-≤75% | 2 | 4 |  |  | 1 | 1 |  |  | 3 | 4 |  |  | 2 | 0 |  |  | 3 | 1 |  |  | 5 | 5 | 0.21(0.23) | 0.16 |
|  | >75% | 2 | 4 |  |  | 2 | 1 |  |  | 2 | 2 |  |  | 1 | 0 |  |  | 3 | 1 |  |  | 5 | 5 | 0.32(0.36) | 0.31 |
