# Supplementary material for: Genome-Wide Gene-Environment Study Identifies Glutamate Receptor Gene GRIN2A as a Parkinson's Disease Modifier Gene via Interaction with Coffee
Source: PLoS Genet. 2011 Aug 18;7(8):e1002237. doi: 10.1371/journal.pgen.1002237 (PMC3158052; doi:10.1371/journal.pgen.1002237)
Supplement: Table S5 — Caffeinated soda and tea do not alter the results. GWAIS and stratified GWAS results were robust when caffeinated tea and soda were included as additional covariates, along with sex, age, PC1 and PC2. (DOC) [file pgen.1002237.s009.doc]

**Table S5 Caffeinated Soda and Tea do not alter the results.**

| **GWAIS** | | | | | | | | | |
| --- | --- | --- | --- | --- | --- | --- | --- | --- | --- |
| Adjusted for | Case | Control | SNP | | | Interaction | | | 2df |
|  |  |  | OR | SE | P | OR | SE | P | P |
| Dominant | | | | | | | | | |
| Tea | 1441 | 920 | 0.83 | 0.12 | 0.19 | 0.49 | 0.11 | 2.3x10^-3^ | 2.3x10^-6^ |
| Soda | 1444 | 915 | 0.86 | 0.12 | 0.30 | 0.47 | 0.11 | 1.2x10^-3^ | 2.3x10^-6^ |
| Tea & soda | 1427 | 908 | 0.85 | 0.12 | 0.27 | 0.48 | 0.11 | 1.8x10^-3^ | 3.8x10^-6^ |
|  |  |  |  |  |  |  |  |  |  |
| Additive | | | | | | | | | |
| Tea | 1441 | 920 | 0.85 | 0.11 | 0.24 | 0.50 | 0.11 | 1.3x10^-3^ | 2.1x10^-6^ |
| Soda | 1444 | 915 | 0.88 | 0.12 | 0.35 | 0.48 | 0.10 | 6.4x10^-4^ | 1.7x10^-6^ |
| Tea & soda | 1427 | 908 | 0.87 | 0.12 | 0.31 | 0.49 | 0.11 | 1.0x10^-3^ | 3.1x10^-6^ |

| **Stratified GWAS** | | | | | | | | | | |
| --- | --- | --- | --- | --- | --- | --- | --- | --- | --- | --- |
| Adjusted for | Case | Control |  | Dominant | | |  | Additive | | |
|  |  |  |  | OR | SE | P |  | OR | SE | P |
| Heavy coffee drinker | | | | | | | | | | |
| Tea | 507 | 384 |  | 0.42 | 0.08 | 1.6x10^-6^ |  | 0.43 | 0.13 | 1.2x10^-6^ |
| Soda | 508 | 383 |  | 0.41 | 0.07 | 1.0x10^-6^ |  | 0.43 | 0.07 | 7.1x10^-7^ |
| Tea & soda | 503 | 380 |  | 0.42 | 0.08 | 1.9x10^-6^ |  | 0.43 | 0.08 | 1.4x10^-6^ |
|  |  |  |  |  |  |  |  |  |  |  |
| Light coffee drinker | | | | | | | | | | |
| Tea | 934 | 536 |  | 0.83 | 0.12 | 0.20 |  | 0.85 | 0.11 | 0.24 |
| Soda | 936 | 532 |  | 0.86 | 0.13 | 0.30 |  | 0.88 | 0.12 | 0.35 |
| Tea & soda | 924 | 528 |  | 0.85 | 0.13 | 0.27 |  | 0.87 | 0.12 | 0.32 |

Given the small effect that tea and soda had on the results, we questioned if soda and tea, both of which contain caffeine, had any effect on PD risk. The amount of caffeine in an 8 oz drink is approximately 10-30 mg in soda, 40-120 mg in black tea, and 100-200 mg in drip or brewed coffee. The following is the OR and P values for the association of tea and coffee with PD risk in (a) the full GWAIS model, and (b) tested individually without coffee, SNP and interaction in the model but adjusted for age, sex and PC1 and PC2. Results are consistent with an inverse association with PD in line with the caffeine content of each drink

|  | (a) PD-association in GWAIS with coffee, SNP, SNP*coffee, sex, age, PC1 and PC2 in the model | |  | (b) PD-association adjusted for sex, age, PC1 and PC2 | |
| --- | --- | --- | --- | --- | --- |
|  | OR | P |  | OR | P |
| Tea | 0.81 | 0.03 |  | 0.80 | 0.02 |
| Soda | 0.89 | 0.29 |  | 0.87 | 0.20 |
