# Supplementary material for: Genome-Wide Gene-Environment Study Identifies Glutamate Receptor Gene GRIN2A as a Parkinson's Disease Modifier Gene via Interaction with Coffee
Source: PLoS Genet. 2011 Aug 18;7(8):e1002237. doi: 10.1371/journal.pgen.1002237 (PMC3158052; doi:10.1371/journal.pgen.1002237)
Supplement: Table S6 — Exploring characteristics of individuals with the rare TT genotype in search of the source of heterogeneity. There was no trend for any of the PD-relevant characteristics that would explain the heterogeneity in TT genotype across studies. Due to the low frequency of TT and the small number of subjects of Jewish heritage, the N = 0 for the Jewish subgroup is expected. (DOC) [file pgen.1002237.s010.doc]

| **Table S6 Exploring characteristics of individuals with the rare TT genotype in search of the source of heterogeneity.** | | | | | | | | | | | | |
| --- | --- | --- | --- | --- | --- | --- | --- | --- | --- | --- | --- | --- |
|  | **NGRC** | | | **PEG** | | | **PAGE** | | | **HIHG** | | |
| **rs4998386_T** | **Total**  **(freq)** | **Case**  **(freq)** | **Control**  **(freq)** | **Total**  **(freq)** | **Case**  **(freq)** | **Control**  **(freq)** | **Total**  **(freq)** | **Case**  **(freq)** | **Control**  **(freq)** | **Total**  **(freq)** | **Case**  **(freq)** | **Control**  **(freq)** |
| Total N individuals | 2389 | 1458 | 931 | 590 | 280 | 310 | 1999 | 525 | 1474 | 342 | 209 | 133 |
| N individual with TT | 23 (0.010) | 12 (0.008) | 11 (0.012) | 10 (0.016) | 4 (0.014) | 6 (0.018) | 14 (0.007) | 6 (0.011) | 8 (0.005) | 5 (0.015) | 4 (0.019) | 1 (0.008) |
| MAF | 0.098 | 0.083 | 0.121 | 0.099 | 0.089 | 0.108 | 0.095 | 0.092 | 0.096 | 0.099 | 0.105 | 0.090 |
| PD subtype | | | | | | | | | | | | |
| Sporadic PD | - | 9 (0.008) | - | - | 4 (0.014) | - | - | 6 (0.013) | - | - | 2 (0.010) | - |
| Familial PD | - | 3 (0.009) | - | - | 0 (0.000) | - | - | 0 (0.000) | - | - | 2 (0.010) | - |
| Late Onset (>50 yrs) | - | 9 (0.008) | - | - | 4 (0.014) | - | - | 6 (0.014) | - | - | 3 (0.014) | - |
| Early Onset (≤50 yrs) | - | 3 (0.008) | - | - | 0 (0.000) | - | - | 0 (0.000) | - | - | 1 (0.005) | - |
| Characteristics relevant to PD risk | | | | | | | | | | | | |
| Male | 16 (0.012) | 8 (0.008) | 8 (0.021) | 3 (0.005) | 1 (0.004) | 2 (0.006) | 11 (0.007) | 6 (0.015) | 5 (0.004) | 4 (0.020) | 4 (0.028) | 0 (0.000) |
| Female | 7 (0.007) | 4 (0.009) | 3 (0.005) | 7 (0.011) | 3 (0.011) | 4 (0.012) | 3 (0.007) | 0 (0.000) | 3 (0.0097) | 1 (0.007) | 0 (0.000) | 1 (0.013) |
| Smokers | 12 (0.011) | 4 (0.006) | 8 (0.018) | 4 (0.007) | 2 (0.007) | 2 (0.006) | 13 (0.011) | 6 (0.002) | 7 (0.0075) | 3 (0.019) | 3 (0.032) | 0 (0.000) |
| Non-Smokers | 11 (0.009) | 8 (0.010) | 3 (0.006) | 6 (0.010) | 2 (0.007) | 4 (0.012) | 1 (0.001) | 0 (0.000) | 1 (0.0019) | 2 (0.011) | 1 (0.009) | 1 (0.015) |
| Coffee Heavy | 7 (0.008) | 2 (0.004) | 5 (0.013) | 3 (0.005) | 2 (0.007) | 1 (0.003) | 11 (0.011) | 5 (0.021) | 6 (0.008) | 2 (0.018) | 2 (0.032) | 0 (0.000) |
| Coffee Light | 16 (0.011) | 10 (0.011) | 6 (0.011) | 7 (0.011) | 2 (0.007) | 5 (0.015) | 3 (0.003) | 1 (0.003) | 2 (0.003) | 2 (0.009) | 1 (0.007) | 1 (0.011) |
| Ashkenazi Jewish Yes | 0 (0.000) | 0 (0.000) | 0 (0.000) | - | - | - | - | - | - | 0 (0.000) | 0 (0.000) | 0 (0.000) |
| Ashkenazi Jewish No | 23 (0.010) | 12 (0.008) | 11 (0.012) | - | - | - | - | - | - | 5 (0.015) | 4 (0.012) | 1 (0.003) |
| Recruitment site | | | | | | | | | | | | |
| New York | 6 (0.010) | 4 (0.012) | 2 (0.008) | - | - | - | - | - | - | 0 (0.000) | 0 (0.000) | 0 (0.000) |
| Oregon | 1 (0.003) | 1 (0.005) | 0 (0.000) | - | - | - | - | - | - | 0 (0.000) | 0 (0.000) | 0 (0.000) |
| Georgia | 2 (0.006) | 1 (0.005) | 1 (0.009) | - | - | - | - | - | - | 1 (0.500) | 1 (1.00) | 0 (0.000) |
| Washington | 14 (0.013) | 6 (0.009) | 8 (0.190) | - | - | - | - | - | - | 0 (0.000) | 0 (0.000) | 0 (0.000) |
| North Carolina | - | - | - | - | - | - | - | - | - | 4 (0.019) | 3 (0.020) | 1 (0.017) |
| Fresno county, California | - | - | - | 5 (0.008) | 1 (0.004) | 4 (0.012) | - | - | - | - | - | - |
| Kern county, California | - | - | - | 3 (0.005) | 1 (0.004) | 2 (0.006) | - | - | - | - | - | - |
| Tulare County, California | - | - | - | 2 (0.003) | 2 (0.007) | 0 (0.000) | - | - | - | - | - | - |
| Paternal and Maternal Ancestry | | | | | | | | | | | | |
| Great Britain | 2 (0.013) | 2 (0.020) | 0 (0.000) | - | - | - | - | - | - | - | - | - |
| Germany / Austria | 1 (0.009) | 1 (0.013) | 0 (0.000) | - | - | - | - | - | - | - | - | - |
| Ireland | 0 (0.000) | 0 (0.000) | 0 (0.000) | - | - | - | - | - | - | - | - | - |
| Scandinavia | 0 (0.000) | 0 (0.000) | 0 (0.000) | - | - | - | - | - | - | - | - | - |
| Eastern Europe | 0 (0.000) | 0 (0.000) | 0 (0.000) | - | - | - | - | - | - | - | - | - |
| Italy | 0 (0.000) | 0 (0.000) | 0 (0.000) | - | - | - | - | - | - | - | - | - |
| Russia | 0 (0.000) | 0 (0.000) | 0 (0.000) | - | - | - | - | - | - | - | - | - |
| Paternal or Maternal Ancestry | | | | | | | | | | | | |
| Great Britain | 10 (0.015) | 5 (0.012) | 5 (0.019) | - | - | - | - | - | - | - | - | - |
| Germany / Austria | 5 (0.010) | 3 (0.009) | 2 (0.010) | - | - | - | - | - | - | - | - | - |
| Ireland | 3 (0.010) | 0 (0.000) | 3 (0.024) | 2 (0.003) | 1 (0.004) | 1 (0.003) | - | - | - | - | - | - |
| Scandinavia | 1 (0.004) | 0 (0.000) | 1 (0.010) | 3 (0.005) | 0 (0.000) | 3 (0.009) | - | - | - | - | - | - |
| Eastern Europe | 1 (0.008) | 0 (0.000) | 1 (0.017) | - | - | - | - | - | - | - | - | - |
| Italy | 1 (0.008) | 0 (0.000) | 1 (0.017) | - | - | - | - | - | - | - | - | - |
| France | 1 (0.008) | 0 (0.000) | 1 (0.017) | - | - | - | - | - | - | - | - | - |
| Russia | 0 (0.000) | 0 (0.000) | 0 (0.000) | - | - | - | - | - | - | - | - | - |
| Switzerland | - | - | - | 1 (0.002) | 1 (0.004) | 0 (0.000) | - | - | - | - | - | - |
