# Supplementary material for: Genome-Wide Gene-Environment Study Identifies Glutamate Receptor Gene GRIN2A as a Parkinson's Disease Modifier Gene via Interaction with Coffee
Source: PLoS Genet. 2011 Aug 18;7(8):e1002237. doi: 10.1371/journal.pgen.1002237 (PMC3158052; doi:10.1371/journal.pgen.1002237)
Supplement: Table S7 — Side-by-side comparison of results from GWAS, Interaction, GWAIS and stratified GWAS analyses for the top GRIN2A SNPs. Genotyped and imputed SNPs (info score ≥95%) that reached P<10−5 in GWAIS are shown in the order of base pair position (BP). (DOC) [file pgen.1002237.s011.doc]

| **Table S7 Side-by-side comparison of results from different analyses for the top *GRIN2A* SNPs.** | | | | | | | | | | | | | | |
| --- | --- | --- | --- | --- | --- | --- | --- | --- | --- | --- | --- | --- | --- | --- |
| **SNP** | **BP** | **All samples with coffee data (Heavy or Light)**  **N=2389** | | | | | | |  | **Heavy coffee drinkers**  **N=899** | |  | **Light coffee drinkers**  **N=1490** | |
|  |  | **GWAS**  **Main effect**  **1df** | |  | **Interaction**  **1df** | |  | **GWAIS**  **Joint test**  **2df** |  | **GWAS**  **Main effect 1df** | |  | **GWAS**  **Main effect 1df** | |
|  |  | **OR (SE)** | **P** |  | **OR (SE)** | **P** |  | **P** |  | **OR (SE)** | **P** |  | **OR (SE)** | **P** |
| 16-9942563 | 9942563 | 0.67 (0.07) | 2x10^-4^ |  | 0.53 (0.11) | 3x10^-3^ |  | 6x10^-6^ |  | 0.45 (0.08) | 4x10^-6^ |  | 0.85 (0.11) | 0.21 |
| rs57576479 | 9945823 | 0.67 (0.07) | 1x10^-4^ |  | 0.53 (0.11) | 3x10^-3^ |  | 5x10^-6^ |  | 0.46 (0.08) | 4x10^-6^ |  | 0.85 (0.11) | 0.21 |
| rs59775432 | 9954194 | 0.67 (0.07) | 8x10^-5^ |  | 0.52 (0.11) | 2x10^-3^ |  | 3x10^-6^ |  | 0.45 (0.08) | 3x10^-6^ |  | 0.85 (0.11) | 0.22 |
| rs7192121 | 9956084 | 0.67 (0.07) | 8x10^-5^ |  | 0.51 (0.11) | 2x10^-3^ |  | 3x10^-6^ |  | 0.44 (0.08) | 2x10^-6^ |  | 0.85 (0.11) | 0.23 |
| rs17670318 | 9957403 | 0.67 (0.07) | 8x10^-5^ |  | 0.51 (0.11) | 2x10^-3^ |  | 2x10^-6^ |  | 0.44 (0.08) | 2x10^-6^ |  | 0.86 (0.11) | 0.24 |
| rs9925439 | 9962962 | 0.67 (0.07) | 8x10^-5^ |  | 0.51 (0.11) | 1x10^-3^ |  | 2x10^-6^ |  | 0.44 (0.08) | 2x10^-6^ |  | 0.86 (0.11) | 0.24 |
| rs17670396 | 9966823 | 0.67 (0.07) | 7x10^-5^ |  | 0.51 (0.11) | 2x10^-3^ |  | 2x10^-6^ |  | 0.44 (0.08) | 2x10^-6^ |  | 0.85 (0.11) | 0.23 |
| rs7197048 | 9970116 | 0.67 (0.07) | 6x10^-5^ |  | 0.52 (0.11) | 2x10^-3^ |  | 2x10^-6^ |  | 0.45 (0.08) | 2x10^-6^ |  | 0.84 (0.11) | 0.19 |
| 16-9970628 | 9970628 | 0.67 (0.07) | 5x10^-5^ |  | 0.53 (0.11) | 3x10^-3^ |  | 3x10^-6^ |  | 0.45 (0.08) | 3x10^-6^ |  | 0.83 (0.11) | 0.17 |
| 16-9971030 | 9971030 | 0.67 (0.07) | 5x10^-5^ |  | 0.54 (0.11) | 3x10^-3^ |  | 3x10^-6^ |  | 0.45 (0.08) | 3x10^-6^ |  | 0.83 (0.11) | 0.16 |
| rs8045558 | 9972307 | 0.67 (0.07) | 4x10^-5^ |  | 0.53 (0.11) | 3x10^-3^ |  | 2x10^-6^ |  | 0.45 (0.08) | 2x10^-6^ |  | 0.83 (0.11) | 0.16 |
| 16-9973459 | 9973459 | 0.65 (0.07) | 4x10^-5^ |  | 0.52 (0.11) | 3x10^-3^ |  | 2x10^-6^ |  | 0.44 (0.08) | 2x10^-6^ |  | 0.83 (0.11) | 0.16 |
| rs57497843 | 9973912 | 0.65 (0.07) | 3x10^-5^ |  | 0.52 (0.11) | 2x10^-3^ |  | 2x10^-6^ |  | 0.44 (0.08) | 2x10^-6^ |  | 0.83 (0.11) | 0.17 |
| 16-9975154 | 9975154 | 0.65 (0.07) | 3x10^-5^ |  | 0.52 (0.11) | 2x10^-3^ |  | 1x10^-6^ |  | 0.44 (0.07) | 1x10^-6^ |  | 0.83 (0.11) | 0.17 |
| rs4998386 | 9978046 | 0.65 (0.07) | 3x10^-5^ |  | 0.50 (0.11) | 1x10^-3^ |  | 1x10^-6^ |  | 0.43 (0.07) | 6x10^-7^ |  | 0.84 (0.11) | 0.19 |
| rs7191736 | 9978079 | 0.65 (0.07) | 3x10^-5^ |  | 0.51 (0.11) | 2x10^-3^ |  | 9x10^-7^ |  | 0.43 (0.07) | 9x10^-7^ |  | 0.83 (0.11) | 0.17 |
| rs7190716 | 9978240 | 0.65 (0.07) | 3x10^-5^ |  | 0.51 (0.11) | 2x10^-3^ |  | 9x10^-7^ |  | 0.43 (0.07) | 9x10^-7^ |  | 0.83 (0.11) | 0.17 |
| rs4254328 | 9978571 | 0.65 (0.07) | 2x10^-5^ |  | 0.51 (0.11) | 2x10^-3^ |  | 9x10^-7^ |  | 0.43 (0.07) | 9x10^-7^ |  | 0.83 (0.11) | 0.17 |
| 16-9979028 | 9979028 | 0.65 (0.07) | 2x10^-5^ |  | 0.51 (0.11) | 2x10^-3^ |  | 8x10^-7^ |  | 0.43 (0.07) | 8x10^-7^ |  | 0.83 (0.11) | 0.16 |
| rs17569609 | 9979290 | 0.65 (0.07) | 2x10^-5^ |  | 0.51 (0.11) | 2x10^-3^ |  | 8x10^-7^ |  | 0.43 (0.07) | 8x10^-7^ |  | 0.83 (0.11) | 0.16 |
| rs3897996 | 9980130 | 0.64 (0.07) | 2x10^-5^ |  | 0.51 (0.11) | 2x10^-3^ |  | 7x10^-7^ |  | 0.43 (0.07) | 7x10^-7^ |  | 0.83 (0.11) | 0.15 |
| 16-9980602 | 9980602 | 0.64 (0.07) | 2x10^-5^ |  | 0.51 (0.11) | 2x10^-3^ |  | 6x10^-7^ |  | 0.43 (0.07) | 7x10^-7^ |  | 0.83 (0.11) | 0.15 |
| 16-9981345 | 9981345 | 0.65 (0.07) | 2x10^-5^ |  | 0.53 (0.11) | 2x10^-3^ |  | 7x10^-7^ |  | 0.44 (0.07) | 9x10^-7^ |  | 0.82 (0.11) | 0.13 |
| rs9929020 | 9981643 | 0.65 (0.07) | 2x10^-5^ |  | 0.53 (0.11) | 2x10^-3^ |  | 7x10^-7^ |  | 0.44 (0.07) | 9x10^-7^ |  | 0.82 (0.11) | 0.13 |
| 16-9982616 | 9982616 | 0.65 (0.07) | 2x10^-5^ |  | 0.53 (0.11) | 2x10^-3^ |  | 7x10^-7^ |  | 0.44 (0.07) | 9x10^-7^ |  | 0.82 (0.11) | 0.14 |
| 16-9982918 | 9982918 | 0.65 (0.07) | 2x10^-5^ |  | 0.52 (0.11) | 2x10^-3^ |  | 7x10^-7^ |  | 0.44 (0.07) | 9x10^-7^ |  | 0.83 (0.11) | 0.14 |
| rs17670509 | 9983495 | 0.65 (0.07) | 2x10^-5^ |  | 0.52 (0.11) | 2x10^-3^ |  | 7x10^-7^ |  | 0.44 (0.07) | 9x10^-7^ |  | 0.83 (0.11) | 0.14 |
| 16-9984703 | 9984703 | 0.64 (0.07) | 2x10^-5^ |  | 0.52 (0.11) | 2x10^-3^ |  | 6x10^-7^ |  | 0.43 (0.07) | 9x10^-7^ |  | 0.82 (0.11) | 0.12 |
| rs8044626 | 9986196 | 0.65 (0.07) | 2x10^-5^ |  | 0.52 (0.11) | 2x10^-3^ |  | 7x10^-7^ |  | 0.44 (0.07) | 9x10^-7^ |  | 0.83 (0.11) | 0.14 |
| rs7190619 | 9986625 | 0.65 (0.07) | 2x10^-5^ |  | 0.52 (0.11) | 2x10^-3^ |  | 7x10^-7^ |  | 0.44 (0.07) | 9x10^-7^ |  | 0.83 (0.11) | 0.14 |
| rs17670544 | 9987533 | 0.64 (0.07) | 2x10^-5^ |  | 0.55 (0.12) | 6x10^-3^ |  | 2x10^-6^ |  | 0.44 (0.08) | 3x10^-6^ |  | 0.79 (0.10) | 0.08 |
| rs17569693 | 9987686 | 0.64 (0.07) | 2X10^-5^ |  | 0.54 (0.12) | 5x10^-3^ |  | 2x10^-6^ |  | 0.44 (0.08) | 3x10^-6^ |  | 0.84 (0.11) | 0.09 |
| rs61628643 | 9988786 | 0.65 (0.07) | 2x10^-5^ |  | 0.52 (0.11) | 2x10^-3^ |  | 7x10^-7^ |  | 0.44 (0.07) | 9x10^-7^ |  | 0.83 (0.11) | 0.14 |
| rs59931374 | 9988788 | 0.65 (0.07) | 2x10^-5^ |  | 0.52 (0.11) | 2x10^-3^ |  | 7x10^-7^ |  | 0.44 (0.07) | 9x10^-7^ |  | 0.83 (0.11) | 0.14 |
| 16-9991621 | 9991621 | 0.65 (0.07) | 2x10^-5^ |  | 0.53 (0.11) | 2x10^-3^ |  | 8x10^-7^ |  | 0.44 (0.07) | 9x10^-7^ |  | 0.83 (0.11) | 0.14 |
| 16-9994644 | 9994644 | 0.65 (0.07) | 2x10^-5^ |  | 0.53 (0.11) | 2x10^-3^ |  | 9x10^-7^ |  | 0.44 (0.07) | 1x10^-6^ |  | 0.83 (0.11) | 0.14 |
| rs60547225 | 9996064 | 0.65 (0.07) | 2x10^-5^ |  | 0.53 (0.11) | 2x10^-3^ |  | 9x10^-7^ |  | 0.44 (0.07) | 1x10^-6^ |  | 0.82 (0.11) | 0.14 |
| 16-9999095 | 9999095 | 0.65 (0.07) | 2x10^-5^ |  | 0.53 (0.11) | 3x10^-3^ |  | 1x10^-6^ |  | 0.45 (0.07) | 2x10^-6^ |  | 0.82 (0.11) | 0.13 |
| 16-9999864 | 9999864 | 0.66 (0.07) | 3x10^-5^ |  | 0.55 (0.11) | 4x10^-3^ |  | 2x10^-6^ |  | 0.46 (0.08) | 3x10^-6^ |  | 0.82 (0.11) | 0.13 |
| 16-10000299 | 10000299 | 0.66 (0.07) | 4x10^-5^ |  | 0.55 (0.12) | 4x10^-3^ |  | 3x10^-6^ |  | 0.46 (0.08) | 3x10^-6^ |  | 0.82 (0.11) | 0.13 |
| 16-10000942 | 10000942 | 0.66 (0.07) | 4x10^-5^ |  | 0.56 (0.12 | 5x10^-3^ |  | 3x10^-6^ |  | 0.46 (0.08) | 4x10^-6^ |  | 0.82 (0.11) | 0.13 |
| 16-10001082 | 10001082 | 0.66 (0.07) | 4x10^-5^ |  | 0.56 (0.12) | 5x10^-3^ |  | 3x10^-6^ |  | 0.46 (0.08) | 4x10^-6^ |  | 0.82 (0.11) | 0.13 |
| 16-10001459 | 10001459 | 0.67 (0.07) | 4x10^-5^ |  | 0.56 (0.12) | 5x10^-3^ |  | 4x10^-6^ |  | 0.47 (0.08) | 4x10^-6^ |  | 0.82 (0.11) | 0.13 |
| rs7200719 | 10002047 | 0.67 (0.07) | 5x10^-5^ |  | 0.56 (0.12) | 5x10^-3^ |  | 4x10^-6^ |  | 0.47 (0.08) | 5x10^-6^ |  | 0.82 (0.11) | 0.13 |
| rs4107019 | 10002700 | 0.67 (0.07) | 5x10^-5^ |  | 0.56 (0.12) | 6x10^-3^ |  | 5x10^-6^ |  | 0.47 (0.08) | 6x10^-6^ |  | 0.82 (0.11) | 0.13 |
| rs8043728 | 10003004 | 0.67 (0.07) | 6X10^-5^ |  | 0.57 (0.12) | 0.01 |  | 8x10^-6^ |  | 0.44 (0.08) | 7x10^-6^ |  | 0.84 (0.11) | 0.13 |
| 16-10005429 | 10005429 | 0.67 (0.07) | 5x10^-5^ |  | 0.56 (0.12) | 6x10^-3^ |  | 5x10^-6^ |  | 0.47 (0.08) | 5x10^-6^ |  | 0.82 (0.11) | 0.13 |
| rs7203512 | 10006587 | 0.67 (0.07) | 5x10^-5^ |  | 0.56 (0.12) | 5x10^-3^ |  | 4x10^-6^ |  | 0.47 (0.08) | 5x10^-6^ |  | 0.82 (0.11) | 0.13 |
| rs7198528 | 10006596 | 0.67 (0.07) | 5x10^-5^ |  | 0.56 (0.12) | 5x10^-3^ |  | 4x10^-6^ |  | 0.47 (0.08) | 5x10^-6^ |  | 0.82 (0.11) | 0.13 |
| 16-10008653 | 10008653 | 0.67 (0.07) | 5x10^-5^ |  | 0.56 (0.12) | 5x10^-3^ |  | 4x10^-6^ |  | 0.47 (0.08) | 5x10^-6^ |  | 0.83 (0.11) | 0.13 |
| rs8060239 | 10019809 | 0.64 (0.07) | 5x10^-5^ |  | 0.50 (0.12) | 3x10^-3^ |  | 2x10^-6^ |  | 0.42 (0.08) | 2x10^-6^ |  | 0.82 (0.11) | 0.17 |
| rs17670766 | 10020926 | 0.67 (0.07) | 6x10^-5^ |  | 0.55 (0.12) | 5x10^-3^ |  | 5x10^-6^ |  | 0.47 (0.08) | 5x10^-6^ |  | 0.83 (0.11) | 0.14 |
| rs17569940 | 10021840 | 0.67 (0.07) | 5x10^-5^ |  | 0.56 (0.12) | 5x10^-3^ |  | 4x10^-6^ |  | 0.47 (0.08) | 5x10^-6^ |  | 0.83 (0.11) | 0.14 |
| rs1827197 | 10022865 | 0.67 (0.07) | 5x10^-5^ |  | 0.56 (0.12) | 5x10^-3^ |  | 4x10^-6^ |  | 0.47 (0.08) | 5x10^-6^ |  | 0.82 (0.11) | 0.13 |
| 16-10025128 | 10025128 | 0.67 (0.07) | 5x10^-5^ |  | 0.56 (0.12) | 6x10^-3^ |  | 4x10^-6^ |  | 0.47 (0.08) | 5x10^-6^ |  | 0.82 (0.11) | 0.12 |
| rs7193123 | 10030548 | 0.67 (0.07) | 5x10^-5^ |  | 0.57 (0.12) | 7x10^-3^ |  | 5x10^-6^ |  | 0.47 (0.08) | 6x10^-6^ |  | 0.82 (0.10) | 0.11 |
| 16-10037753 | 10037753 | 0.67 (0.07) | 4x10^-5^ |  | 0.56 (0.12) | 6x10^-3^ |  | 4x10^-6^ |  | 0.47 (0.08) | 5x10^-6^ |  | 0.82 (0.10) | 0.12 |
| 16-10040481 | 10040481 | 0.67 (0.07) | 4x10^-5^ |  | 0.56 (0.12) | 6x10^-3^ |  | 4x10^-6^ |  | 0.47 (0.08) | 5x10^-6^ |  | 0.82 (0.10) | 0.12 |
| 16-10041339 | 10041339 | 0.67 (0.07) | 4x10^-5^ |  | 0.56 (0.12) | 6x10^-3^ |  | 4x10^-6^ |  | 0.47 (0.08) | 5x10^-6^ |  | 0.82 (0.11) | 0.12 |
| 16-10043792 | 10043792 | 0.67 (0.07) | 4x10^-5^ |  | 0.56 (0.12) | 6x10^-3^ |  | 4x10^-6^ |  | 0.47 (0.08) | 5x10^-6^ |  | 0.82 (0.11) | 0.12 |
| 16-10044183 | 10044183 | 0.60 (0.07) | 4x10^-6^ |  | 0.56 (0.13) | 0.01 |  | 7x10^-7^ |  | 0.42 (0.08) | 3x10^-6^ |  | 0.74 (0.10) | 0.03 |
| 16-10044321 | 10044321 | 0.67 (0.07) | 4x10^-5^ |  | 0.56 (0.12) | 6x10^-3^ |  | 4x10^-6^ |  | 0.47 (0.08) | 5x10^-6^ |  | 0.82 (0.11) | 0.12 |
| 16-10044670 | 10044670 | 0.67 (0.07) | 4x10^-5^ |  | 0.56 (0.12) | 6x10^-3^ |  | 4x10^-6^ |  | 0.47 (0.08) | 5x10^-6^ |  | 0.82 (0.11) | 0.12 |
| rs7193471 | 10045134 | 0.67 (0.07) | 4x10^-5^ |  | 0.56 (0.12) | 6x10^-3^ |  | 4x10^-6^ |  | 0.47 (0.08) | 5x10^-6^ |  | 0.82 (0.11) | 0.12 |
| 16-10048254 | 10048254 | 0.66 (0.07) | 4x10^-5^ |  | 0.56 (0.12) | 5x10^-3^ |  | 3x10^-6^ |  | 0.47 (0.08) | 4x10^-6^ |  | 0.82 (0.11) | 0.13 |
| rs13332501 | 10048640 | 0.67 (0.07) | 4x10^-5^ |  | 0.56 (0.12) | 5x10^-3^ |  | 3x10^-6^ |  | 0.46 (0.08) | 4x10^-6^ |  | 0.82 (0.11) | 0.13 |
| 16-10048787 | 10048787 | 0.69 (0.07) | 1x10^-4^ |  | 0.53 (0.11) | 2x10^-3^ |  | 3x10^-6^ |  | 0.47 (0.07) | 2x10^-6^ |  | 0.87 (0.11) | 0.25 |
| 16-10049656 | 10049656 | 0.67 (0.07) | 4x10^-5^ |  | 0.56 (0.12) | 5x10^-3^ |  | 3x10^-6^ |  | 0.46 (0.08) | 4x10^-6^ |  | 0.82 (0.11) | 0.13 |
| rs28528871 | 10049884 | 0.67 (0.07) | 4x10^-5^ |  | 0.55 (0.12) | 5x10^-3^ |  | 3x10^-6^ |  | 0.46 (0.08) | 4x10^-6^ |  | 0.82 (0.11) | 0.13 |
| rs7190321 | 10051520 | 0.67 (0.07) | 5x10^-5^ |  | 0.55 (0.11) | 4x10^-3^ |  | 3x10^-6^ |  | 0.46 (0.08) | 4x10^-6^ |  | 0.83 (0.11) | 0.14 |
| 16-10052589 | 10052589 | 0.67 (0.07) | 5x10^-5^ |  | 0.55 (0.11) | 4x10^-3^ |  | 3x10^-6^ |  | 0.47 (0.08) | 3x10^-6^ |  | 0.83 (0.11) | 0.15 |
| rs28491487 | 10052697 | 0.67 (0.07) | 7x10^-5^ |  | 0.55 (0.12) | 5x10^-3^ |  | 6x10^-6^ |  | 0.47 (0.08) | 6x10^-6^ |  | 0.83 (0.11) | 0.16 |
| rs8056683 | 10052710 | 0.67 (0.07) | 5X10^-5^ |  | 0.55 (0.11) | 4x10^-3^ |  | 4x10^-6^ |  | 0.44 (0.08) | 3x10^-6^ |  | 0.84 (0.11) | 0.14 |
| rs8061963 | 10052788 | 0.67 (0.07) | 5x10^-5^ |  | 0.55 (0.11) | 4x10^-3^ |  | 3x10^-6^ |  | 0.47 (0.08) | 3x10^-6^ |  | 0.83 (0.11) | 0.14 |
| rs8062320 | 10052970 | 0.67 (0.07) | 5x10^-5^ |  | 0.55 (0.11) | 4x10^-3^ |  | 3x10^-6^ |  | 0.47 (0.08) | 3x10^-6^ |  | 0.83 (0.11) | 0.14 |
| rs7203194 | 10053433 | 0.67 (0.07) | 5x10^-5^ |  | 0.55 (0.11) | 4x10^-3^ |  | 3x10^-6^ |  | 0.47 (0.08) | 3x10^-6^ |  | 0.83 (0.11) | 0.14 |
| rs8056514 | 10054493 | 0.67 (0.07) | 5x10^-5^ |  | 0.55 (0.11) | 4x10^-3^ |  | 3x10^-6^ |  | 0.46 (0.08) | 3x10^-6^ |  | 0.83 (0.11) | 0.14 |
| rs7200649 | 10054977 | 0.67 (0.07) | 5x10^-5^ |  | 0.55 (0.11) | 4x10^-3^ |  | 3x10^-6^ |  | 0.46 (0.08) | 3x10^-6^ |  | 0.83 (0.11) | 0.14 |
| rs7206296 | 10054979 | 0.67 (0.07) | 5x10^-5^ |  | 0.55 (0.11) | 4x10^-3^ |  | 3x10^-6^ |  | 0.46 (0.08) | 3x10^-6^ |  | 0.83 (0.11) | 0.14 |
| 16-10055961 | 10055961 | 0.67 (0.07) | 5x10^-5^ |  | 0.55 (0.11) | 4x10^-3^ |  | 3x10^-6^ |  | 0.46 (0.08) | 3x10^-6^ |  | 0.83 (0.11) | 0.14 |
| rs13335102 | 10056129 | 0.67 (0.07) | 5x10^-5^ |  | 0.55 (0.11) | 4x10^-3^ |  | 3x10^-6^ |  | 0.46 (0.08) | 3x10^-6^ |  | 0.83 (0.11) | 0.14 |
| 16-10056132 | 10056132 | 0.67 (0.07) | 5x10^-5^ |  | 0.55 (0.11) | 4x10^-3^ |  | 3x10^-6^ |  | 0.46 (0.08) | 3x10^-6^ |  | 0.83 (0.11) | 0.14 |
| rs58834898 | 10056278 | 0.67 (0.07) | 5x10^-5^ |  | 0.55 (0.11) | 4x10^-3^ |  | 3x10^-6^ |  | 0.46 (0.08) | 3x10^-6^ |  | 0.83 (0.11) | 0.14 |
| rs12102500 | 10056680 | 0.67 (0.07) | 5x10^-5^ |  | 0.55 (0.11) | 4x10^-3^ |  | 3x10^-6^ |  | 0.46 (0.08) | 3x10^-6^ |  | 0.83 (0.11) | 0.14 |
| rs12102636 | 10056729 | 0.67 (0.07) | 5x10^-5^ |  | 0.55 (0.11) | 4x10^-3^ |  | 3x10^-6^ |  | 0.46 (0.08) | 3x10^-6^ |  | 0.83 (0.11) | 0.14 |
| rs9925707 | 10057268 | 0.67 (0.07) | 5x10^-5^ |  | 0.55 (0.11) | 4x10^-3^ |  | 3x10^-6^ |  | 0.46 (0.08) | 3x10^-6^ |  | 0.83 (0.11) | 0.14 |
| rs9927914 | 10057354 | 0.67 (0.07) | 5x10^-5^ |  | 0.55 (0.11) | 4x10^-3^ |  | 3x10^-6^ |  | 0.46 (0.08) | 3x10^-6^ |  | 0.83 (0.11) | 0.14 |
| rs9925801 | 10057393 | 0.67 (0.07) | 5x10^-5^ |  | 0.55 (0.11) | 4x10^-3^ |  | 3x10^-6^ |  | 0.46 (0.08) | 3x10^-6^ |  | 0.83 (0.11) | 0.14 |
| rs9927926 | 10057405 | 0.67 (0.07) | 5X10^-5^ |  | 0.55 (0.11) | 4x10^-3^ |  | 4x10^-6^ |  | 0.44 (0.08) | 3x10^-6^ |  | 0.84 (0.11) | 0.14 |
| rs1463128 | 10057535 | 0.67 (0.07) | 5x10^-5^ |  | 0.55 (0.11) | 4x10^-3^ |  | 3x10^-6^ |  | 0.46 (0.08) | 3x10^-6^ |  | 0.83 (0.11) | 0.15 |
| rs9937970 | 10057671 | 0.67 (0.07) | 5x10^-5^ |  | 0.55 (0.11) | 4x10^-3^ |  | 3x10^-6^ |  | 0.46 (0.08) | 3x10^-6^ |  | 0.83 (0.11) | 0.15 |
| 16-10061092 | 10061092 | 0.67 (0.07) | 5x10^-5^ |  | 0.54 (0.11) | 3x10^-3^ |  | 2x10^-6^ |  | 0.46 (0.08) | 2x10^-6^ |  | 0.84 (0.11) | 0.18 |
| rs7194796 | 10061139 | 0.67 (0.07) | 5x10^-5^ |  | 0.54 (0.11) | 3x10^-3^ |  | 2x10^-6^ |  | 0.46 (0.08) | 2x10^-6^ |  | 0.84 (0.11) | 0.18 |
| rs7198175 | 10061667 | 0.67 (0.07) | 5x10^-5^ |  | 0.54 (0.11) | 3x10^-3^ |  | 2x10^-6^ |  | 0.46 (0.08) | 2x10^-6^ |  | 0.84 (0.11) | 0.18 |
| rs17670977 | 10062772 | 0.67 (0.07) | 5x10^-5^ |  | 0.54 (0.11) | 3x10^-3^ |  | 2x10^-6^ |  | 0.46 (0.08) | 2x10^-6^ |  | 0.84 (0.11) | 0.18 |
| 16-10063369 | 10063369 | 0.67 (0.07) | 5x10^-5^ |  | 0.54 (0.11) | 2x10^-3^ |  | 2x10^-6^ |  | 0.46 (0.08) | 2x10^-6^ |  | 0.84 (0.11) | 0.18 |
| rs7189360 | 10063496 | 0.67 (0.07) | 5x10^-5^ |  | 0.54 (0.11) | 2x10^-3^ |  | 2x10^-6^ |  | 0.46 (0.08) | 2x10^-6^ |  | 0.84 (0.11) | 0.18 |
| rs7188122 | 10063546 | 0.67 (0.07) | 5x10^-5^ |  | 0.54 (0.11) | 2x10^-3^ |  | 2x10^-6^ |  | 0.46 (0.08) | 2x10^-6^ |  | 0.84 (0.11) | 0.18 |
| rs9935354 | 10064292 | 0.67 (0.07) | 5x10^-5^ |  | 0.53 (0.11) | 2x10^-3^ |  | 2x10^-6^ |  | 0.46 (0.08) | 2x10^-6^ |  | 0.84 (0.11) | 0.18 |
| rs8043864 | 10064430 | 0.67 (0.07) | 5x10^-5^ |  | 0.53 (0.11) | 2x10^-3^ |  | 2x10^-6^ |  | 0.46 (0.08) | 2x10^-6^ |  | 0.84 (0.11) | 0.18 |
| rs9922383 | 10065179 | 0.67 (0.07) | 5x10^-5^ |  | 0.53 (0.11) | 2x10^-3^ |  | 2x10^-6^ |  | 0.45 (0.08) | 2x10^-6^ |  | 0.84 (0.11) | 0.18 |
| rs9922460 | 10065294 | 0.67 (0.07) | 4x10^-5^ |  | 0.53 (0.11) | 2x10^-3^ |  | 2x10^-6^ |  | 0.46 (0.08) | 2x10^-6^ |  | 0.84 (0.11) | 0.18 |
| rs9935774 | 10065592 | 0.67 (0.07) | 5x10^-5^ |  | 0.53 (0.11) | 2x10^-3^ |  | 2x10^-6^ |  | 0.46 (0.07) | 2x10^-6^ |  | 0.85 (0.11) | 0.19 |
| rs9938061 | 10065671 | 0.67 (0.07) | 4x10^-5^ |  | 0.53 (0.11) | 2x10^-3^ |  | 2x10^-6^ |  | 0.46 (0.08) | 2x10^-6^ |  | 0.84 (0.11) | 0.18 |
| rs7197200 | 10065921 | 0.67 (0.07) | 4x10^-5^ |  | 0.53 (0.11) | 2x10^-3^ |  | 2x10^-6^ |  | 0.46 (0.08) | 2x10^-6^ |  | 0.84 (0.11) | 0.18 |
| 16-10066267 | 10066267 | 0.67 (0.07) | 4x10^-5^ |  | 0.53 (0.11) | 2x10^-3^ |  | 2x10^-6^ |  | 0.46 (0.08) | 2x10^-6^ |  | 0.84 (0.11) | 0.18 |
| rs7192622 | 10066343 | 0.67 (0.07) | 4x10^-5^ |  | 0.53 (0.11) | 2x10^-3^ |  | 2x10^-6^ |  | 0.46 (0.08) | 2x10^-6^ |  | 0.84 (0.11) | 0.18 |
| 16-10066691 | 10066691 | 0.67 (0.07) | 4x10^-5^ |  | 0.53 (0.11) | 2x10^-3^ |  | 2x10^-6^ |  | 0.46 (0.08) | 2x10^-6^ |  | 0.84 (0.11) | 0.18 |
| rs1448261 | 10068325 | 0.67 (0.07) | 4x10^-5^ |  | 0.53 (0.11) | 2x10^-3^ |  | 2x10^-6^ |  | 0.46 (0.08) | 2x10^-6^ |  | 0.84 (0.11) | 0.18 |
| 16-10068351 | 10068351 | 0.67 (0.07) | 4x10^-5^ |  | 0.54 (0.11) | 2x10^-3^ |  | 2x10^-6^ |  | 0.46 (0.08) | 2x10^-6^ |  | 0.84 (0.11) | 0.18 |
| rs1448262 | 10068370 | 0.67 (0.07) | 4x10^-5^ |  | 0.54 (0.11) | 2x10^-3^ |  | 2x10^-6^ |  | 0.46 (0.08) | 2x10^-6^ |  | 0.84 (0.11) | 0.18 |
| rs7188081 | 10068435 | 0.67 (0.07) | 4x10^-5^ |  | 0.54 (0.11) | 2x10^-3^ |  | 2x10^-6^ |  | 0.46 (0.08) | 2x10^-6^ |  | 0.84 (0.11) | 0.18 |
| rs7188291 | 10068537 | 0.67 (0.07) | 4x10^-5^ |  | 0.54 (0.11) | 2x10^-3^ |  | 2x10^-6^ |  | 0.46 (0.08) | 2x10^-6^ |  | 0.84 (0.11) | 0.18 |
| rs17671033 | 10068727 | 0.68 (0.07) | 7X10^-5^ |  | 0.53 (0.11) | 2x10^-3^ |  | 4x10^-6^ |  | 0.44 (0.08) | 3x10^-6^ |  | 0.85 (0.11) | 0.2 |
| rs9933478 | 10068753 | 0.67 (0.07) | 4x10^-5^ |  | 0.54 (0.11) | 2x10^-3^ |  | 2x10^-6^ |  | 0.46 (0.08) | 2x10^-6^ |  | 0.84 (0.11) | 0.18 |
| rs13331457 | 10069136 | 0.67 (0.07) | 4x10^-5^ |  | 0.53 (0.11) | 2x10^-3^ |  | 2x10^-6^ |  | 0.46 (0.07) | 2x10^-6^ |  | 0.84 (0.11) | 0.18 |
| rs28581562 | 10069342 | 0.67 (0.07) | 4x10^-5^ |  | 0.53 (0.11) | 2x10^-3^ |  | 2x10^-6^ |  | 0.46 (0.07) | 2x10^-6^ |  | 0.84 (0.11) | 0.18 |
| rs28465345 | 10069439 | 0.67 (0.07) | 4x10^-5^ |  | 0.53 (0.11) | 2x10^-3^ |  | 2x10^-6^ |  | 0.46 (0.07) | 2x10^-6^ |  | 0.84 (0.11) | 0.18 |
| rs28375797 | 10069497 | 0.67 (0.07) | 4x10^-5^ |  | 0.53 (0.11) | 2x10^-3^ |  | 2x10^-6^ |  | 0.46 (0.07) | 2x10^-6^ |  | 0.84 (0.11) | 0.18 |
| 16-10070145 | 10070145 | 0.67 (0.07) | 4x10^-5^ |  | 0.53 (0.11) | 2x10^-3^ |  | 2x10^-6^ |  | 0.46 (0.07) | 2x10^-6^ |  | 0.84 (0.11) | 0.18 |
| rs8057034 | 10070452 | 0.67 (0.07) | 5x10^-5^ |  | 0.53 (0.11) | 2x10^-3^ |  | 2x10^-6^ |  | 0.46 (0.07) | 2x10^-6^ |  | 0.84 (0.11) | 0.18 |
| rs8059513 | 10070729 | 0.67 (0.07) | 7x10^-5^ |  | 0.53 (0.11) | 3x10^-3^ |  | 3x10^-6^ |  | 0.46 (0.08) | 3x10^-6^ |  | 0.85 (0.11) | 0.2 |
| rs8059547 | 10070795 | 0.67 (0.07) | 5x10^-5^ |  | 0.53 (0.11) | 2x10^-3^ |  | 2x10^-6^ |  | 0.46 (0.07) | 2x10^-6^ |  | 0.84 (0.11) | 0.18 |
| 16-10070978 | 10070978 | 0.67 (0.07) | 5x10^-5^ |  | 0.53 (0.11) | 2x10^-3^ |  | 2x10^-6^ |  | 0.45 (0.07) | 2x10^-6^ |  | 0.84 (0.11) | 0.18 |
| 16-10071089 | 10071089 | 0.67 (0.07) | 5x10^-5^ |  | 0.53 (0.11) | 2x10^-3^ |  | 2x10^-6^ |  | 0.45 (0.07) | 2x10^-6^ |  | 0.85 (0.11) | 0.19 |
| rs9930396 | 10071278 | 0.67 (0.07) | 5x10^-5^ |  | 0.53 (0.11) | 2x10^-3^ |  | 2x10^-6^ |  | 0.45 (0.07) | 2x10^-6^ |  | 0.85 (0.11) | 0.19 |
| 16-10071764 | 10071764 | 0.67 (0.07) | 5x10^-5^ |  | 0.53 (0.11) | 2x10^-3^ |  | 2x10^-6^ |  | 0.45 (0.07) | 1x10^-6^ |  | 0.85 (0.11) | 0.19 |
| 16-10071991 | 10071991 | 0.67 (0.07) | 5x10^-5^ |  | 0.53 (0.11) | 2x10^-3^ |  | 1x10^-6^ |  | 0.45 (0.07) | 1x10^-6^ |  | 0.85 (0.11) | 0.19 |
| 16-10071996 | 10071996 | 0.67 (0.07) | 5x10^-5^ |  | 0.53 (0.11) | 2x10^-3^ |  | 1x10^-6^ |  | 0.45 (0.07) | 1x10^-6^ |  | 0.85 (0.11) | 0.19 |
| rs9933111 | 10072100 | 0.67 (0.07) | 5X10^-5^ |  | 0.53 (0.11) | 2x10^-3^ |  | 2x10^-6^ |  | 0.45 (0.07) | 1x10^-6^ |  | 0.85 (0.11) | 0.2 |
| rs9922871 | 10072317 | 0.67 (0.07) | 5x10^-5^ |  | 0.53 (0.11) | 2x10^-3^ |  | 1x10^-6^ |  | 0.45 (0.07) | 1x10^-6^ |  | 0.85 (0.11) | 0.19 |
| rs9922338 | 10072329 | 0.66 (0.07) | 4x10^-5^ |  | 0.52 (0.11) | 2x10^-3^ |  | 1x10^-6^ |  | 0.44 (0.07) | 1x10^-6^ |  | 0.84 (0.11) | 0.19 |
| rs9302409 | 10072531 | 0.67 (0.07) | 5x10^-5^ |  | 0.53 (0.11) | 2x10^-3^ |  | 1x10^-6^ |  | 0.45 (0.07) | 1x10^-6^ |  | 0.85 (0.11) | 0.19 |
| 16-10072565 | 10072565 | 0.67 (0.07) | 5x10^-5^ |  | 0.53 (0.11) | 2x10^-3^ |  | 1x10^-6^ |  | 0.45 (0.07) | 1x10^-6^ |  | 0.85 (0.11) | 0.19 |
| rs17570290 | 10072647 | 0.67 (0.07) | 5x10^-5^ |  | 0.53 (0.11) | 2x10^-3^ |  | 1x10^-6^ |  | 0.45 (0.07) | 1x10^-6^ |  | 0.85 (0.11) | 0.19 |
| rs10518152 | 10072788 | 0.67 (0.07) | 5x10^-5^ |  | 0.53 (0.11) | 2x10^-3^ |  | 1x10^-6^ |  | 0.45 (0.07) | 1x10^-6^ |  | 0.85 (0.11) | 0.19 |
| rs10518151 | 10072820 | 0.65 (0.07) | 6x10^-5^ |  | 0.51 (0.11) | 3x10^-3^ |  | 3x10^-6^ |  | 0.43 (0.08) | 2x10^-6^ |  | 0.83 (0.12) | 0.19 |
| 16-10073124 | 10073124 | 0.67 (0.07) | 5x10^-5^ |  | 0.53 (0.11) | 2x10^-3^ |  | 1x10^-6^ |  | 0.45 (0.07) | 1x10^-6^ |  | 0.85 (0.11) | 0.19 |
| rs13332018 | 10073171 | 0.67 (0.07) | 5x10^-5^ |  | 0.53 (0.11) | 2x10^-3^ |  | 1x10^-6^ |  | 0.45 (0.07) | 1x10^-6^ |  | 0.85 (0.11) | 0.19 |
| rs9938467 | 10073412 | 0.67 (0.07) | 5x10^-5^ |  | 0.53 (0.11) | 2x10^-3^ |  | 1x10^-6^ |  | 0.45 (0.07) | 1x10^-6^ |  | 0.85 (0.11) | 0.19 |
| rs8061018 | 10073903 | 0.67 (0.07) | 5x10^-5^ |  | 0.53 (0.11) | 2x10^-3^ |  | 1x10^-6^ |  | 0.45 (0.07) | 1x10^-6^ |  | 0.85 (0.11) | 0.19 |
| rs9927648 | 10074002 | 0.67 (0.07) | 5x10^-5^ |  | 0.53 (0.11) | 2x10^-3^ |  | 1x10^-6^ |  | 0.45 (0.07) | 1x10^-6^ |  | 0.85 (0.11) | 0.19 |
| 16-10074078 | 10074078 | 0.67 (0.07) | 5x10^-5^ |  | 0.53 (0.11) | 2x10^-3^ |  | 1x10^-6^ |  | 0.45 (0.07) | 1x10^-6^ |  | 0.85 (0.11) | 0.19 |
| rs58797003 | 10074188 | 0.67 (0.07) | 5x10^-5^ |  | 0.53 (0.11) | 2x10^-3^ |  | 1x10^-6^ |  | 0.45 (0.07) | 1x10^-6^ |  | 0.85 (0.11) | 0.19 |
| rs59568026 | 10074278 | 0.67 (0.07) | 5x10^-5^ |  | 0.53 (0.11) | 2x10^-3^ |  | 1x10^-6^ |  | 0.45 (0.07) | 1x10^-6^ |  | 0.85 (0.11) | 0.19 |
| 16-10074282 | 10074282 | 0.67 (0.07) | 5x10^-5^ |  | 0.53 (0.11) | 2x10^-3^ |  | 1x10^-6^ |  | 0.45 (0.07) | 1x10^-6^ |  | 0.85 (0.11) | 0.19 |
| 16-10074631 | 10074631 | 0.67 (0.07) | 5x10^-5^ |  | 0.53 (0.11) | 2x10^-3^ |  | 1x10^-6^ |  | 0.45 (0.07) | 1x10^-6^ |  | 0.85 (0.11) | 0.19 |
| 16-10074643 | 10074643 | 0.67 (0.07) | 5x10^-5^ |  | 0.53 (0.11) | 2x10^-3^ |  | 1x10^-6^ |  | 0.45 (0.07) | 1x10^-6^ |  | 0.85 (0.11) | 0.19 |
| rs61224814 | 10074756 | 0.67 (0.07) | 5x10^-5^ |  | 0.53 (0.11) | 2x10^-3^ |  | 1x10^-6^ |  | 0.45 (0.07) | 1x10^-6^ |  | 0.85 (0.11) | 0.19 |
| rs60950183 | 10074799 | 0.67 (0.07) | 5x10^-5^ |  | 0.53 (0.11) | 2x10^-3^ |  | 1x10^-6^ |  | 0.45 (0.07) | 1x10^-6^ |  | 0.85 (0.11) | 0.19 |
| 16-10074810 | 10074810 | 0.67 (0.07) | 5x10^-5^ |  | 0.53 (0.11) | 2x10^-3^ |  | 1x10^-6^ |  | 0.45 (0.07) | 1x10^-6^ |  | 0.85 (0.11) | 0.19 |
| 16-10074932 | 10074932 | 0.67 (0.07) | 5x10^-5^ |  | 0.53 (0.11) | 2x10^-3^ |  | 1x10^-6^ |  | 0.45 (0.07) | 1x10^-6^ |  | 0.85 (0.11) | 0.19 |
| 16-10074942 | 10074942 | 0.67 (0.07) | 5x10^-5^ |  | 0.53 (0.11) | 2x10^-3^ |  | 1x10^-6^ |  | 0.45 (0.07) | 1x10^-6^ |  | 0.85 (0.11) | 0.19 |
| 16-10075021 | 10075021 | 0.67 (0.07) | 5x10^-5^ |  | 0.53 (0.11) | 2x10^-3^ |  | 1x10^-6^ |  | 0.45 (0.07) | 1x10^-6^ |  | 0.85 (0.11) | 0.19 |
| rs56666352 | 10075139 | 0.67 (0.07) | 5x10^-5^ |  | 0.53 (0.11) | 2x10^-3^ |  | 1x10^-6^ |  | 0.45 (0.07) | 1x10^-6^ |  | 0.85 (0.11) | 0.19 |
| 16-10075411 | 10075411 | 0.67 (0.07) | 5x10^-5^ |  | 0.53 (0.11) | 2x10^-3^ |  | 1x10^-6^ |  | 0.45 (0.07) | 1x10^-6^ |  | 0.85 (0.11) | 0.19 |
| 16-10075461 | 10075461 | 0.67 (0.07) | 5x10^-5^ |  | 0.53 (0.11) | 2x10^-3^ |  | 1x10^-6^ |  | 0.45 (0.07) | 1x10^-6^ |  | 0.85 (0.11) | 0.19 |
| 16-10075477 | 10075477 | 0.67 (0.07) | 5x10^-5^ |  | 0.53 (0.11) | 2x10^-3^ |  | 1x10^-6^ |  | 0.45 (0.07) | 1x10^-6^ |  | 0.85 (0.11) | 0.19 |
| rs12102832 | 10075633 | 0.67 (0.07) | 5x10^-5^ |  | 0.53 (0.11) | 2x10^-3^ |  | 1x10^-6^ |  | 0.45 (0.07) | 1x10^-6^ |  | 0.85 (0.11) | 0.19 |
| 16-10075722 | 10075722 | 0.67 (0.07) | 5x10^-5^ |  | 0.53 (0.11) | 2x10^-3^ |  | 1x10^-6^ |  | 0.45 (0.07) | 1x10^-6^ |  | 0.85 (0.11) | 0.19 |
| rs8062241 | 10075809 | 0.67 (0.07) | 5x10^-5^ |  | 0.53 (0.11) | 2x10^-3^ |  | 1x10^-6^ |  | 0.45 (0.07) | 1x10^-6^ |  | 0.85 (0.11) | 0.19 |
| rs8062624 | 10075998 | 0.67 (0.07) | 5x10^-5^ |  | 0.53 (0.11) | 2x10^-3^ |  | 1x10^-6^ |  | 0.45 (0.07) | 1x10^-6^ |  | 0.85 (0.11) | 0.19 |
| rs9931653 | 10076193 | 0.67 (0.07) | 5x10^-5^ |  | 0.53 (0.11) | 2x10^-3^ |  | 1x10^-6^ |  | 0.45 (0.07) | 1x10^-6^ |  | 0.85 (0.11) | 0.2 |
| rs10518150 | 10076257 | 0.67 (0.07) | 5x10^-5^ |  | 0.53 (0.11) | 2x10^-3^ |  | 2x10^-6^ |  | 0.45 (0.07) | 1x10^-6^ |  | 0.85 (0.11) | 0.2 |
| rs9933945 | 10076487 | 0.67 (0.07) | 5x10^-5^ |  | 0.53 (0.11) | 2x10^-3^ |  | 2x10^-6^ |  | 0.45 (0.07) | 1x10^-6^ |  | 0.85 (0.11) | 0.2 |
| rs10518149 | 10076563 | 0.67 (0.07) | 5x10^-5^ |  | 0.53 (0.11) | 2x10^-3^ |  | 2x10^-6^ |  | 0.45 (0.07) | 1x10^-6^ |  | 0.85 (0.11) | 0.2 |
| rs28634372 | 10076780 | 0.67 (0.07) | 5x10^-5^ |  | 0.53 (0.11) | 2x10^-3^ |  | 2x10^-6^ |  | 0.45 (0.07) | 1x10^-6^ |  | 0.85 (0.11) | 0.2 |
| rs60276136 | 10077091 | 0.67 (0.07) | 5x10^-5^ |  | 0.53 (0.11) | 2x10^-3^ |  | 2x10^-6^ |  | 0.45 (0.07) | 1x10^-6^ |  | 0.85 (0.11) | 0.2 |
| rs28540790 | 10077145 | 0.67 (0.07) | 5x10^-5^ |  | 0.53 (0.11) | 2x10^-3^ |  | 2x10^-6^ |  | 0.45 (0.07) | 1x10^-6^ |  | 0.85 (0.11) | 0.2 |
| rs28574279 | 10077172 | 0.67 (0.07) | 5x10^-5^ |  | 0.53 (0.11) | 2x10^-3^ |  | 2x10^-6^ |  | 0.45 (0.07) | 1x10^-6^ |  | 0.85 (0.11) | 0.2 |
| 16-10077221 | 10077221 | 0.67 (0.07) | 5x10^-5^ |  | 0.53 (0.11) | 2x10^-3^ |  | 2x10^-6^ |  | 0.45 (0.07) | 1x10^-6^ |  | 0.85 (0.11) | 0.2 |
| rs57587803 | 10077247 | 0.67 (0.07) | 5x10^-5^ |  | 0.53 (0.11) | 2x10^-3^ |  | 2x10^-6^ |  | 0.45 (0.07) | 1x10^-6^ |  | 0.85 (0.11) | 0.2 |
| rs60864732 | 10077257 | 0.67 (0.07) | 5x10^-5^ |  | 0.53 (0.11) | 2x10^-3^ |  | 2x10^-6^ |  | 0.45 (0.07) | 1x10^-6^ |  | 0.85 (0.11) | 0.2 |
| rs58861142 | 10077314 | 0.67 (0.07) | 5x10^-5^ |  | 0.53 (0.11) | 2x10^-3^ |  | 2x10^-6^ |  | 0.45 (0.07) | 1x10^-6^ |  | 0.85 (0.11) | 0.2 |
| rs16956843 | 10077466 | 0.67 (0.07) | 5x10^-5^ |  | 0.53 (0.11) | 2x10^-3^ |  | 2x10^-6^ |  | 0.45 (0.07) | 1x10^-6^ |  | 0.85 (0.11) | 0.2 |
| 16-10077488 | 10077488 | 0.67 (0.07) | 5x10^-5^ |  | 0.53 (0.11) | 2x10^-3^ |  | 2x10^-6^ |  | 0.45 (0.07) | 1x10^-6^ |  | 0.85 (0.11) | 0.2 |
| rs61686802 | 10077552 | 0.67 (0.07) | 5x10^-5^ |  | 0.53 (0.11) | 2x10^-3^ |  | 2x10^-6^ |  | 0.45 (0.07) | 1x10^-6^ |  | 0.85 (0.11) | 0.2 |
| 16-10077618 | 10077618 | 0.67 (0.07) | 5x10^-5^ |  | 0.53 (0.11) | 2x10^-3^ |  | 2x10^-6^ |  | 0.45 (0.07) | 1x10^-6^ |  | 0.85 (0.11) | 0.2 |
| 16-10077656 | 10077656 | 0.67 (0.07) | 5x10^-5^ |  | 0.53 (0.11) | 2x10^-3^ |  | 2x10^-6^ |  | 0.45 (0.07) | 1x10^-6^ |  | 0.85 (0.11) | 0.2 |
| rs13331030 | 10077692 | 0.67 (0.07) | 5x10^-5^ |  | 0.53 (0.11) | 2x10^-3^ |  | 2x10^-6^ |  | 0.45 (0.07) | 1x10^-6^ |  | 0.85 (0.11) | 0.2 |
| 16-10077704 | 10077704 | 0.67 (0.07) | 5x10^-5^ |  | 0.53 (0.11) | 2x10^-3^ |  | 2x10^-6^ |  | 0.45 (0.07) | 1x10^-6^ |  | 0.85 (0.11) | 0.2 |
| rs13331085 | 10077809 | 0.67 (0.07) | 5x10^-5^ |  | 0.53 (0.11) | 2x10^-3^ |  | 2x10^-6^ |  | 0.45 (0.07) | 1x10^-6^ |  | 0.85 (0.11) | 0.2 |
| rs13331088 | 10077819 | 0.67 (0.07) | 5x10^-5^ |  | 0.53 (0.11) | 2x10^-3^ |  | 2x10^-6^ |  | 0.45 (0.07) | 1x10^-6^ |  | 0.85 (0.11) | 0.2 |
| rs13331428 | 10077870 | 0.67 (0.07) | 5x10^-5^ |  | 0.53 (0.11) | 2x10^-3^ |  | 2x10^-6^ |  | 0.45 (0.07) | 1x10^-6^ |  | 0.85 (0.11) | 0.2 |
| rs13331461 | 10077948 | 0.67 (0.07) | 5x10^-5^ |  | 0.53 (0.11) | 2x10^-3^ |  | 2x10^-6^ |  | 0.45 (0.07) | 1x10^-6^ |  | 0.85 (0.11) | 0.2 |
| rs13331465 | 10077968 | 0.67 (0.07) | 5X10^-5^ |  | 0.53 (0.11) | 2x10^-3^ |  | 2x10^-6^ |  | 0.45 (0.07) | 1x10^-6^ |  | 0.85 (0.11) | 0.2 |
| rs13331168 | 10077998 | 0.67 (0.07) | 5x10^-5^ |  | 0.53 (0.11) | 2x10^-3^ |  | 2x10^-6^ |  | 0.45 (0.07) | 1x10^-6^ |  | 0.85 (0.11) | 0.2 |
| 16-10078003 | 10078003 | 0.67 (0.07) | 5x10^-5^ |  | 0.53 (0.11) | 2x10^-3^ |  | 2x10^-6^ |  | 0.45 (0.07) | 1x10^-6^ |  | 0.85 (0.11) | 0.2 |
| rs13331514 | 10078110 | 0.67 (0.07) | 5x10^-5^ |  | 0.53 (0.11) | 2x10^-3^ |  | 2x10^-6^ |  | 0.45 (0.07) | 1x10^-6^ |  | 0.85 (0.11) | 0.2 |
| rs13336632 | 10078155 | 0.67 (0.07) | 5X10^-5^ |  | 0.52 (0.11) | 2x10^-3^ |  | 2x10^-6^ |  | 0.45 (0.07) | 1x10^-6^ |  | 0.85 (0.11) | 0.2 |
| rs57737700 | 10078197 | 0.67 (0.07) | 5x10^-5^ |  | 0.53 (0.11) | 2x10^-3^ |  | 2x10^-6^ |  | 0.45 (0.07) | 1x10^-6^ |  | 0.85 (0.11) | 0.2 |
| rs13336710 | 10078329 | 0.67 (0.07) | 5x10^-5^ |  | 0.53 (0.11) | 2x10^-3^ |  | 2x10^-6^ |  | 0.45 (0.07) | 1x10^-6^ |  | 0.85 (0.11) | 0.2 |
| rs13332665 | 10078506 | 0.67 (0.07) | 5x10^-5^ |  | 0.53 (0.11) | 2x10^-3^ |  | 2x10^-6^ |  | 0.45 (0.07) | 1x10^-6^ |  | 0.85 (0.11) | 0.2 |
| rs13332699 | 10078588 | 0.67 (0.07) | 5x10^-5^ |  | 0.53 (0.11) | 2x10^-3^ |  | 2x10^-6^ |  | 0.45 (0.07) | 1x10^-6^ |  | 0.85 (0.11) | 0.2 |
| 16-10078887 | 10078887 | 0.67 (0.07) | 5x10^-5^ |  | 0.53 (0.11) | 2x10^-3^ |  | 2x10^-6^ |  | 0.45 (0.07) | 2x10^-6^ |  | 0.85 (0.11) | 0.2 |
| 16-10079158 | 10079158 | 0.67 (0.07) | 6x10^-5^ |  | 0.53 (0.11) | 2x10^-3^ |  | 2x10^-6^ |  | 0.45 (0.07) | 2x10^-6^ |  | 0.85 (0.11) | 0.2 |
| 16-10079160 | 10079160 | 0.67 (0.07) | 6x10^-5^ |  | 0.53 (0.11) | 2x10^-3^ |  | 2x10^-6^ |  | 0.45 (0.07) | 2x10^-6^ |  | 0.85 (0.11) | 0.2 |
| rs9940458 | 10079364 | 0.67 (0.07) | 6x10^-5^ |  | 0.53 (0.11) | 2x10^-3^ |  | 2x10^-6^ |  | 0.45 (0.07) | 2x10^-6^ |  | 0.85 (0.11) | 0.21 |
| rs2352741 | 10080206 | 0.68 (0.07) | 7x10^-5^ |  | 0.53 (0.11) | 2x10^-3^ |  | 2x10^-6^ |  | 0.46 (0.08) | 2x10^-6^ |  | 0.86 (0.11) | 0.22 |
| rs1375072 | 10080576 | 0.68 (0.07) | 8x10^-5^ |  | 0.53 (0.11) | 2x10^-3^ |  | 3x10^-6^ |  | 0.46 (0.08) | 2x10^-6^ |  | 0.86 (0.11) | 0.22 |
| rs7191991 | 10081191 | 0.68 (0.07) | 9x10^-5^ |  | 0.53 (0.11) | 2x10^-3^ |  | 3x10^-6^ |  | 0.46 (0.08) | 2x10^-6^ |  | 0.86 (0.11) | 0.22 |
| rs1448268 | 10082178 | 0.68 (0.07) | 9x10^-5^ |  | 0.53 (0.11) | 2x10^-3^ |  | 3x10^-6^ |  | 0.46 (0.08) | 3x10^-6^ |  | 0.86 (0.11) | 0.22 |
| rs1448270 | 10082819 | 0.68 (0.07) | 1X10^-4^ |  | 0.53 (0.11) | 2x10^-3^ |  | 5x10^-6^ |  | 0.47 (0.08) | 3x10^-6^ |  | 0.85 (0.11) | 0.24 |
| rs1448271 | 10082972 | 0.68 (0.07) | 9x10^-5^ |  | 0.53 (0.11) | 2x10^-3^ |  | 3x10^-6^ |  | 0.46 (0.08) | 3x10^-6^ |  | 0.86 (0.11) | 0.22 |
| rs1111537 | 10083078 | 0.68 (0.07) | 1x10^-4^ |  | 0.53 (0.11) | 2x10^-3^ |  | 4x10^-6^ |  | 0.46 (0.08) | 3x10^-6^ |  | 0.86 (0.11) | 0.23 |
| rs1375075 | 10083230 | 0.68 (0.07) | 8x10^-5^ |  | 0.53 (0.11) | 2x10^-3^ |  | 3x10^-6^ |  | 0.46 (0.08) | 3x10^-6^ |  | 0.86 (0.11) | 0.22 |
| rs1375076 | 10083232 | 0.68 (0.07) | 8x10^-5^ |  | 0.53 (0.11) | 2x10^-3^ |  | 3x10^-6^ |  | 0.46 (0.08) | 3x10^-6^ |  | 0.86 (0.11) | 0.22 |
| rs4780784 | 10083589 | 0.68 (0.07) | 8x10^-5^ |  | 0.53 (0.11) | 2x10^-3^ |  | 2x10^-6^ |  | 0.46 (0.08) | 2x10^-6^ |  | 0.85 (0.11) | 0.22 |
| rs1448272 | 10083636 | 0.68 (0.07) | 8x10^-5^ |  | 0.53 (0.11) | 2x10^-3^ |  | 2x10^-6^ |  | 0.46 (0.08) | 2x10^-6^ |  | 0.85 (0.11) | 0.22 |
| 16-10084653 | 10084653 | 0.67 (0.07) | 6x10^-5^ |  | 0.52 (0.11) | 2x10^-3^ |  | 2x10^-6^ |  | 0.45 (0.07) | 2x10^-6^ |  | 0.85 (0.11) | 0.21 |
| rs7199095 | 10085009 | 0.68 (0.07) | 7x10^-5^ |  | 0.49 (0.10) | 5x10^-4^ |  | 6x10^-7^ |  | 0.44 (0.07) | 6x10^-7^ |  | 0.88 (0.11) | 0.29 |
| rs7206094 | 10085011 | 0.68 (0.07) | 7x10^-5^ |  | 0.49 (0.10) | 5x10^-4^ |  | 6x10^-7^ |  | 0.44 (0.07) | 5x10^-7^ |  | 0.88 (0.11) | 0.29 |
| 16-10086160 | 10086160 | 0.70 (0.06) | 1x10^-4^ |  | 0.47 (0.09) | 1x10^-4^ |  | 2x10^-7^ |  | 0.43 (0.07) | 1x10^-7^ |  | 0.91 (0.11) | 0.43 |
| 16-10086580 | 10086580 | 0.70 (0.06) | 1x10^-4^ |  | 0.47 (0.09) | 1x10^-4^ |  | 2x10^-7^ |  | 0.43 (0.07) | 1x10^-7^ |  | 0.91 (0.11) | 0.43 |
| rs7201627 | 10087459 | 0.68 (0.07) | 6x10^-5^ |  | 0.49 (0.10) | 4x10^-4^ |  | 4x10^-7^ |  | 0.43 (0.07) | 4x10^-7^ |  | 0.87 (0.11) | 0.28 |
| 16-10089064 | 10089064 | 0.70 (0.07) | 2x10^-4^ |  | 0.47 (0.09) | 1x10^-4^ |  | 3x10^-7^ |  | 0.43 (0.07) | 2x10^-7^ |  | 0.92 (0.11) | 0.46 |
| 16-10090107 | 10090107 | 0.70 (0.06) | 9x10^-5^ |  | 0.46 (0.09) | 8x10^-5^ |  | 1x10^-7^ |  | 0.42 (0.07) | 7x10^-8^ |  | 0.91 (0.11) | 0.43 |
| 16-10092692 | 10092692 | 0.69 (0.06) | 7x10^-5^ |  | 0.46 (0.09) | 7x10^-5^ |  | 8x10^-8^ |  | 0.42 (0.07) | 5x10^-8^ |  | 0.91 (0.11) | 0.41 |
| 16-10093997 | 10093997 | 0.69 (0.06) | 7x10^-5^ |  | 0.46 (0.09) | 7x10^-5^ |  | 8x10^-8^ |  | 0.42 (0.07) | 5x10^-8^ |  | 0.91 (0.11) | 0.4 |
| 16-10094528 | 10094528 | 0.69 (0.06) | 7x10^-5^ |  | 0.46 (0.09) | 7x10^-5^ |  | 8x10^-8^ |  | 0.42 (0.07) | 5x10^-8^ |  | 0.91 (0.11) | 0.4 |
| rs17671178 | 10094708 | 0.69 (0.06) | 7x10^-5^ |  | 0.46 (0.09) | 7x10^-5^ |  | 8x10^-8^ |  | 0.42 (0.07) | 5x10^-8^ |  | 0.91 (0.11) | 0.4 |
| 16-10098086 | 10098086 | 0.71 (0.07) | 4x10^-4^ |  | 0.47 (0.10) | 2x10^-4^ |  | 1x10^-6^ |  | 0.44 (0.07) | 6x10^-7^ |  | 0.92 (0.11) | 0.49 |
| 16-10098493 | 10098493 | 0.71 (0.07) | 4x10^-4^ |  | 0.46 (0.10) | 2x10^-4^ |  | 1x10^-6^ |  | 0.43 (0.07) | 5x10^-7^ |  | 0.92 (0.11) | 0.52 |
| 16-10098724 | 10098724 | 0.70 (0.06) | 1x10^-4^ |  | 0.48 (0.09) | 2x10^-4^ |  | 3x10^-7^ |  | 0.44 (0.07) | 2x10^-7^ |  | 0.90 (0.11) | 0.39 |
| 16-10099982 | 10099982 | 0.70 (0.07) | 1x10^-4^ |  | 0.47 (0.09) | 1x10^-4^ |  | 3x10^-7^ |  | 0.43 (0.07) | 2x10^-7^ |  | 0.91 (0.11) | 0.43 |
| 16-10101465 | 10101465 | 0.69 (0.06) | 8x10^-5^ |  | 0.45 (0.09) | 6x10^-5^ |  | 7x10^-8^ |  | 0.41 (0.07) | 5x10^-8^ |  | 0.91 (0.11) | 0.43 |
| 16-10102124 | 10102124 | 0.69 (0.06) | 7x10^-5^ |  | 0.44 (0.09) | 4x10^-5^ |  | 5x10^-8^ |  | 0.41 (0.07) | 3x10^-8^ |  | 0.92 (0.11) | 0.46 |
| 16-10102229 | 10102229 | 0.69 (0.06) | 7x10^-5^ |  | 0.44 (0.09) | 4x10^-5^ |  | 5x10^-8^ |  | 0.41 (0.07) | 3x10^-8^ |  | 0.92 (0.11) | 0.46 |
| 16-10103787 | 10103787 | 0.69 (0.06) | 7x10^-5^ |  | 0.44 (0.09) | 4x10^-5^ |  | 5x10^-8^ |  | 0.41 (0.07) | 3x10^-8^ |  | 0.92 (0.11) | 0.46 |
| 16-10105921 | 10105921 | 0.69 (0.06) | 7x10^-5^ |  | 0.44 (0.09) | 4x10^-5^ |  | 5x10^-8^ |  | 0.41 (0.07) | 3x10^-8^ |  | 0.92 (0.11) | 0.46 |
| rs56275045 | 10108893 | 0.69 (0.06) | 6x10^-5^ |  | 0.45 (0.09) | 5x10^-5^ |  | 5x10^-8^ |  | 0.42 (0.07) | 4x10^-8^ |  | 0.91 (0.11) | 0.41 |
| 16-10109203 | 10109203 | 0.69 (0.06) | 6x10^-5^ |  | 0.45 (0.09) | 5x10^-5^ |  | 5x10^-8^ |  | 0.42 (0.07) | 4x10^-8^ |  | 0.91 (0.11) | 0.41 |
| 16-10109483 | 10109483 | 0.66 (0.06) | 3x10^-5^ |  | 0.47 (0.10) | 3x10^-4^ |  | 1x10^-7^ |  | 0.41 (0.07) | 1x10^-7^ |  | 0.86 (0.11) | 0.23 |
| 16-10110896 | 10110896 | 0.69 (0.06) | 6x10^-5^ |  | 0.45 (0.09) | 6x10^-5^ |  | 6x10^-8^ |  | 0.42 (0.07) | 4x10^-8^ |  | 0.91 (0.11) | 0.41 |
| rs11866570 | 10113676 | 0.74 (0.07) | 9X10^-4^ |  | 0.54 (0.11) | 2x10^-3^ |  | 3x10^-5^ |  | 0.51 (0.08) | 1x10^-5^ |  | 0.85 (0.11) | 0.45 |
| 16-10116251 | 10116251 | 0.66 (0.07) | 5x10^-5^ |  | 0.50 (0.11) | 1x10^-3^ |  | 9x10^-7^ |  | 0.43 (0.07) | 9x10^-7^ |  | 0.84 (0.11) | 0.18 |
| 16-10116566 | 10116566 | 0.66 (0.07) | 4x10^-5^ |  | 0.50 (0.11) | 1x10^-3^ |  | 8x10^-7^ |  | 0.43 (0.07) | 9x10^-7^ |  | 0.84 (0.11) | 0.18 |
| 16-10118795 | 10118795 | 0.66 (0.07) | 3x10^-5^ |  | 0.52 (0.11) | 2x10^-3^ |  | 6x10^-7^ |  | 0.44 (0.07) | 8x10^-7^ |  | 0.83 (0.11) | 0.14 |
| 16-10120657 | 10120657 | 0.66 (0.07) | 2x10^-5^ |  | 0.51 (0.11) | 1x10^-3^ |  | 5x10^-7^ |  | 0.43 (0.07) | 6x10^-7^ |  | 0.83 (0.10) | 0.14 |
| 16-10121144 | 10121144 | 0.66 (0.07) | 2x10^-5^ |  | 0.51 (0.11) | 1x10^-3^ |  | 5x10^-7^ |  | 0.43 (0.07) | 6x10^-7^ |  | 0.83 (0.10) | 0.14 |
| rs7196139 | 10121899 | 0.65 (0.07) | 2x10^-5^ |  | 0.51 (0.11) | 1x10^-3^ |  | 5x10^-7^ |  | 0.43 (0.07) | 6x10^-7^ |  | 0.83 (0.10) | 0.13 |
| 16-10122294 | 10122294 | 0.65 (0.06) | 2x10^-5^ |  | 0.52 (0.11) | 2x10^-3^ |  | 4x10^-7^ |  | 0.43 (0.07) | 6x10^-7^ |  | 0.82 (0.10) | 0.13 |
| 16-10123191 | 10123191 | 0.65 (0.07) | 2x10^-5^ |  | 0.51 (0.11) | 1x10^-3^ |  | 5x10^-7^ |  | 0.43 (0.07) | 7x10^-7^ |  | 0.83 (0.11) | 0.15 |
| 16-10123460 | 10123460 | 0.64 (0.07) | 2x10^-5^ |  | 0.53 (0.12) | 4x10^-3^ |  | 1x10^-6^ |  | 0.43 (0.08) | 2x10^-6^ |  | 0.80 (0.11) | 0.09 |
| rs1375068 | 10123958 | 0.66 (0.07) | 4x10^-5^ |  | 0.53 (0.11) | 2x10^-3^ |  | 1x10^-6^ |  | 0.45 (0.07) | 2x10^-6^ |  | 0.83 (0.11) | 0.15 |
| 16-10125149 | 10125149 | 0.67 (0.07) | 4x10^-5^ |  | 0.54 (0.11) | 3x10^-3^ |  | 2x10^-6^ |  | 0.46 (0.08) | 2x10^-6^ |  | 0.83 (0.10) | 0.14 |
| rs4782266 | 10125491 | 0.66 (0.07) | 5x10^-5^ |  | 0.53 (0.11) | 2x10^-3^ |  | 2x10^-6^ |  | 0.45 (0.08) | 2x10^-6^ |  | 0.84 (0.11) | 0.17 |
| rs56135508 | 10125997 | 0.67 (0.07) | 5x10^-5^ |  | 0.53 (0.11) | 2x10^-3^ |  | 2x10^-6^ |  | 0.45 (0.08) | 2x10^-6^ |  | 0.84 (0.11) | 0.17 |
| rs1448253 | 10128367 | 0.68 (0.07) | 7X10^-5^ |  | 0.55 (0.11) | 3x10^-3^ |  | 5x10^-6^ |  | 0.47 (0.08) | 4x10^-6^ |  | 0.84 (0.11) | 0.18 |
| 16-10128920 | 10128920 | 0.67 (0.07) | 6x10^-5^ |  | 0.55 (0.11) | 3x10^-3^ |  | 3x10^-6^ |  | 0.47 (0.08) | 3x10^-6^ |  | 0.84 (0.11) | 0.16 |
| rs9930364 | 10128984 | 0.67 (0.07) | 6x10^-5^ |  | 0.55 (0.11) | 3x10^-3^ |  | 3x10^-6^ |  | 0.47 (0.08) | 4x10^-6^ |  | 0.84 (0.11) | 0.16 |
| 16-10129181 | 10129181 | 0.67 (0.07) | 6x10^-5^ |  | 0.55 (0.11) | 4x10^-3^ |  | 4x10^-6^ |  | 0.47 (0.08) | 4x10^-6^ |  | 0.84 (0.11) | 0.15 |
| rs11645379 | 10129471 | 0.67 (0.07) | 8x10^-5^ |  | 0.54 (0.11) | 3x10^-3^ |  | 3x10^-6^ |  | 0.46 (0.08) | 3x10^-6^ |  | 0.84 (0.11) | 0.18 |
| rs6497716 | 10130309 | 0.68 (0.07) | 7x10^-5^ |  | 0.56 (0.11) | 4x10^-3^ |  | 5x10^-6^ |  | 0.47 (0.08) | 5x10^-6^ |  | 0.84 (0.10) | 0.15 |
| rs2197778 | 10130956 | 0.68 (0.07) | 8x10^-5^ |  | 0.56 (0.11) | 5x10^-3^ |  | 5x10^-6^ |  | 0.47 (0.08) | 5x10^-6^ |  | 0.84 (0.10) | 0.15 |
| 16-10132126 | 10132126 | 0.68 (0.07) | 9x10^-5^ |  | 0.56 (0.11) | 5x10^-3^ |  | 6x10^-6^ |  | 0.48 (0.08) | 6x10^-6^ |  | 0.84 (0.10) | 0.15 |
| rs1070479 | 10136873 | 0.69 (0.07) | 1x10^-4^ |  | 0.57 (0.12) | 0.01 |  | 9x10^-6^ |  | 0.49 (0.08) | 9x10^-6^ |  | 0.84 (0.10) | 0.16 |
